# Revision of *Varanus marathonensis* (Squamata, Varanidae) based on historical and new material: morphology, systematics, and paleobiogeography of the European monitor lizards

**DOI:** 10.1371/journal.pone.0207719

**Published:** 2018-12-05

**Authors:** Andrea Villa, Juan Abella, David M. Alba, Sergio Almécija, Arnau Bolet, George D. Koufos, Fabien Knoll, Àngel H. Luján, Jorge Morales, Josep M. Robles, Israel M. Sánchez, Massimo Delfino

**Affiliations:** 1 Dipartimento di Scienze della Terra, Università di Torino, Torino, Italy; 2 Universidad Estatal de la Peninsula de Santa Elena, La Libertad, Santa Elena, Ecuador; 3 Institut Català de Paleontologia Miquel Crusafont, Universitat Autònoma de Barcelona, Cerdanyola del Vallès, Barcelona, Spain; 4 Division of Anthropology, American Museum of Natural History, New York, United States of America; 5 New York Consortium in Evolutionary Primatology, New York, United States of America; 6 School of Earth Sciences, University of Bristol, Bristol, United Kingdom; 7 Laboratory of Geology and Paleontology, Department of Geology, Aristotle University of Thessaloniki, Thessaloniki, Greece; 8 Departamento de Paleobiología, Museo Nacional de Ciencias Naturales–Consejo Superior de Investigaciones Cientificas, Madrid, Spain; 9 ARAID–Fundación Conjunto Paleontológico de Teruel-Dinópolis, Teruel, Spain; 10 School of Earth & Environmental Sciences, University of Manchester, Manchester, United Kingdom; 11 Department of Geological Sciences, Faculty of Science, Masaryk University, Brno, Czech Republic; Ecole Normale Supérieure de Lyon, FRANCE

## Abstract

Monitor lizards (genus *Varanus*) inhabited Europe at least from the early Miocene to the Pleistocene. Their fossil record is limited to about 40 localities that have provided mostly isolated vertebrae. Due to the poor diagnostic value of these fossils, it was recently claimed that all the European species described prior to the 21^st^ century are not taxonomically valid and a new species, *Varanus amnhophilis*, was erected on the basis of fragmentary material including cranial elements, from the late Miocene of Samos (Greece). We re-examined the type material of *Varanus marathonensis* Weithofer, 1888, based on material from the late Miocene of Pikermi (Greece), and concluded that it is a valid, diagnosable species. Previously unpublished Iberian material from the Aragonian (middle Miocene) of Abocador de Can Mata (Vallès-Penedès Basin, Barcelona) and the Vallesian (late Miocene) of Batallones (Madrid Basin) is clearly referable to the same species on a morphological basis, further enabling to provide an emended diagnosis for this species. *Varanus amnhophilis* appears to be a junior subjective synonym of *V*. *marathonensis*. On the basis of the most complete fossil *Varanus* skeleton ever described, it has been possible to further resolve the internal phylogeny of this genus by cladistically analyzing 80 taxa coded for 495 morphological and 5729 molecular characters. *Varanus marathonensis* was a large-sized species distributed at relatively low latitudes in both southwestern and southeastern Europe from at least MN7+8 to MN12. Our cladistic analysis nests *V*. *marathonensis* into an eastern clade of *Varanus* instead of the African clade comprising *Varanus griseus*, to which it had been related in the past. At least two different *Varanus* lineages were present in Europe during the Neogene, represented by *Varanus mokrensis* (early Miocene) and *V*. *marathonensis* (middle to late Miocene), respectively.

## Introduction

Monitor lizards have a wide current distribution embracing Africa, Asia, and Australia [1]. The current fossil record indicates that the genus *Varanus* (Varanidae) originated as early as the late Eocene [2] and that it inhabited Europe at least from the early Miocene (MN4) to the Pleistocene [3–6]. The European fossil record is limited to about 40 localities, which have provided mostly isolated vertebrae. Such remains are however poorly diagnostic, therefore being generally identified to the genus rank only [5,7–9]. Nevertheless, a few species have been erected on the basis of European material: *Varanus amnhophilis* Conrad et al., 2012 (late Miocene; Greece), *Varanus atticus* Nopcsa, 1908 (late Miocene; Greece); *Varanus deserticolus* Bolkay, 1913 (Pliocene, Hungary); *Varanus hofmanni* Roger, 1898 (middle to late Miocene; Austria, France, Germany, Hungary, Spain, Moldova); *Varanus lungui* Zerova & Chkhikvadze, 1986 (middle Miocene, Moldova); *Varanus marathonensis* Weithofer, 1888 (late Miocene to Pliocene; Greece, Hungary); *Varanus mokrensis* Ivanov et al., 2018 (early Miocene; Czech Republic); *Varanus semjonovi* Zerova & Chkhikvadze, 1986 (late Miocene, Ukraine); and *Varanus tyrasiensis* Lungu et al., 1983 (middle Miocene, Moldova). Except for *V*. *amnhophilis*, *V*. *marathonensis* and *V*. *mokrensis*, all the other nominal species are represented by isolated vertebrae only, or by material which is not clearly referable to *Varanus*.

Recent analyses of the phylogenetic relationships of extant and extinct *Varanus*, based on a combined data set of morphologic and molecular data [5,10,11], did not take into account any of the above-mentioned European fossil taxa erected on the basis of isolated (and often fragmentary) vertebrae, which are not informative enough as to allow a thorough phylogenetic analysis. In fact, the identification to species rank of isolated varanid vertebrae is mostly hindered by the poor knowledge of the intra- and interspecific variability, and by their relatively uniform morphology, so that Holmes et al. (p. 1107 in [2]) remarked that it “is uncertain to what degree *Varanus* vertebrae are diagnostic to species”. Conrad et al. [10] listed several vertebral characters that could be useful for deciphering the phylogenetic relationships among *Varanus* species, but such characters just identify species groups, not species. An example is provided by the only unambiguous morphologic synapomorphy that diagnoses a subclade of *Varanus*: the “presence of strong precondylar constriction of the presacral vertebrae such that a right or acute angle is formed between the condyle and the centrum just anterior to the posterolateral part of the condyle (state 2 of character 233)” (p. 38 in [10]).

Conrad et al. [12] stated that, because of the scarce informativeness of isolated vertebrae and other non-diagnostic materials, all the fossil species of *Varanus* erected based on European materials are not diagnosable (this statement predates the description of *V*. *mokrensis*). Based on this assumption, Conrad et al. [11] described a new species, *V*. *amnhophilis*, from the late Miocene locality Q1 of Samos (Mytilinii Formation, Greece; MN 12, GPTS age: ~7.1 Ma [13]), which is based on fragmentary cranial and postcranial remains. According to Conrad et al. [11], such material represents the earliest evidence for a giant limbed non-marine squamate, that is to say, of a limbed squamate longer than 99% of the known fossil and living lizards.

The erection of the new species *V*. *amnhophilis* overlooked that *V*. *marathonensis*, from the late Miocene (MN12) of Pikermi (Greece; [14]) was established by Weithofer [15] based on cranial remains that were originally described with sufficient detail to provide diagnostic characters and that are still available in the collections of the Institut für Paläontologie der Universität Wien (Vienna, Austria).

In order to provide a solid ground for the study of the European monitor lizards, here we re-evaluate the morphology of the type material of *V*. *marathonensis* from Pikermi and describe new remains of the same species from two different sedimentary basins: the Vallès-Penedès Basin (northeastern Spain) and the Madrid Basin (central Spain). As briefly anticipated by Delfino et al. [16,17], we show on the basis of these materials, which include the most complete fossil *Varanus* skeleton ever described, that the species erected by Weithofer [15] is (1) diagnosable thanks to several morphological characters, (2) widespread both in eastern and western Europe, and (3) a senior synonym of *V*. *amnhophilis*. Furthermore, the phylogenetic relationships of this species with other fossil and extant *Varanus* are evaluated based on a cladistic analysis including both morphologic and molecular data, and the paleobiogeographic history of Neogene European monitor lizards is discussed.

## Material and methods

### Studied material

The studied material includes varanid remains coming from the Miocene of Greece and the Iberian Peninsula. The Greek material is stored in the Institut für Paläontologie der Universität Wien, in Vienna, whereas the Iberian material is stored in the collections of the Institut Català de Paleontologia Miquel Crusafont in Barcelona (remains from the Vallès-Penedès Basin) and of the Museo Nacional de Ciencias Naturales in Madrid (remains from Cerro de los Batallones). Further material from Greece that is stored in the National History Museum of Aegean, Mytiliniii, Samos Island, is mentioned in the discussion, even though it is not studied in detail in this paper. More than 200 skeletons of extant *Varanus* species have been studied as comparative material. A complete list of these specimens is given in S1 File. Morphological character scoring for the phylogenetic analysis is based on these specimens, as well as on literature (see S2 File). All the fossils and modern specimens studied in this project were already part of museum collections. None of them was specifically collected in the field with the goal of producing this manuscript. Therefore, no specific permits were required for the described study.

### Phylogenetic analysis methodology

We used the matrix of Conrad et al. [11] to perform a phylogenetic analysis using parsimony in order to infer the phylogenetic position of the taxon from Batallones. However, it has to be noted that the published version of the matrix (Dataset S1 of Conrad et al. [11]) had some formatting mistakes that prevented its proper working. The Editor of Conrad et al. [11] was contacted, and he kindly supplied a new, working version of the matrix. Although a link to a new version of the matrix was posted in the publication webpage comments, the link was not working the last time we tried to access it, on December 2017. Besides minor changes in the names of taxa (not affecting codings) and incongruences with the number of taxa and characters (the text reads that the matrix has 83 taxa and 6226 characters, when it actually has 80 taxa and 6223 characters), we found one problem with codings of the new matrix. The first 493 characters are morphological, and the remaining characters (5729 as stated in Conrad et al. [11]) should be the molecular ones. However, the sum of morphological and molecular characters in this matrix is 6223 (instead of 6222), suggesting that there is an extra character in the provided one. After checking the last character, we realized that this supposed molecular character is coded for fossil taxa, what suggests that it was possibly added as a new morphological character that was finally never used (and the authors would have forgotten to delete). For this reason, we treated this last character as inactive for all analyses. Following Conrad et al. [11], we considered characters 386, 389 and 402 as ordered, and we deactivated characters 236, 242, 326 and 364. The analyses were run in TNT v. 1.5 [18] using the New Technology Search option set to 1000 replicates, and with Ratchet and Drift options activated. However, we were not able to replicate the same exact results as Conrad et al. [11]. We recovered a lower number of MPTs (3960), which are slightly longer (10631 vs. 10325 steps). The topology of the Adams consensus tree is, however, almost identical, with the exception of the position of *Varanus semiremex*, which is better resolved in our analysis. The strict consensus tree presents exactly the same topology as in Conrad et al. [11]. Conrad et al. [11] did not report Nelson consensus tree, so a comparison with our results is not possible.

For our new analyses, we added the (morphological) coding for the fossil *Varanus mokrensis* as taken from Ivanov et al. [5], except for the following characters that were lacking in their analysis: 3, 7, 13, 20, 32, 39, 43, 49, 50, 52, 55, 59, 82, 83, 94, 118, 128, 141, 173, 183, 197, 236, 243, 263, 364, 374, 377, 379, 384, 393, 396, 407, 413, 414, 416, 432, 435, 448, 453, 461, 467, 469, 478, 483. We used a new coding for *Varanus marathonensis*, named “Vmarathonensis Vamnhophilis”, mainly based on the information provided by the new skeleton from Batallones, but also including the scorings for a few additional characters as provided by the type of *Varanus amnhophilis*, here considered a synonym of *V*. *marathonensis* (see below), for our main analysis (we named this one analysis 1A). The skeleton from Batallones is considered representative also for the rest of the material assigned to *V*. *marathonensis* (namely, Weithofer’s material, Gaudry’s vertebra, and the rest of the Iberian remains; see below), given that their scorings are redundant (sensu [19]) and all characters that can be scored in these remains can be scored identically for the skeleton from Batallones too. We performed a second analysis (2A) where *V*. *marathonensis* was scored separate from *V*. *amnhophilis*. For this first set of analyses (1A, 2A), the last three characters of the matrix were deactivated. These three characters corresponded to the last one in the matrix provided by Conrad (considered a character added in error, see above), and two new characters added herein. A variation of the same analyses was performed in order to test the influence of the addition of the new characters. The second set of analyses (1B, 2B) comprises the same as above, but with the last two morphological characters of the matrix (added in the present work) activated (see S4 File).

### Anatomical abbreviations

The anatomical terminology follows the one used in Conrad’s works [10,11]. Anatomical abbreviations are as follows: ac, articular condyle; af, adductor fossa; alf, alveolar foramina; alp, anterolateral process; anf, antorbital flange; anp, anterior process; ap, alar process; art, articular; asf, anterior surangular foramen; bo, basioccipital; bp, basal plate; bpp, basipterygoid process; cb, chevron bone; cbl, coracoid blade; cem, coronoid eminence; cf, coracoid foramen; cjf, crista juxtafovealis; con, condyle; cot, cotyle; cpf, crista postfovealis; crc, crista cranii; crl, crista lateralis; crt, crista transversalis; cv, caudal vertebra; d, diapophysis; de, distal epiphysis; dop, dorsal process; dp, descensus parietalis; dpc, deltopectoral crest; ds, dorsum sellae; fap, facial process; fh, femoral head; fm, foramen magnum; fop, fossa parietalis; frp, frontal process; fsv, first sacral vertebra; gf, glenoid fossa; gs, glenoid surface; h, hypapophysis; hh, humeral head; ip, incisive process; it, internal trochanter; lai, lamina intercristalis; lar, lacrimal recess; las, lateral sulcus; lf, lacrimal foramen; maf, maxillary flange; map, maxillary process; mca, Meckel's canal; mefp, medial expansion of the facial process; mec, medial crest; mf, mental foramen; nap, nasal process; nes, neural spine; oc, occipital condyle; oto, otooccipital; pag, palatine groove; pap, palatine process; par, paranasal recess; pas, palatine shelf; pca, posterior canal; pcb, pedestal for chevron bone; pf, pineal foramen; plf, posterolateral flange; pmp, posterior maxillary process; poc, posterior crest; popo, paroccipital process of the otooccipital; popp, paroccipital process of the prootic; poz, postzygapophysis; pp, parietal process; ppr, posterior process; pra, processus ascendens; pre, prearticular; pro, prootic; prz, prezygapophysis; psf, posterior surangular foramen; psp, parasphenoid process; ptp, pterygoid process; qp, quadrate process; rap, retroarticular process; rc, radial condyle; rlf, rim of the lacrimal foramen; sar, sagittal ridge; sh, shaft; sot, sphenooccipital tubercle; sqp, squamosal process; ssv, second sacral vertebra; stp, supratemporal process; suo, supraoccipital; sup, sublacrimal process; sur, surangular; sut, subdental table; syn, synapophysis; trp, transverse process; tuc, tuberal crest; tv, trunk vertebra; tyc, tympanic crest; uc, ulnar condyle; vop, vomerine process.

## Fossil locality abbreviations

ACM, local stratigraphic series of Abocador de Can Mata (els Hostalets de Pierola, Vallès-Penedès Basin, Spain); BAT, Cerro de los Batallones (Torrejón de Velasco, Madrid Basin, Spain); C2, C3, C4, C5, different cells from ACM (the capital letter that might follow the cell, separated by a dash, denotes a subsector, whereas the number or lowercase letter that follows without a dash denotes the exact locality—stratigraphic horizon—within that sector or subsector; [20]).

### Institutional abbreviations

CM, Carnegie Museum of Natural History (Pittsburgh, USA); ICP, Institut Català de Paleontologia Miquel Crusafont, Universitat Autònoma de Barcelona (Barcelona, Spain); IPS, ‘Institut de Paleontologia de Sabadell’, acronym of the collections of the ICP; IPUW, Institut für Paläontologie der Universität Wien (Vienna, Austria); MNCN, Museo Nacional de Ciencias Naturales–CSIC (Madrid, Spain); SMF, Senckenberg Museum Frankfurt (Germany); ZFMK, Zoologisches Forschungsmuseum Alexander Koenig (Bonn, Germany).

## Results

### Age and geological setting of the localities

#### Vallès-penedès localities

The Vallès-Penedès Basin (Catalonia, Spain) is a half-graben that originated as a result of the extensional tectonics that started by the earliest Neogene in the Western Mediterranean, due to the northward movement of the African plate [21–24]. The sedimentary sequences of this basin cover most of the Miocene [25], mainly corresponding to alluvial fan sediments, although some marine and transitional sequences were deposited during the early and middle Miocene. Sedimentation continued throughout the middle and late Miocene, resulting in a considerable sediment thickness due to continuous subsidence, particularly near to the main active fault, located in the NW margin of the basin.

The Vallès-Penedès Basin has delivered a rich fossil record of terrestrial vertebrates from the early to the late Miocene [26,27]. The ACM series [20,28–30] is situated in the area of els Hostalets de Pierola, which is characterized by thick middle to late Miocene alluvial sequences that correspond to the Upper Continental Complex of the basin [25,31]. The middle Miocene sequences of els Hostalets mainly consist in red to brown mudstones, sandstones, breccias and conglomerates, which were deposited in the distal-to-marginal, inter-fan zones of the coalescing alluvial fan systems of els Hostalets de Pierola and Olesa [20,29,32]. Such depositional environment would have favored the preservation of vertebrate remains in a mudstone-dominated sedimentary environment, thanks to the combination of high rates of subsidence and sediment supply [32]. The exact stratigraphic position of classical localities from els Hostalets [33,34] is unknown in many instances, having traditionally been grouped into Hostalets Inferior (Aragonian levels) and Hostalets Superior (Vallesian levels) [31,33–35]. This contrasts with the situation for the 250 m-thick ACM series, where more than 200 fossil vertebrate localities have been formally defined [20]. The age of these localities and many isolated finds can be accurately estimated on the basis of firm litho-, bio- and magnetostratigraphic correlation (for the last update, see Table S2 in [20]). The whole ACM series corresponds to the late Aragonian (middle Miocene), and from a biostratigraphic viewpoint it can be correlated to the late portion of MN6 and most the MN7+8 [20,36]). On the basis of magnetostratigraphic correlation [20,32,37]), the ACM series spans from ca. 12.6 to 11.4 Ma, and estimated interpolated ages for the several localities can be provided on the basis of average local sedimentation rates for each subchron [20]. The material described here comes from ACM localities C2-A3, C3-A6, C3-A7 and C4-C2. C3-A7 and C2-A3 are the oldest localities (the former being stratigraphically 5 m below the latter), with an interpolated estimated age of 12.0 Ma within C5r.3r; C3-A6 and C4-C2 are slightly younger, being stratigraphically situated several meters above C2-A3 (10 and 20 m, respectively), with an interpolated estimated age of 11.9 Ma, also within C5r.3r. All these localities are correlated to the *Megacricetodon crusafonti* + *Democricetodon crusafonti* Interval Subzone of the Vallès-Penedès Basin [20,36]), representing the earlier portion of MN7+8.

#### Cerro de los Batallones

Cerro de los Batallones (Torrejón de Velasco, Madrid Basin) includes a set (BAT-1 to BAT-10) of exceptionally rich paleontological sites of Vallesian age (ca. 10–9 Ma, MN10, late Miocene; [38–41]. These sites are situated on the top of the Intermediate Miocene Unit of the Madrid Basin, and are probably synchronous with or even posterior to the deposition of the upper Miocene Unit of the above-mentioned basin. BAT-1 was discovered during the industrial exploitation of sepiolite deposits [38,40,42], which are interpreted as a result of neoformative and transformational processes of fine sediments in a lacustrine margin, due to periodical episodes of lowering of the water level and subaerial exposition [40]. Sedimentological evidence suggests that sepiolite deposits were formed in a lacustrine-palustrine depositional context, whereas fossil remains were accumulated in holes, formed due to pseudokarstic processes (piping), that acted as natural traps [39–41,43] (see [44] for further details on the taphonomy of Batallones). The *Varanus* material described in this paper comes from BAT-3, which on the basis of micromammalian remains [45] is probably somewhat younger than BAT-1, although being also correlated to MN10.

### Systematic paleontology

Order Squamata Oppel, 1811
Superfamily Varanoidea Camp, 1923
Family Varanidae Gray, 1827
Genus *Varanus* Merrem, 1820
*Varanus marathonensis* Weithofer, 1888
(Figs 1–15)

**Fig 1:**
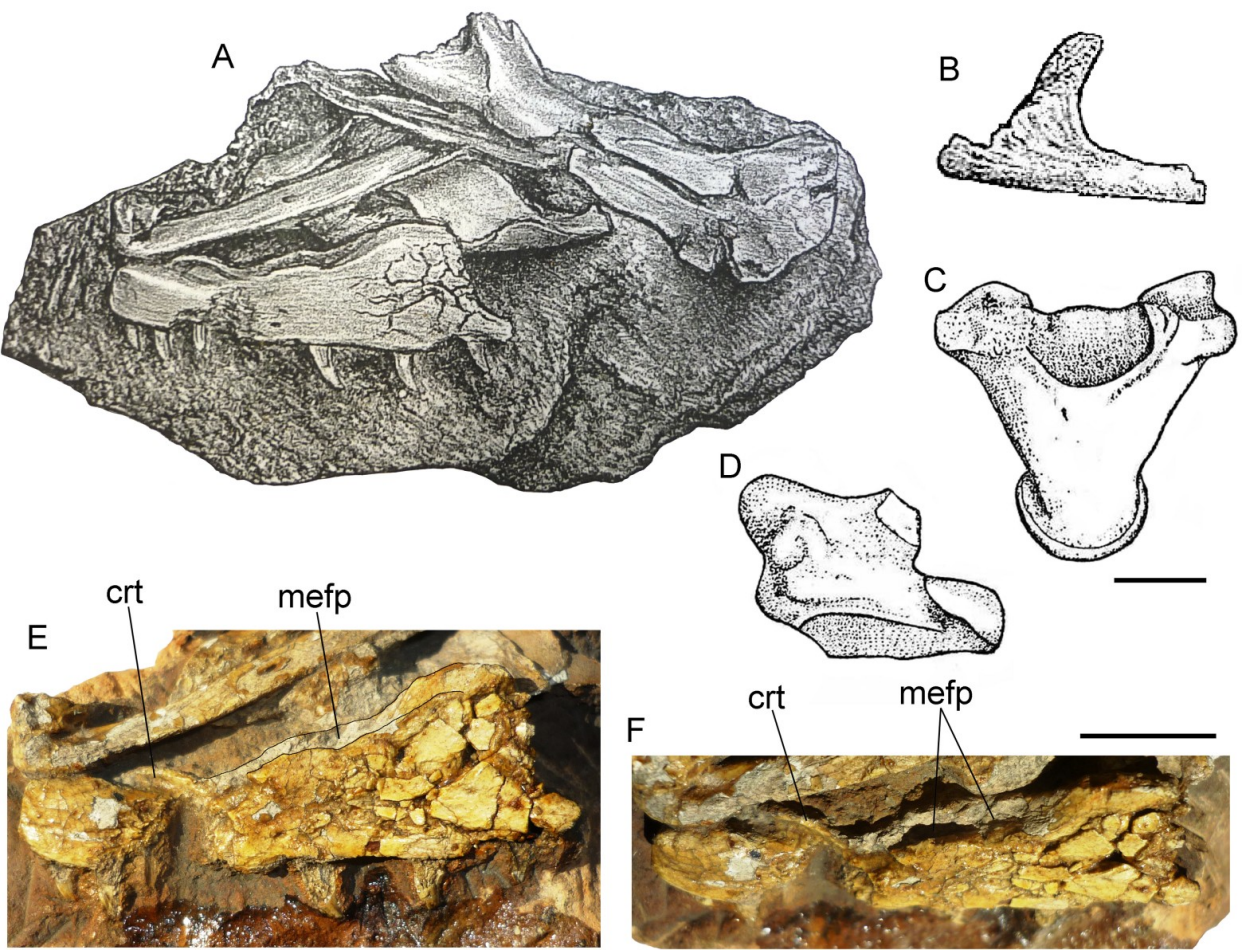
Historical material of *Varanus marathonensis* Weithofer, 1888. (A) Lectotype IPUW 1888-001-001, left maxilla from the late Miocene, MN12, of Pikermi (Greece) as figured in its original description [15]. (B) Paralectotype, isolated supraorbital from Pikermi (currently lost). (C, D) Ventral and left lateral view of the vertebra MNHN Pikermi n. 31 from the same locality as illustrated by Gaudry [49]. (E) Lateral view of the lectotype IPUW 1888-001-001 as is preserved nowadays. (F) Dorsolateral view of the same maxilla. Note that the expanded surface on the maxilla (mefp) was already shown by the original drawing by Weithofer, who also described it in the accompanying text. Scale bars equal 10 mm; (A) and (B) not to scale.

**Fig 2:**
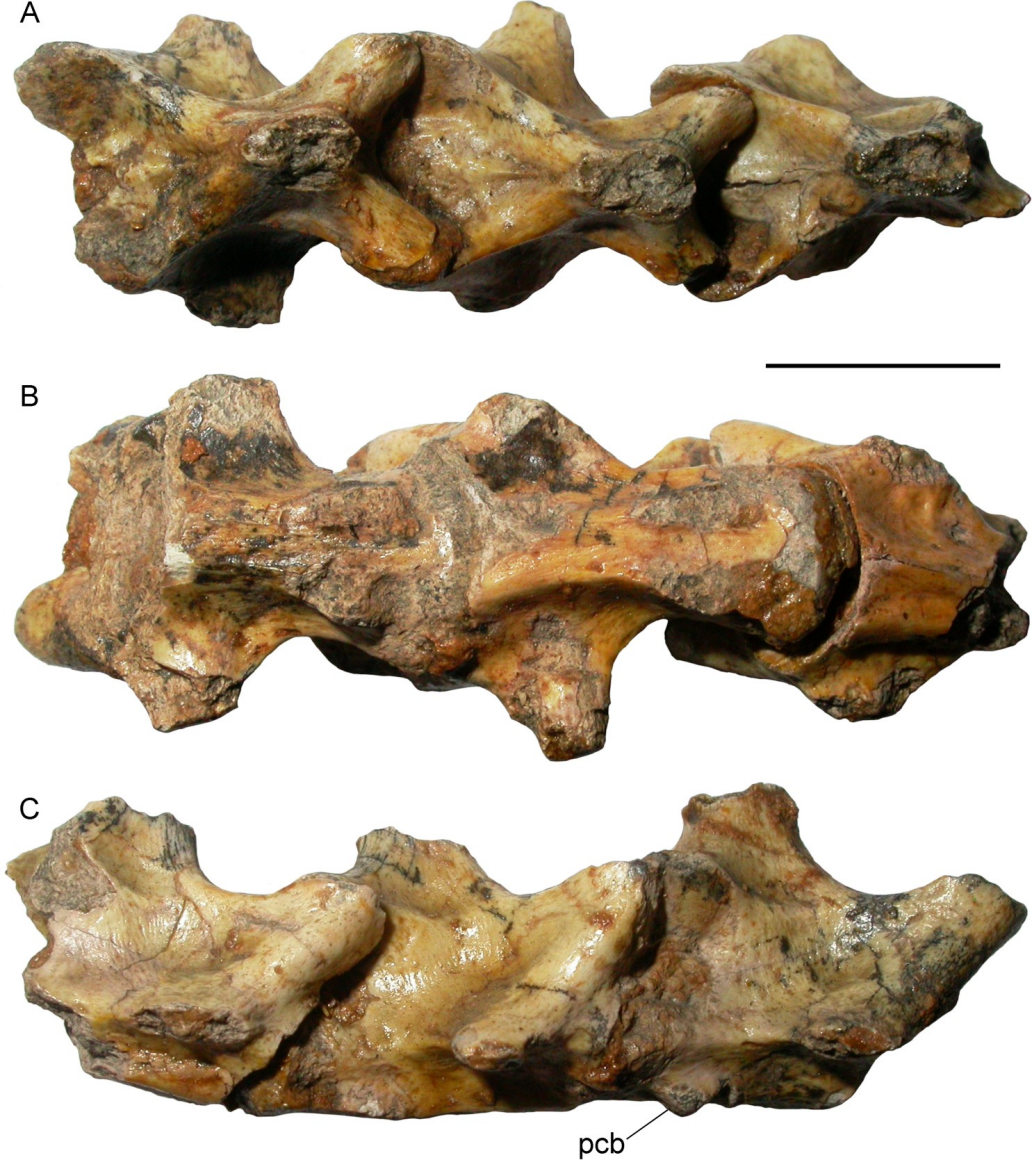
Topotypic vertebrae of *Varanus marathonensis* Weithofer, 1888. (A–C) Caudal vertebrae, IPUW 5187, from Pikermi (Greece), in dorsal (A), ventral (B) and right lateral (C) views. Note the pedestal for the chevron bone located close to the condyle (but separated from it). Scale bar equals 10 mm.

**Fig 3:**
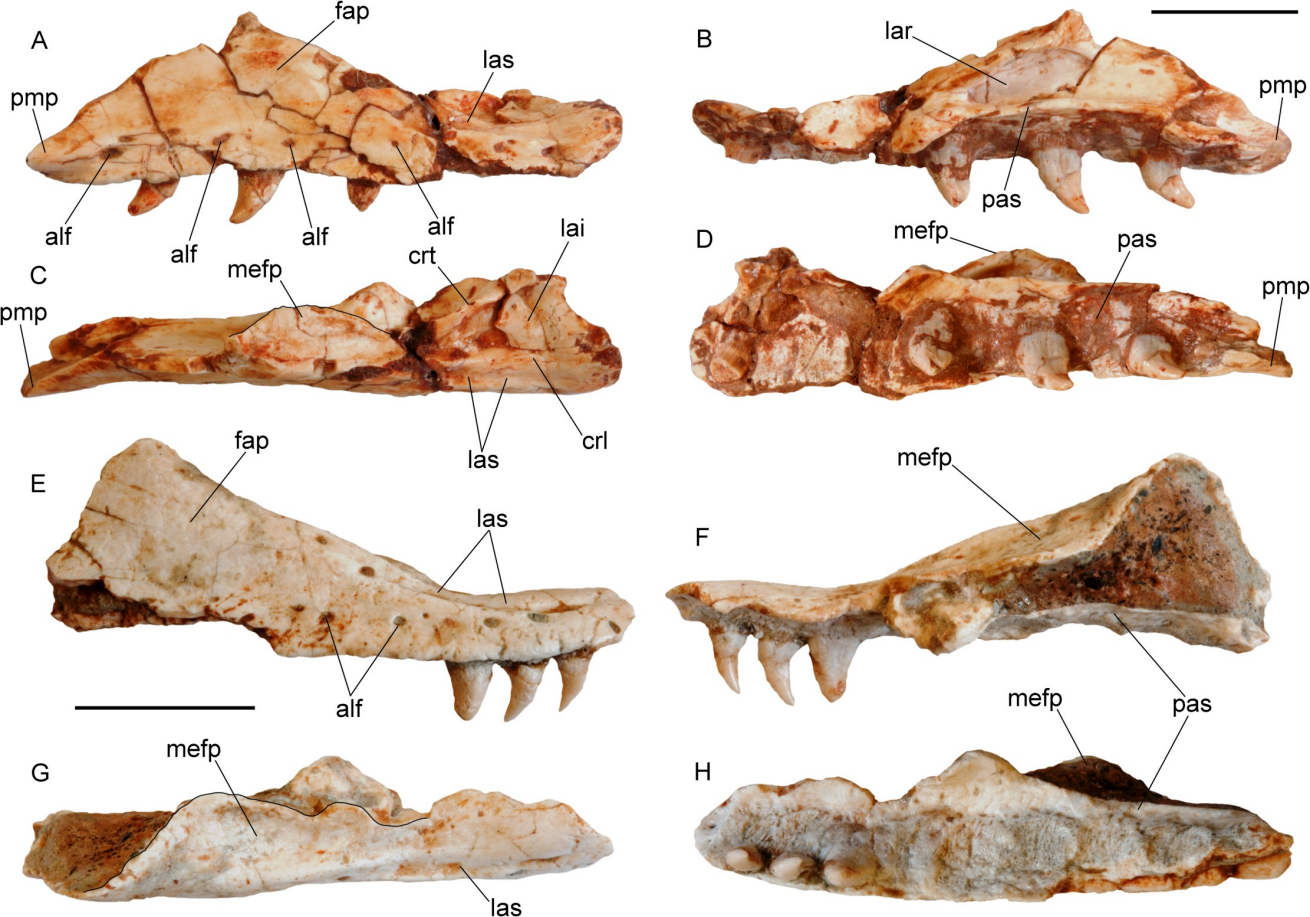
Maxillae of *Varanus marathonensis* Weithofer, 1888 from Abocador de Can Mata. (A–D) Right maxilla IPS50119 from the middle Miocene, MN7+8, of ACM/C4-C2 in lateral (A), medial (B), dorsal (C), and ventral (D) views. (E–H) Same as above but right maxilla IPS50292 from ACM/C3-A6. Note the medially expanded ascending surface of the facial process and the evident lateral sulcus developed along the narial margin. Scale bars equal 10 mm.

**Fig 4:**
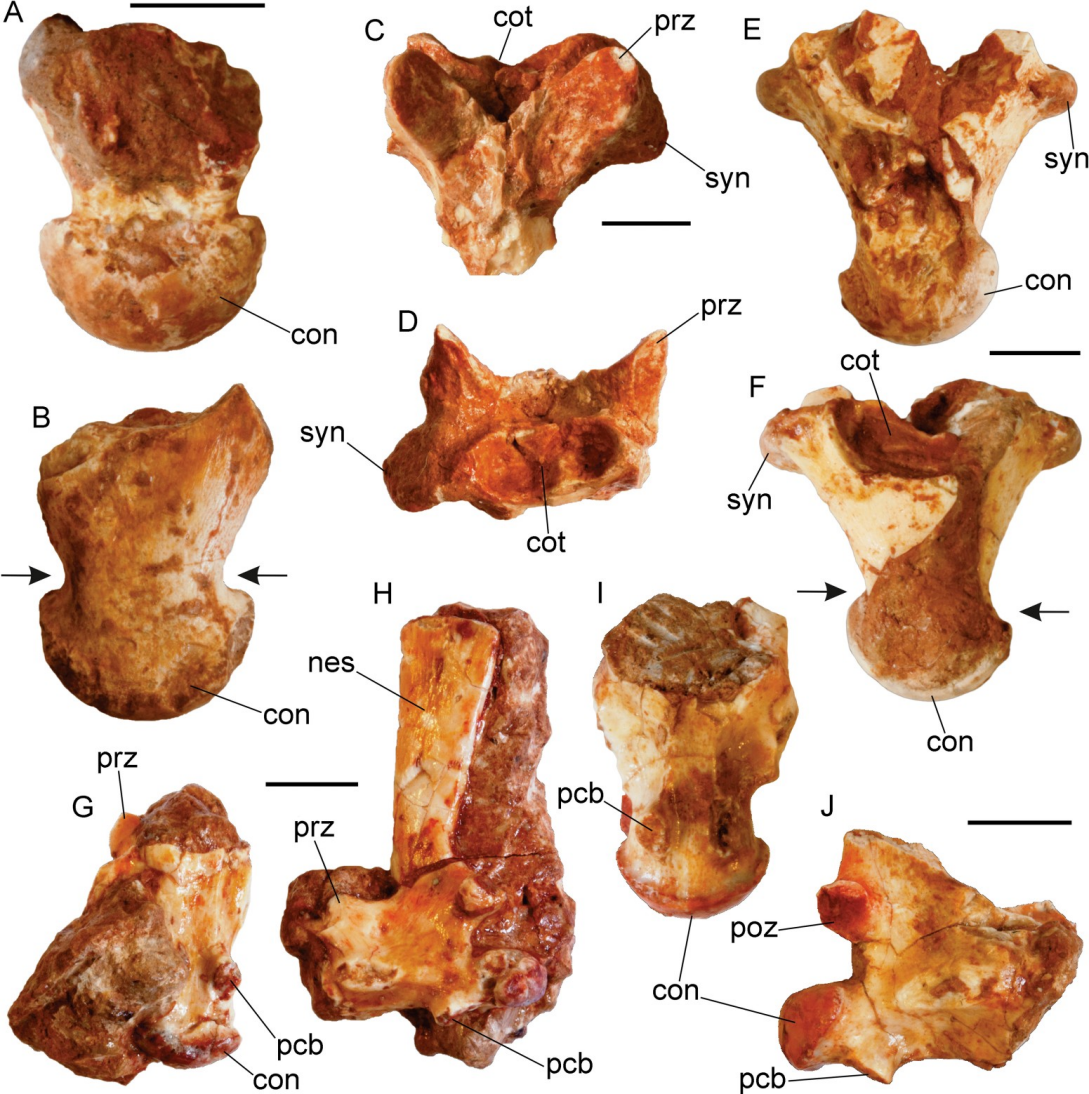
Vertebrae of *Varanus marathonensis* Weithofer, 1888 from Abocador de Can Mata. (A, B) Fragmentary trunk vertebra IPS42353 from ACM/C4-C2 in dorsal (A) and ventral (B) views. (C, D) Fragmentary trunk vertebra IPS42362 from ACM/C4-C2 in dorsal (C) and anterior (D) views. (E, F) Fragmentary trunk vertebra IPS43719 from ACM/C4-C2 in dorsal (D) and ventral (F) views. (G, H) Caudal vertebra IPS42400 from ACM/C4-C2 in ventral (G) and left lateral (H) views. (I, J) Caudal vertebra IPS42368 from ACM/C4-C2 in ventral (I) and right lateral (J) views. The arrows indicate the precondylar constriction. Scale bars equal 5 mm.

**Fig 5:**
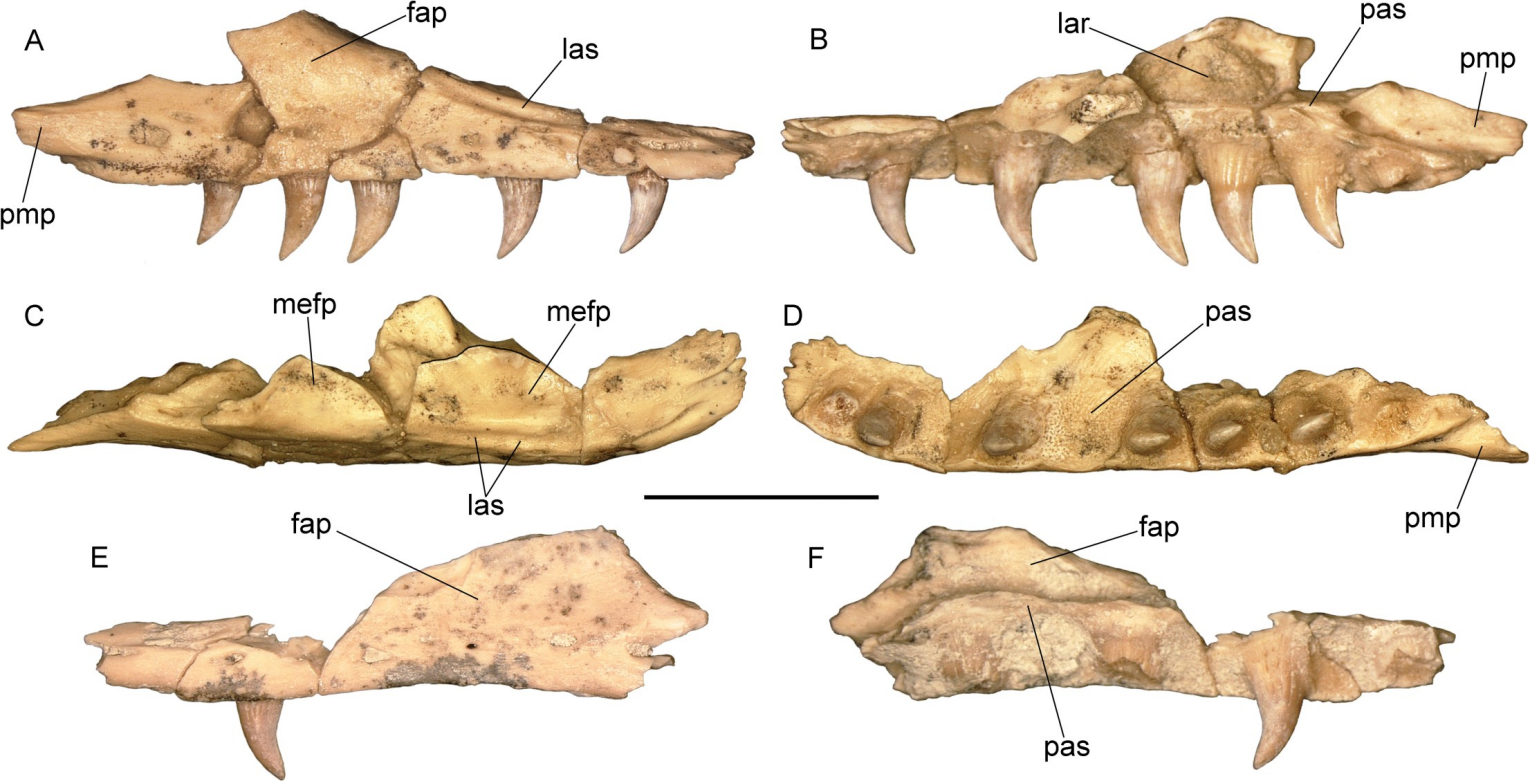
Isolated maxillae of *Varanus marathonensis* Weithofer, 1888 from Cerro de los Batallones. (A–D) Right maxilla BAT-3 1088a from the late Miocene, MN10, of Cerro de los Batallones in lateral (A), medial (B), dorsal (C), and ventral (D) views. (E, F) Left maxilla BAT-3 1088b in lateral (E) and medial (F) views. Scale bar equals 10 mm.

**Fig 6:**
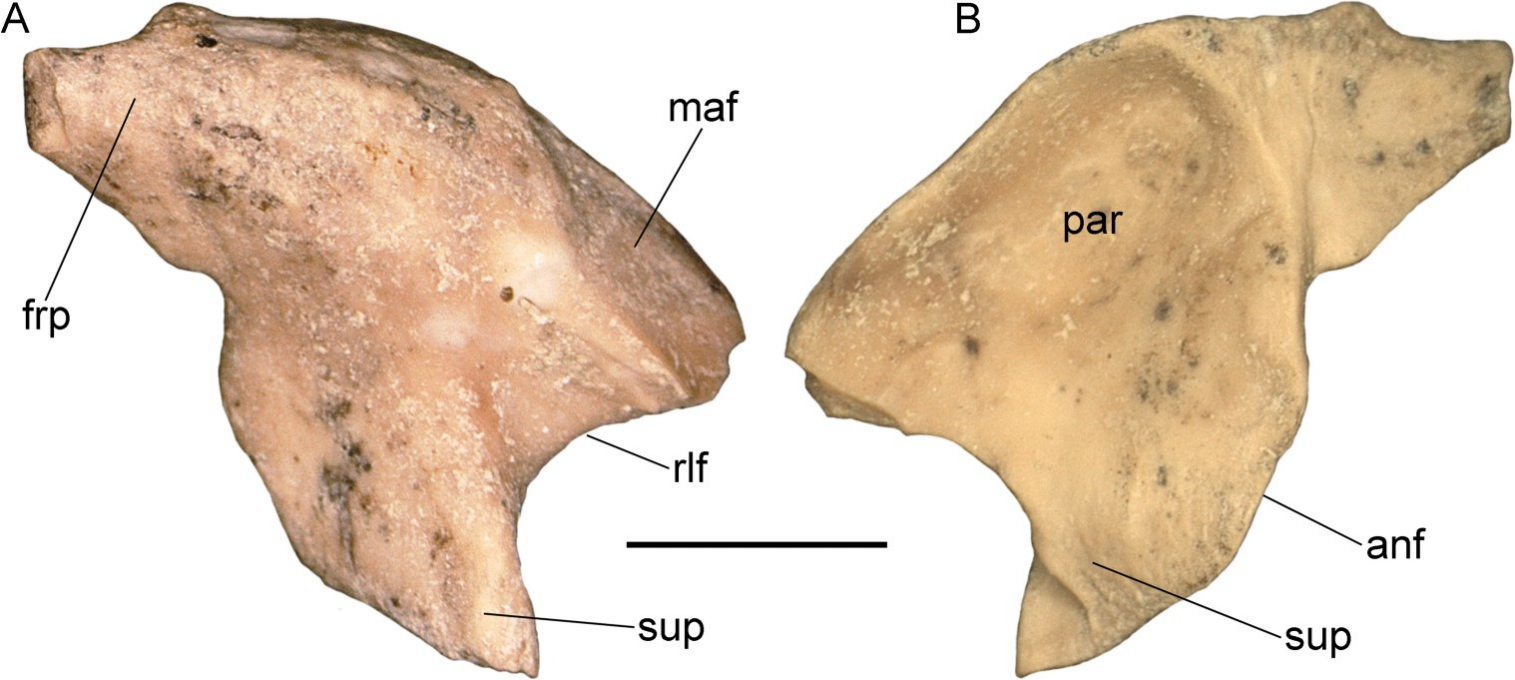
Isolated prefrontal of *Varanus marathonensis* Weithofer, 1888 from Cerro de los Batallones. (A, B) Right prefrontal BAT-3 1088c from Cerro de los Batallones in external (A) and internal (B) views. Scale bar equals 5 mm.

**Fig 7:**
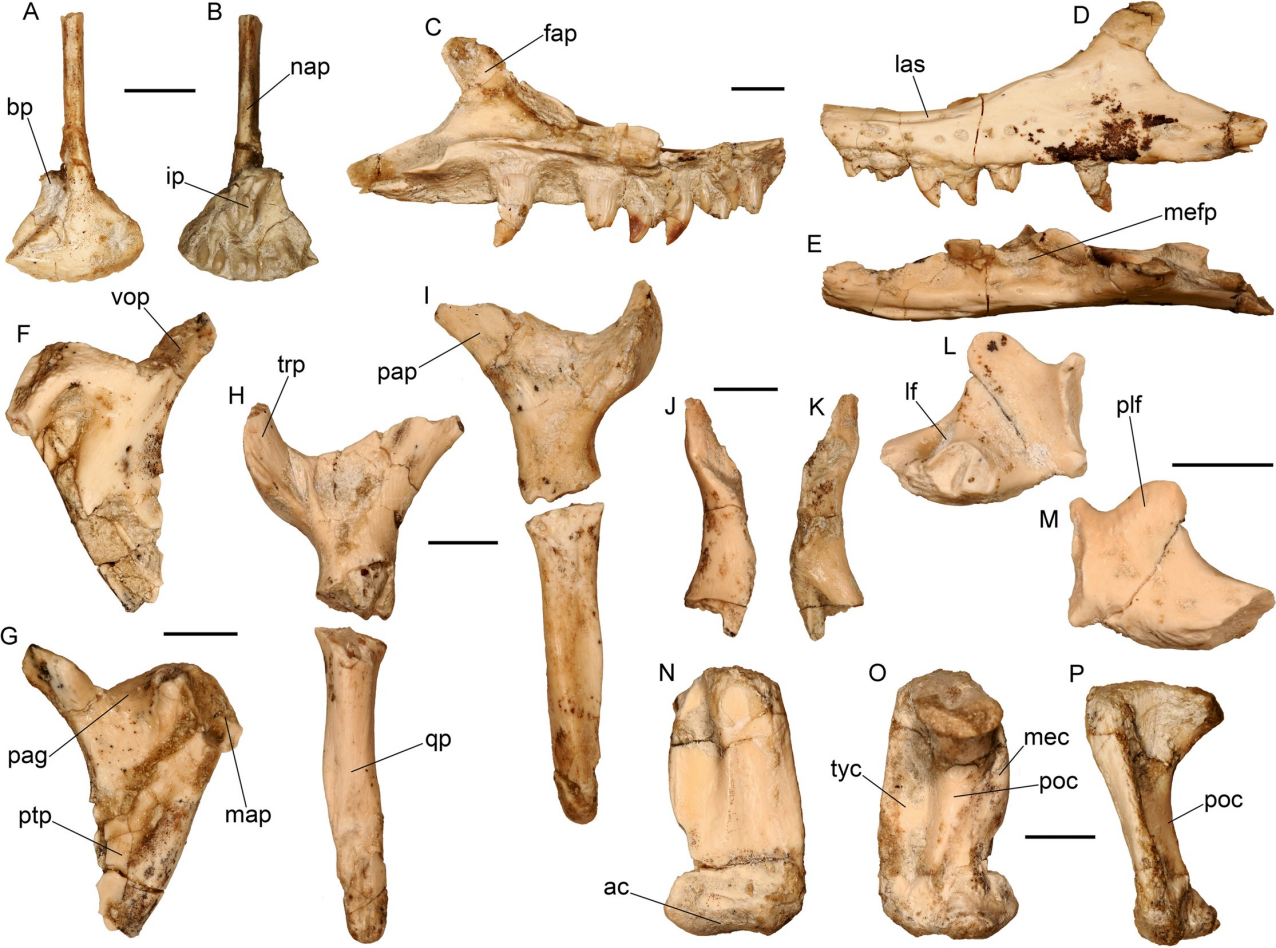
Cranial bones of the almost complete skeleton of *Varanus marathonensis* Weithofer, 1888 from Batallones-3. (A, B) Premaxilla in anterior (A) and posterior (B) views. (C–E) Left maxilla in medial (C), lateral (D), and dorsal (E) views. (F, G) Left palatine in dorsal (F) and ventral (G) views. (H, I) Left pterygoid in dorsal (H) and ventral (I) views. (J, K) Right ectopterygoid in dorsal (J) and ventral (K) views. (L, M) Left lacrimal in medial (L) and lateral (M) views. (N–P) Left quadrate in anterior (N), posterior (O), and lateral (P) views. Scale bars equal 5 mm.

**Fig 8:**
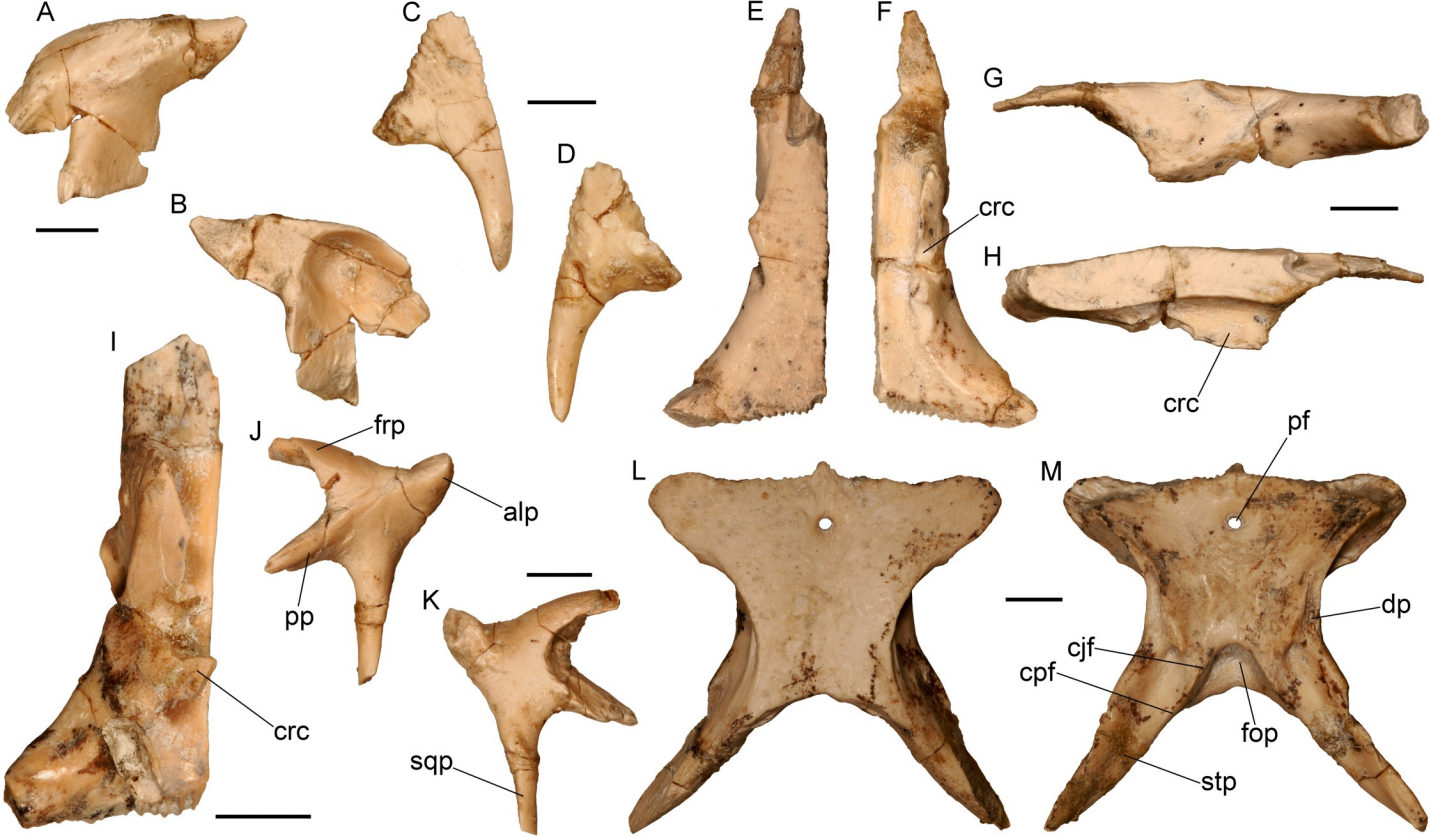
Cranial bones of the almost complete skeleton of *Varanus marathonensis* Weithofer, 1888 from Batallones-3. (A, B) Left prefrontal in lateral (A) and medial (B) views. (C, D) Right supraorbital in dorsal (C) and ventral (D) views. (E–H) Left frontal in dorsal (E), ventral (F), lateral (G), and medial (H) views. (I) Right frontal in ventral view. (J, K) Left postorbitofrontal in ventral (J) and dorsal (K) views. (L, M) Parietal in dorsal (L) and ventral (M) views. Scale bars equal 5 mm.

**Fig 9:**
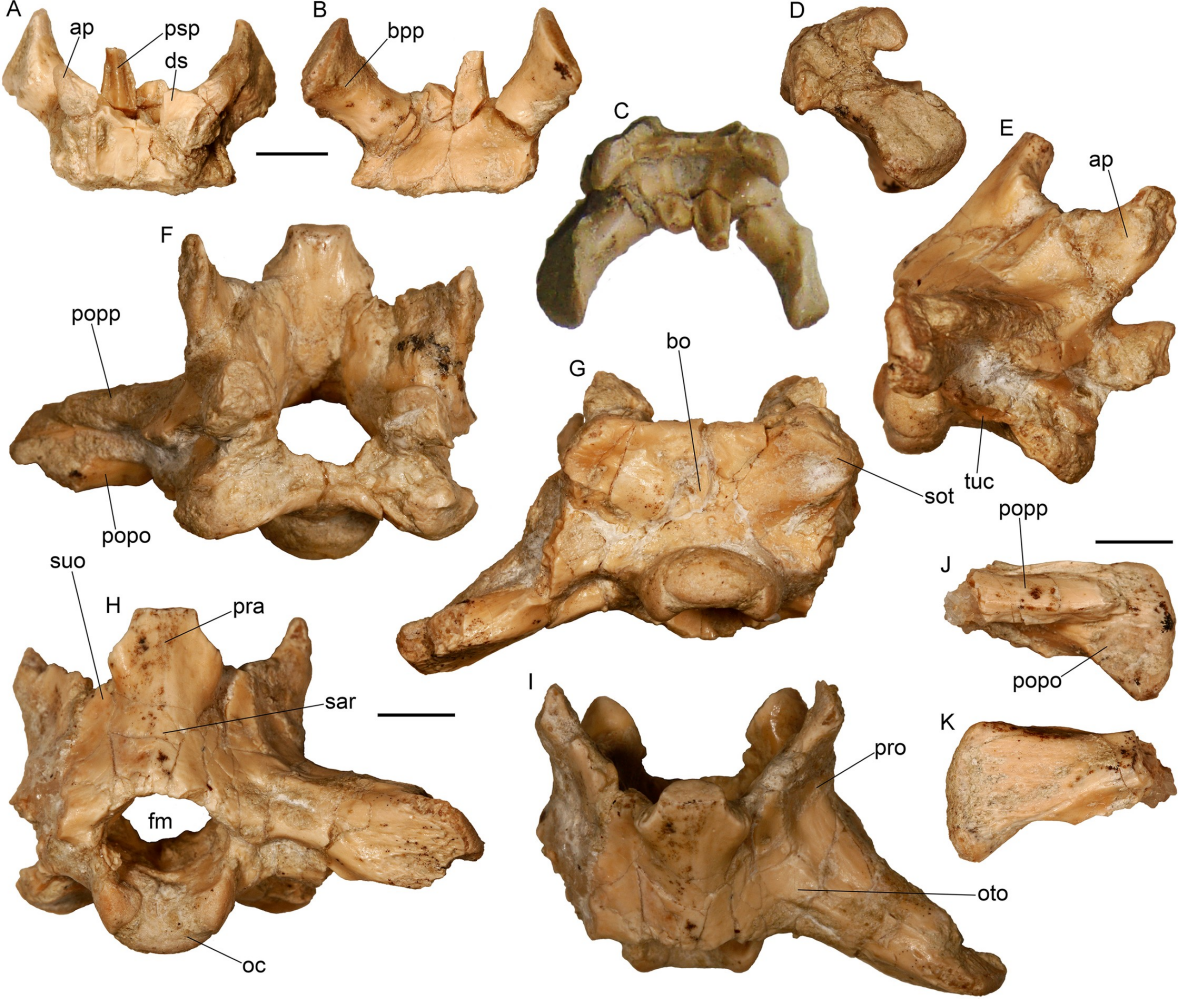
Braincase of the almost complete specimen of *Varanus marathonensis* Weithofer, 1888 from Batallones-3. (A–D) Isolated parabasisphenoid in dorsal (A), ventral (B), anterior (C), and right lateral (D) views. (E–I) Braincase in right lateral (E), anterior (F), ventral (G), posterior (H), and dorsal (I) views. (J, K) Isolated left paroccipital process in anterior (J) and posterior (K) views. Scale bars equal 5 mm.

**Fig 10:**
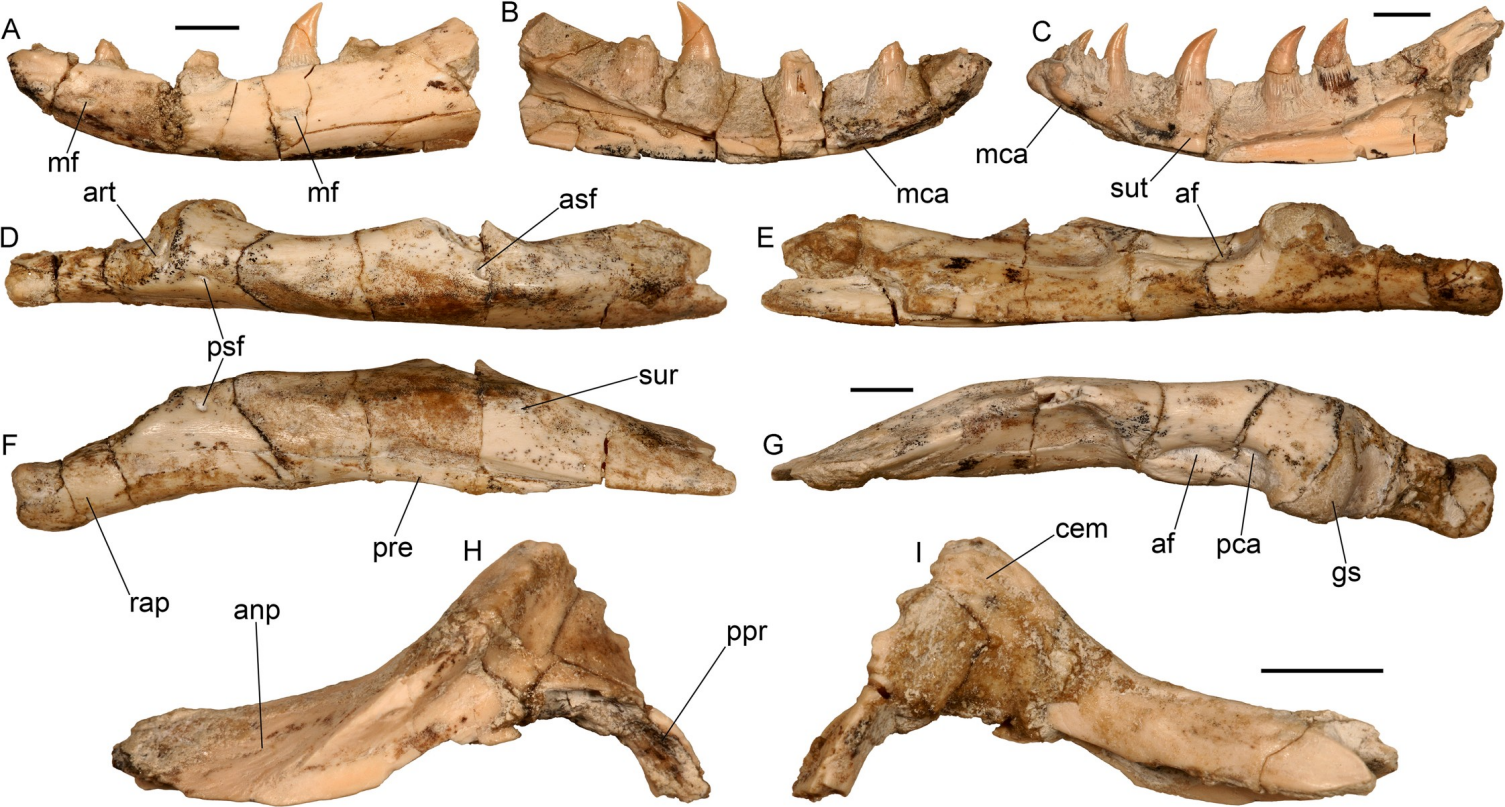
Lower jaw of the almost complete specimen of *Varanus marathonensis* Weithofer, 1888 from Batallones-3. (A, B) Left dentary in lateral (A) and medial (B) views. (C) Right dentary in medial view. (D–G) Posterior part of the right mandible (surangular, prearticular, articular) in lateral (D), medial (E), ventral (F), and dorsal (G) views. (H, I) Left coronoid in lateral (H) and medial (I) views. Scale bars equal 5 mm.

**Fig 11:**
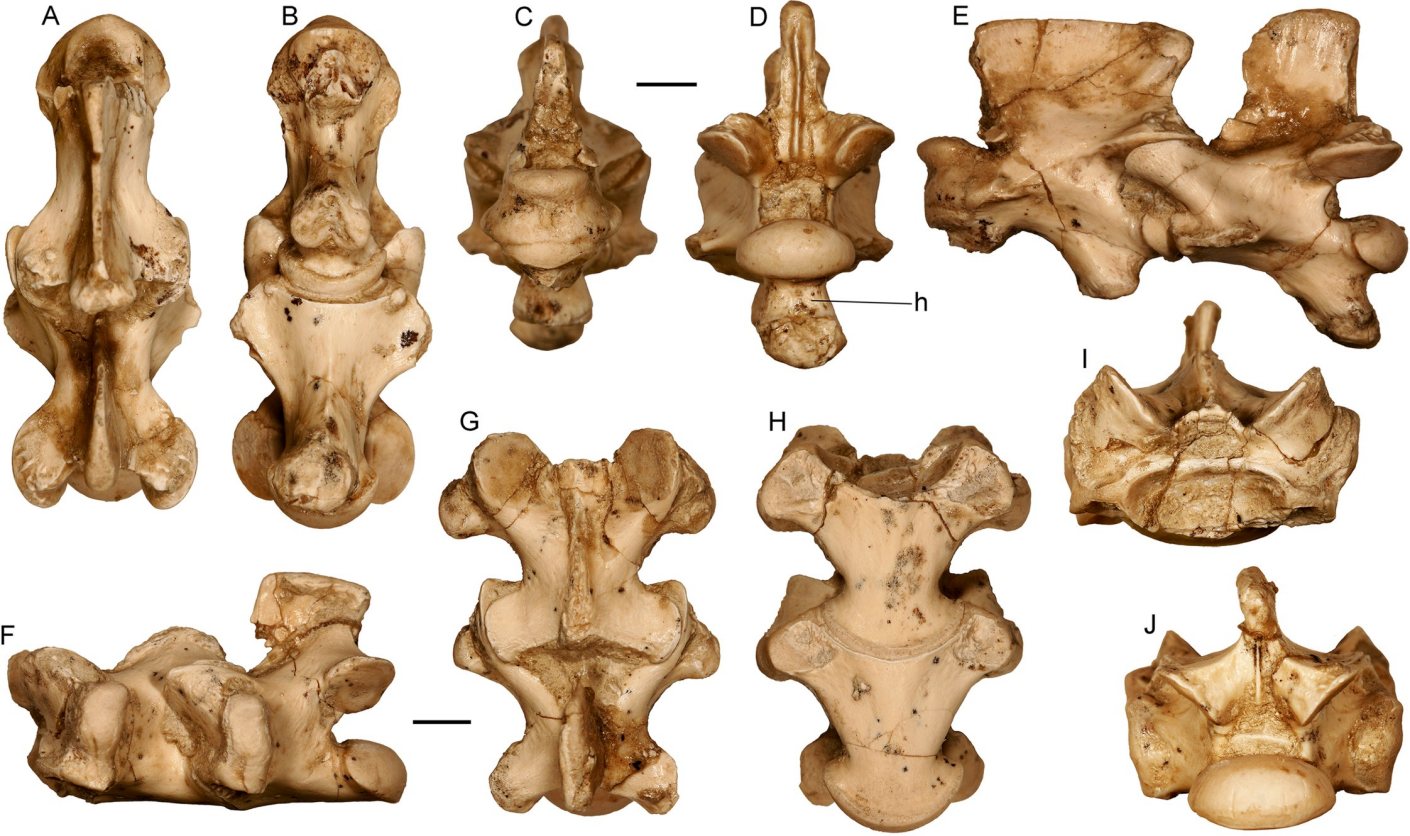
Presacral vertebrae of the almost complete specimen of *Varanus marathonensis* Weithofer, 1888 from Batallones-3. (A–E) Axis and third cervical vertebra in dorsal (A), ventral (B), anterior (C), posterior (D), and left lateral (E) views. (F–J) Trunk vertebrae in left lateral (F), dorsal (G), ventral (H), anterior (I), and posterior (J) views. Scale bars equal 5 mm.

**Fig 12:**
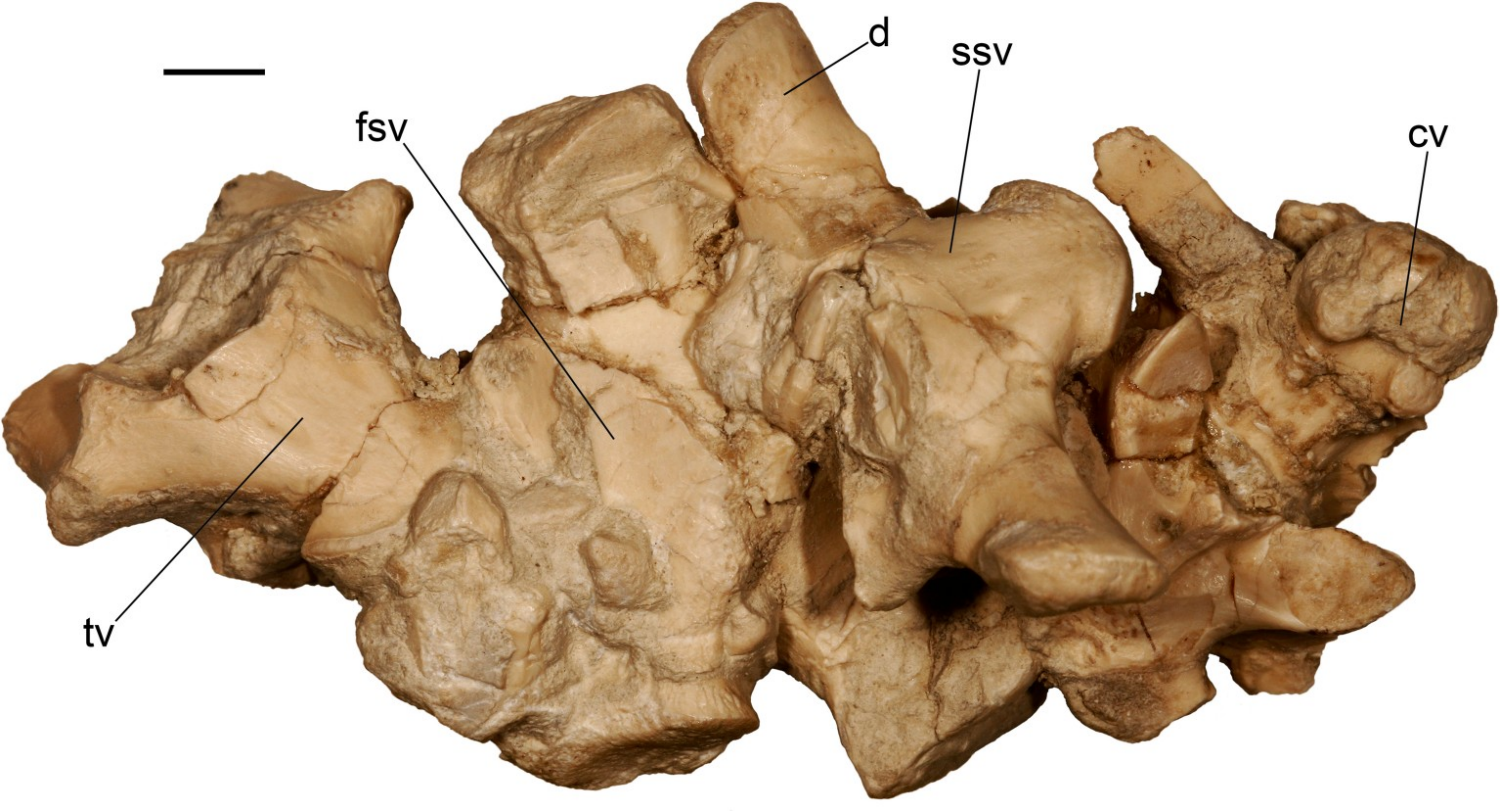
Sacral region of the almost complete specimen of *Varanus marathonensis* Weithofer, 1888 from Batallones-3, including the last trunk vertebra, two sacral vertebrae, one cloacal vertebra (plus a fragment of a possible second one), and first caudal vertebra. Scale bars equal 5 mm.

**Fig 13:**
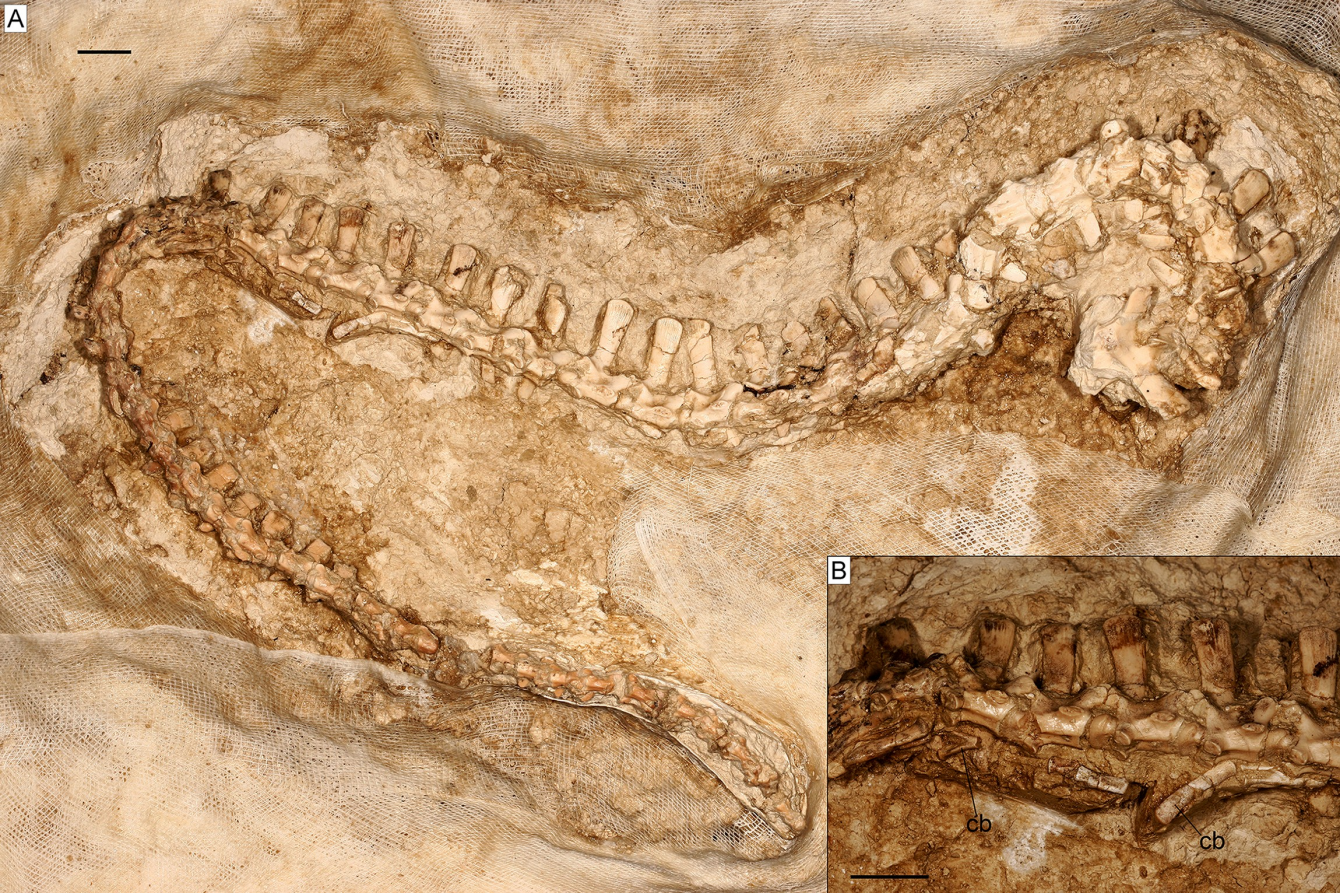
Tail of the almost complete specimen of *Varanus marathonensis* Weithofer, 1888 from Batallones-3. (A) Complete tail. (B) Detail of the caudal vertebrae in right lateral view. Scale bars equal 10 mm.

**Fig 14:**
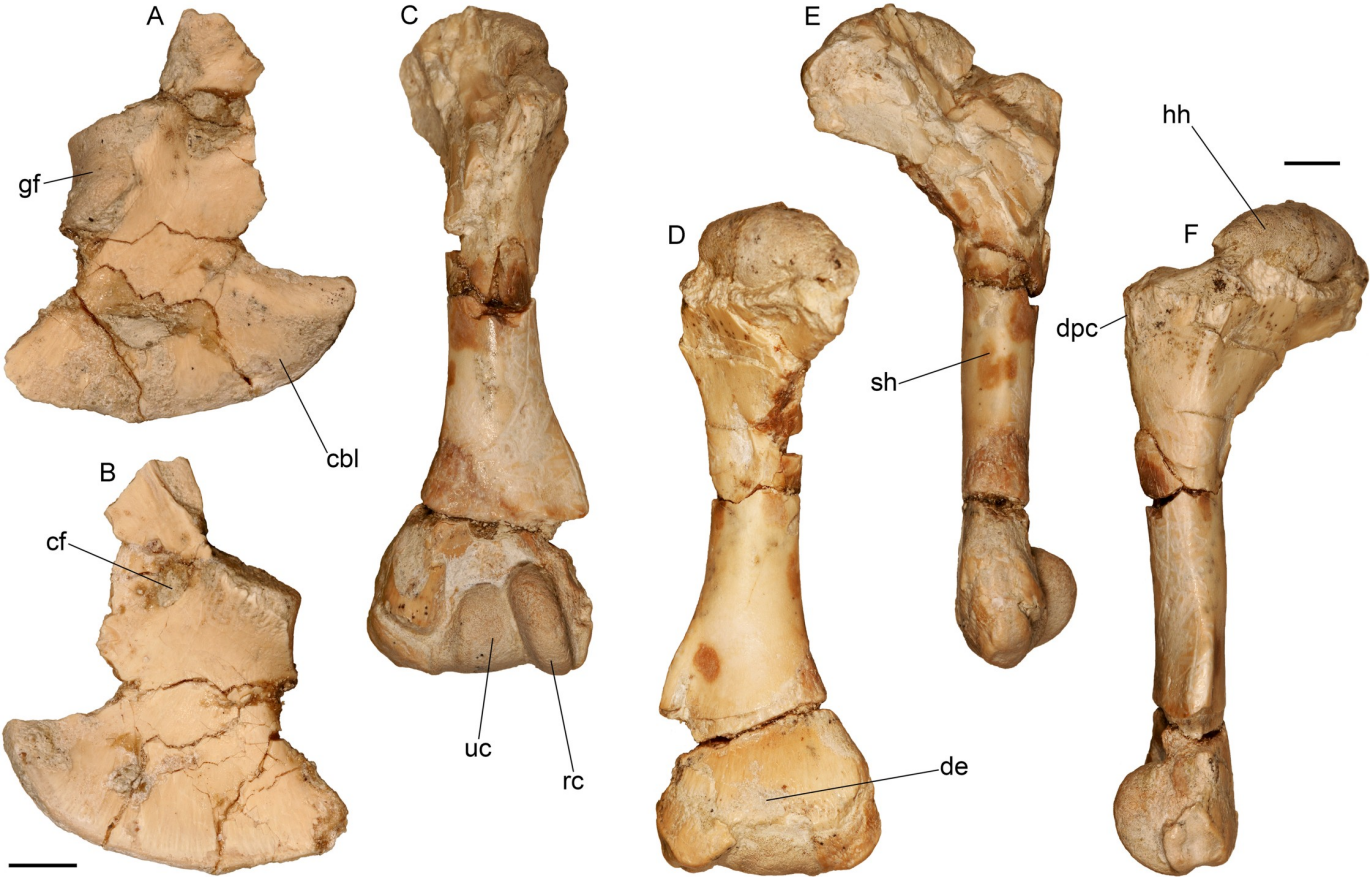
Forelimb of the almost complete specimen of *Varanus marathonensis* Weithofer, 1888 from Batallones-3. (A, B) Right coracoid in lateral (A) and medial (B) views. (C–F) Left humerus in anterior (C), posterior (D), dorsal (E), and ventral (F) views. Scale bars equal 5 mm.

**Fig 15:**
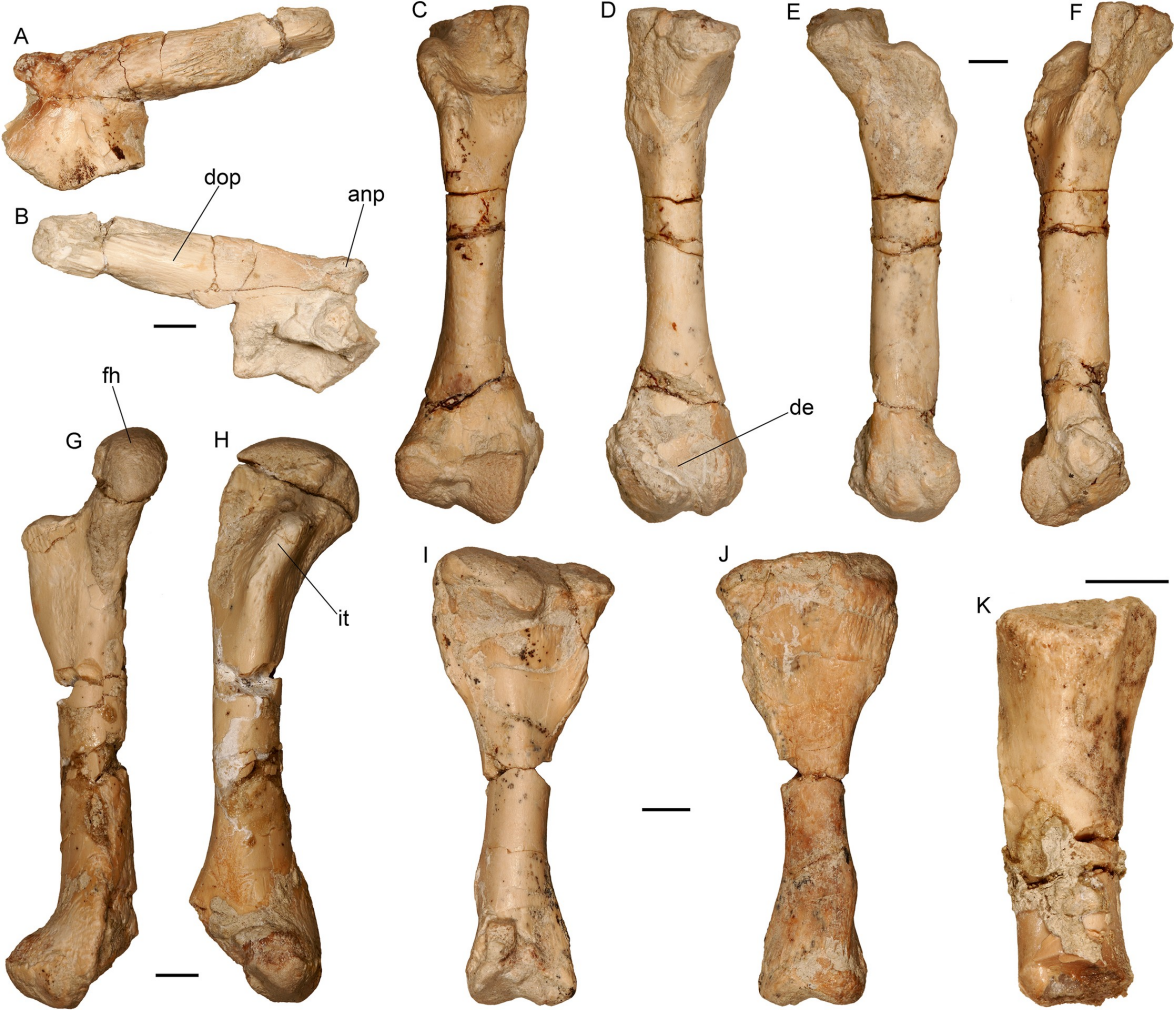
Hindlimb of the almost complete specimen of *Varanus marathonensis* Weithofer, 1888 from Batallones-3. (A, B) Left ilium in lateral (A) and medial (B) views. (C–F) Right femur in ventral (C), dorsal (D), anterior (E), and posterior (F) views. (G, H) Left femur in anterior (G) and ventral (H) views. (I, J) Left tibia in ventral (I) and dorsal (J) views. (K) Proximal end of an undetermined fibula in ventral view. Scale bars equal 5 mm.

1908 *Varanus atticus* Nopcsa: 47.
2012 *Varanus amnhophilis* Conrad, Balcarcel & Mehling: 2, figs 2, 3.

**Lectotype.** IPUW 1888-001-001, a slab preserving at least a left incomplete maxilla.

**Paralectotype.** Isolated supraorbital figured by Weithofer ([15], plate 19, fig. 9), currently lost.

**Topotype.** IPUW 5187, three incomplete caudal vertebrae joined together by sediment, plus three other highly fragmentary isolated caudal vertebrae.

**Referred specimens.** IPS50119 (from ACM/C4-C2), right maxilla; IPS50292 (from ACM/C3-A6), right maxilla; IPS57544 (from ACM/C3-A7), tooth-bearing bone; IPS42353 (from ACM/C4-C2), trunk vertebra; IPS42362 (from ACM/C4-C2), trunk vertebra; IPS43719 (from ACM/C4-C2), trunk vertebra; IPS57546 (from ACM/C2-A3), trunk vertebra; IPS42400 (from ACM/C4-C2), caudal vertebra; IPS49408 (from ACM/C2-A3), caudal vertebra; IPS42368 (from ACM/C4-C2), caudal vertebra; IPS57547 (from ACM/C2-A3), caudal vertebra; MNCN BAT-3 1088, right and left maxillae, right prefrontal; MNCN BAT-3 2011–200, premaxilla, right and left maxillae, right and left frontals, left lacrimal, right and left prefontals, right supraorbital, right and left postorbitofrontals, parietal, right and left quadrates, parabasisphenoid, supraoccipital, right and left prootics, right and left otooccipitals, basioccipitas, left palatine, left pterygoid; MNCN BAT-3 2011–252, right and left dentaries, right and left coronoids, right surangular, right prearticular, right articular; MNCN BAT-3 2011–148, right coracoid, left humerus, right and left ilia, right and left femura, right and left tibiae; MNCN BAT-3 2011–2626, six cervical vertebrae (axis included); MNCN BAT-3 2011–2627, 20 trunk vertebrae; MNCN BAT-3 2011–2628, last trunk vertebra, two sacral vertebrae, one cloacal vertebra (plus a fragment of a possible second one), first caudal vertebra; MNCN BAT-3 2011–2629, 51 caudal vertebrae and few chevron bones. All the Batallones material except MNCN BAT-3 1088 was found associated and belongs to a single individual.

**Type locality and age.** Pikermi, near Athens (Greece). Geographic coordinates are 38°01’10”N, 23°59’30”E according to the NOW database [46]. According to [47], the late Miocene Pikermi faunal assemblage can be referred to the upper part of the middle Turolian (MN12); recent data suggests an age for the Pikermi formation between 7.37–7.11 Ma [48].

**Age and distribution.** Late Miocene (MN12) of Greece (localities: Samos Q1; Pikermi). Middle to late Miocene (MN7+8 to MN10) of Spain (localities: C2-A3, C3-A6, C3-A7 and C4-C2, Abocador de Can Mata; Batallones-3, Cerro de los Batallones).

**Emended diagnosis.** *Varanus marathonensis* differs from all the extant and other fossil *Varanus* (for which the maxilla is known) by two maxillary autapomorphies: anterior sloping surface of the facial process characterized by a medially directed, very broad and slightly concave lamina, whose medial edge can be ventrally thickened by a ridge in larger specimens; distinct lateral sulcus developed along the narial margin, anteriorly shallow but more evident posteriorly, where it is delimited by ridges. Moreover, it differs because of the following combination of maxillary characters: crista lateralis of the lateral premaxillary process not developed apically; well-developed, high and laminar crista transversalis on the medial anterior maxillary process. Additional characters: nine to 10 slender, mediolaterally flattened, non-molariform maxillary teeth; anterior edge of the facial process gently sloping (not as steep as, for example, in *Varanus exanthematicus* (Bosc, 1792)).

The phylogenetic analysis recovered the following unambiguous apomorphies for *V*. *marathonensis*: 1) Parietal, transverse posterior margin between the supratemporal processes, absent, no transverse posterior margin between the supratemporal processes; 2) Braincase, occipital condyle, single unit made of basioccipital and otooccipitals; U-shaped; 3) Prootic, facial foramen, single; 4) Prootic, accessory anterostapedial process shape, anteroposteriorly elongate tab that is laterally directed; 5) Prefrontal, anterolateral overlap of the frontal, with strong dorsomedial flange partly constricting the exposed frontal margin; 6) Groove on the lateral surface of the maxilla in the anterior sector of the bony narial opening, present; 7) Medial expansion on the anterior sloping edge of the facial process of the maxilla, present, developed on the ascending branch of the process, and well defined posteriorly (not merging with the lateral wall of the facial process).

#### Remarks

The name *Varanus marathonensis* was introduced in the literature by Weithofer in 1888 [15]. He described (on pages 291–292) and figured (in plate 19 in figs. 8–9 in [15]) two fossils from Pikermi (Fig 1A and 1B) without designating a holotype: a slab hosting what he considered to be a premaxilla, a maxilla, and a prefrontal; and an isolated supraorbital. Of these syntypes, only the slab is still available (IPUW 1888-001-001), and its direct examination allowed us to confirm with confidence the identity of the maxilla but not of the premaxilla and the prefrontal. Here we select this specimen as the lectotype of the species, whereas the supraorbital (whose current whereabouts is unknown) would become a paralectotype. It is worth mentioning that Weithofer himself remarked that a monitor lizard from Pikermi was previously reported by Gaudry in 1862 ([49] and figured in table 60, figs. 3 and 4; here shown in Fig 1C and 1D) without erecting any new species but simply writing “*Varanus*? Vertèbre qui ressemble à celles des grands varans de l’Afrique”. Such vertebra is still available in the collections of the Muséum national d'Histoire naturelle (Paris, France) with the accession number “MNHN Pikermi n. 31”. Fejérváry [50] correctly considered the material described by Weithofer as “type-remains” (see caption of his text fig. 8 in [50]) but other authors apparently overlooked the original paper by Weithofer and considered Gaudry’s vertebra as the holotype of *V*. *marathonensis* [7] or refigured it (without explicitly considering it as type material) while discussing *V*. *marathonensis* [9]. More recently, Conrad et al. [11] erroneously considered Gaudry’s vertebra as the “original holotype” of *V*. *marathonensis* and “treated the partial skull separate from the presumed holotypic vertebra because there is no clear reason to associate them”.

#### Description of the type material from Pikermi

IPUW 1888-001-001 is a left incomplete maxilla, 39.80 mm long, preserved on a block of hardened reddish sediment along with a few other unidentified bone fragments (Fig 1). The maxilla shows only the external and, partially, dorsal surfaces. The anterior portion of the maxilla is partly covered by an unidentified bone laying over it. The dorsal tip of the pars facialis and the posterior maxillary process are broken off. The general preservation is rather poor: the bone appears to be significantly mediolaterally compressed; the external surface of the maxilla is missing for about 8 mm at the level of the posterior end of the crista transversalis (a damage also visible in the original drawing by Weithofer [15]; fig 19:8); the lateral surface of the pars facialis and the area below it are severely fractured and the fragments have been apparently glued together so that a yellowish film covers most of the bone. Noteworthy is that in the above-mentioned figure accompanying the original description, these fractures appear like dendroid root traces that are actually absent (compare Fig 1A with Fig 1E). The anterior portion of the maxilla is slightly rotated in dorsal direction. Even if not perfectly preserved, the maxilla shows relevant morphologic characters. The crista transversalis is significantly raised in a dorsal direction (this could be partially due to mediolateral compression). The facial process is raised in the posterior section of the maxilla and its anterodorsal surface gradually slopes in dorsal direction. The anterodorsal sloping surface of the facial process is mediolaterally wide and hosts a deep concavity that is medially expanded. The surface of this concavity is not covered by the yellowish varnish film that covers most of the bone, but it is grayish in color, as the areas of the bones where the film was had been lost, and contrasts with the surrounding reddish matrix. The rims of the concavity are rather close to each other, probably due to compression (Fig 1F). The depth of the depression could be related to compression, but it is also possible that it was slightly excavated during preparation. In dorsal view, the medial edge of the maxilla, in the region between the crista transversalis and the medial expansion of the ascending margin of the processus facialis, is markedly concave. There is no trace of a lateral groove in the anterior sector of the narial opening (see below the section concerning the Iberian remains), probably because of the poor preservation of the bone. Due to incompleteness and breakage of the surface of the maxilla, the lateral foramina are not visible with the exception of a single foramen located close to the ventral edge of the maxilla, approximately in the middle of the preserved portion of the bone (Fig 1E). Four teeth are partly preserved along with the imprints of two additional teeth (the last one may host a tooth fragment). Teeth are triangular and pointed, but they only minimally preserve the original surface (an exception is represented by the apex of the first and third preserved teeth) so that the fine dental morphology (features such as basal wrinkles, denticles on the distal keel of the tooth, structure of the plicidentine) is no longer visible.

Weithofer [15] also described the presence of the premaxilla and prefrontal but the other bones hosted on the same block cannot be identified with confidence and therefore cannot be referred to a monitor lizard. The ‘supraciliare’ (= supraorbital) discussed and figured by Weithofer ([15], fig. 19:9) was not found. Based on Weithofer’s figure, this could be a varanid supraorbital indeed, but a precise description is hindered pending the recovery of the specimen.

In the same collection, a group of six undescribed caudal vertebrae from Pikermi were found. Although incomplete, these specimens, IPUW 5187, clearly belong to a monitor lizard. Given that they come from the type locality, these vertebrae are here referred to the hypodigm of *Varanus marathonensis* as topotypes. The three better-preserved vertebrae (Fig 2), still in connection, are described collectively below. The anterior edge of the neural arch is slightly notched and does not host any zygosphene. In dorsal view, the anterior surface of the neural arch is characterized by a triangular, deeply recessed area delimited by the ridges connecting each prezygapophysis to the neural spine. A weak but evident sagittal keel is present in the posterior sector of such area. The neural spine is developed in the posterior half of the neural arch; its dorsal development cannot be evaluated due to its incompleteness. The prezygapophyses have roundish facets that are dorsally tilted. The shape of the postzygapophyseal facets most probably matches that of the prezygapophyseal ones. A weak ridge links the pre- and postzygapophysis on the lateral side of the neural arch. The transverse processes are broken off but their remnants clearly indicate a subhorizontal orientation of their base. The ventral rim of the cotyle and condyle is placed posteriorly to the dorsal rim. The cotyle and condyle were probably dorsoventrally flattened. The ventral surface of the centrum has a median constriction (not exactly a precondylar constriction) and hosts two nearly parallel ridges delimiting between them a flat, rectangular region. The ridges originate from the cotyle and terminate into the pedestal for the chevron bone (the only preserved pedestal is the right one of the anterior vertebra). The centrum length of the best-preserved vertebra (the one in the middle) can be estimated to be about 14 mm.

The three other fragmentary vertebrae are isolated, poorly preserved and much smaller in size. The centrum length of the only sufficiently preserved vertebra nearly reaches 9 mm. Their morphology does not differ from that described for the other vertebrae, except for the fact that they are distinctly more elongate.

#### Description of the material from Abocador de Can Mata

IPS50119 is the best preserved and largest (43 mm long) of the maxillae analyzed in this study (Fig 3A–3D). The element is highly fractured but most of the fragments are still placed in their original position or have been only slightly displaced. The dorsal area of the facial process, located in the posterior half of the maxilla and preceded by a wide concavity corresponding to the naris, is the only structure that is broken off. All the external and internal surfaces of the maxilla are unsculptured. All along the lateral surface of the maxilla, close to its ventral edge, there is a row of at least ten irregularly aligned alveolar foramina extending from the first to the tenth tooth position; the last foramen is by far the largest, followed by the first one. There is no clear trend in the size of the foramina. The ventral edge of the maxilla is slightly eroded anteriorly (mostly at the level of the third tooth position). Nevertheless, in lateral view, it has no trace of supradental thickening but has a shallow concavity corresponding to the tooth positions from five to nine; in ventral view, it is nearly straight. The anterior part of the maxilla is medially expanded and forms a large lateral premaxillary process and a much smaller medial premaxillary process. The lateral premaxillary process flares in a mediolateral direction, it is apically rounded and, assuming negligible deformation, slightly curved dorsally. The medial process has an irregular medial edge, but it seems to be fairly complete; it is about five times smaller than the lateral process and pointed. The dorsal margin of the medial process is raised into a well-developed, tall and laminar crista transversalis, whereas the crista lateralis of the lateral process is not developed apically (it is present only at the base of the lateral process). The narial margin is therefore nearly smooth in the area of the lateral premaxillary process. A very shallow sulcus is developed along the narial margin in correspondence to the first two tooth positions, then its rims become progressively more elevated in posterior direction (thereby making the sulcus deeper): the lateral rim disappears on the lateral surface of the facial process at the level of the fifth alveolus; the medial rim gives origin to a distinct ridge along the posterior narial margin (on the anterior sloping edge of the facial process), which is clearly visible from the third to the seventh tooth position. The crista transversalis seems to converge with the latter ridge at the level of the fourth alveolus, where an approximately transversal breakage, with margins not perfectly fitting, splits the maxilla. The anterior edge of the lamina intercristalis corresponds to a distinct concavity developed between the lateral and the medial premaxillary processes. Posteriorly, the lamina is clearly recessed between the crista transversalis and the ridge at the posterior narial margin (crista lateralis). The anterior portion of the maxilla gradually slopes in posterodorsal direction toward the facial process, whose dorsal tip is broken off between the seventh and the ninth alveolus. The anterior, sloping surface of the facial process is mediolaterally wide and concave because it develops a medially directed, broad and slightly concave lamina (somehow the continuation of the crista transversalis), whose medial edge is ventrally thickened by a sort of ridge. This medially-expanded lamina is laterally delimited by a blunt ridge. In medial view (Fig 3B), it is clear that the lamina is not confluent with the palatal shelf. In dorsal view (Fig 3C), a deep medial concavity separates this lamina and the crista transversalis. The external surface of the facial process is weakly concave dorsally to the irregular line of the alveolar foramina. The posterior maxillary process, well preserved in the area for the contact with lacrimal and jugal, is short and stout. Its tip is posterolaterally directed. The palatal shelf, medially to the crista transversalis, is slightly broken but clearly characterized, in medial view, by a distinct ventral concavity corresponding to the third, fourth, and part of the fifth tooth positions. The palatal shelf is nearly straight from the sixth to the ninth positions, and bends downward posteriorly to the half of the ninth position. On the medial surface of the maxilla, dorsal to the palatal shelf, there are three main excavations (Fig 3B). The anterior one is delimited dorsally by the crista transversalis and ventrally by the concave sector of the palatal shelf (corresponding to the tooth positions three to five). In correspondence of the anterior margin of the fifth tooth position, such excavation hosts a large foramen (foramen at base of facial process sensu [51]). Posteriorly to the mid of the fifth tooth position, a very wide, deep second excavation, the lacrimal recess, is delimited dorsally by the above-described medial lamina of the facial process, laterally by the facial process and ventrally by the palatal shelf. The posterior excavation, for the contact with jugal, develops posteriorly to the mid of the ninth tooth position (where the palatal shelf forms a distinct step posterior to which it bents posteroventrally); it is anteriorly marked by a weak ridge, and delimited laterally by the medial surface of the posterior maxillary process and ventrally by the palatal shelf itself. The forked shape of the posterior end of the palatal shelf is probably due to breakage (Fig 3C and 3D). The palatal surface of the palatal shelf does not host any dental gutter because the shelf forms a very obtuse ventral angle with the lateral wall (with the exception of the above-mentioned area corresponding to the concavity between dental positions three to five). The tooth row is represented by 10 tooth positions, of which the first, fifth, seventh, and ninth host at least a remnant of a subpleurodont tooth (sensu [52]). The tooth in the first position is truncated close to its base; the one in the fifth position lacks only the tip; the two others are complete, but fractured. The size of the tooth positions increases up to the central ones (from five to seven) and then decreases. The first and ninth teeth are substantially smaller than the other preserved teeth. All preserved teeth are mediolaterally flattened, pointed, and posteriorly curved. They have unserrated keels. The distal keels are more evident than the mesial ones, which are apparently rather worn (the best visible proximal keel is that of the tooth in the seventh position). The orientation of the keels is not parallel to that of the lower edge of the maxilla, but slightly transversal, the tips of the teeth pointing in posteromedial direction. All the preserved teeth are characterized by basoapical grooves (developed for about half of the length of the tooth), thus betraying the presence of plicidentine. This presence is also indicated by the lamellae [53] visible on the section of the first tooth, which is truncated close to its base. Among the tooth positions not hosting teeth, the second and third are clean of matrix and the palatal shelf shows a rough surface with irregular furrows.

The second maxilla IPS50292 (Fig 3E–3H) is smaller (length of the preserved portion: 32.20 mm) than IPS50119 but shares with it all the basic features that are visible in the preserved areas not covered by matrix. It is remarkable the presence in the anterior sector of the narial opening of the lateral sulcus, which is visible in both lateral and dorsal views (Fig 3E and 3G). The lateral rim of this sulcus is more defined than in IPS50119 and, as in the latter, the lateral ridge delimiting the groove terminates on the lateral surface of the facial process, where a foramen filled with matrix is located. Even if the crista transversalis is not preserved, the remnants of the lamina intercristalis indicate the presence of a concavity shallower than that visible in IPS50119. The medially directed, broad and slightly concave lamina on the anterior, sloping edge of the facial process is comparatively more developed in IPS50292 than in IPS50119. The preserved portion of the palatal shelf clearly confirms the presence in IPS50292 of a ventral flexure terminating dorsal to the (presumed) fifth tooth position. The anterior extent of the flexure is unknown because the palatal shelf is broken off. At least eight tooth positions are preserved. Three teeth are present in the anterior sector of the maxilla. Their size is obviously different, but their shape and orientation is congruent with that described above for IPS50119.

IPS57544 is represented by two tooth-bearing bone fragments. The largest corresponds to two tooth positions, the smallest just to one. None of them preserves a complete tooth, but both show the remnants of labiolingually-compressed teeth with longitudinal basal striations. Due to erosion, such striations are well visible, and one of the two teeth of the largest fragment, broken nearly transversally, clearly shows the internal lamellae typical of the plicidentine.

None of the available four trunk and four caudal vertebrae (Fig 4A–4J) is complete nor, if nearly so, completely free of matrix. Trunk vertebrae are characterized by a proportionally massive, long centrum (the length of the posterior fragment of IPS42353 is 15.42 mm, Fig 4A and 4B; IPS43719 is 18.36 mm long, Fig 4E and 4F) with a flat ventral surface. Both the cotyle and the condyle are noticeably dorsoventrally depressed. The cotyle is 3.52 mm high and 8.90 mm wide in IPS42362; the condyle is 5.20 mm high and 10.08 mm wide in IPS42353, and approximately 3.92 mm high and 9.41 mm wide in IPS43719. Due to a different development of their dorsal and ventral rims and edges, the cotyle faces in anteroventral direction, whereas the condyle does in posterodorsal direction. The ventral margin of the condyle is located nearly at the posterior edge of the vertebra. The condyle is separated from the centrum by a marked precondylar constriction (see arrows in Fig 4B and 4F). The ratio between the width at constriction and the width of the condyle is 0.74 in both IPS42353 (7.43 mm/10.08 mm) and IPS57546 (7.58 mm/10.24 mm). As shown by IPS42362 (Fig 4C), on the neural arch there are no distinct depressions between the prezygapophyses and the basis of the neural spine, and there is no hint of zygosphene. The diameter of the neural canal is very small if compared to the vertebra (Fig 4D). The prezygapophyses are dorsally tilted, forming in IPS42362 an angle of about 110° with the anterodorsal edge of the neural arch. The prezygapophyseal facets are oval and markedly elongated in anterolateral direction. The postzygapophyseal facets are similarly oval but are posterolaterally directed. In IPS57546, the lateral development of the prezygapophyses significantly exceeds that of the postzygapophyses. The shape and orientation of the synapophyses varies according to the position of the vertebra within the vertebral column. They can be nearly dorsoventrally oriented, with a ventral edge developed in anterior direction and nearly reaching the cotyle, as in IPS43719, or relatively large, clearly visible in dorsal view, dorsoventrally flattened and anteroposteriorly developed in a nearly horizontal plane, as in IPS42362. The posteroventral surface of the neural arch does not host any zygantrum. The surface of the neural arch, lateral to the neural spine, can be finely striated. None of the dorsal vertebrae preserves a complete neural spine.

Caudal vertebrae (Fig 4G–4J) are characterized by a centrum generally provided with two weak hemal keels connecting the cotyle to the pedestals for the chevron bones placed close to the condyle. The hemal keels are not present in the fragmentary caudal vertebra IPS49408. The pedestals have an oval surface (with a main axis longitudinally developed) facing in posteroventral direction. The cotyle and condyle have a ventral edge well posterior to the dorsal edge; their shape varies according to the position of the vertebra in the tail. The cotyle is markedly flattened dorsoventrally in IPS42400. The ventral surface of the centrum is slightly convex in IPS57547 (the centrum length corresponds to approximately 12.50 mm). The elongated centrum of IPS42400, 10.30 mm long, has sides nearly parallel in ventral view, with a weak interzygapophyseal constriction placed at mid length between the synapophysis and the cotyle; there is no clear precondylar constriction (but the exposed side of the centrum preceding the condyle is slightly damaged). Similarly, IPS42368 has no precondylar constriction. It is present but only moderately developed in IPS57547, because the ratio between the width at constriction (7.18 mm) and the width of the condyle (8.43 mm) is 0.85. A depression between the prezygapophyses and the basis of the neural spine is clearly absent (as in IPS42368), but a ridge links each prezygapophysis to the base of the neural spine, therefore delimiting a concave dorsal surface at the anterior edge of the neural arch. In IPS57547, the prezygapophyses are anterolaterally directed and dorsally tilted; the shape of their facets is oval and noticeably elongated. The postzygapophyses are small, tilted and with oval facets. The postzygapophyseal facets are tilted about 45° in IPS42368. The transverse processes are laminar. Their base is nearly horizontal in lateral view, but they are apically pointing in posteroventral direction (as in IPS42400). The posterior tip of their base can be connected by a ridge to the condyle. The basis of the neural spine develops in the posterior area of the neural arch. IPS42368 shows a depression on the basal anterior surface of the neural spine. The only well-preserved neural spine is that of IPS42400. It is very tall (15.52 mm), moderately inclined posteriorly, slightly expanded distally, and nearly truncated apically.

#### Description of the isolated material from Batallones-3

The two fragmentary maxillae from BAT-3 apparently belong to a single individual. The right maxilla BAT-3 1088a (Fig 5A–5D) is better preserved than the left one, BAT-3 1088b (Fig 5E and 5F). BAT-3 1088a is represented by four fragments glued together. The total length of the maxilla is of 32.40 mm. The medial premaxillary process and part of the facial process are broken off but the palatal shelf preserves the entire series of nine tooth positions: teeth of the second, fourth, sixth, seventh, and eight positions are still in place. The morphology of this maxilla, including tooth morphology, is congruent with that of IPS50119 and IPS50292. It is remarkable the presence of the same sulcus delimited by ridges at the lateral edge of the narial aperture (Fig 5A and 5C), and the morphology of the anterior surface of the facial process (Fig 5C). The sulcus reaches posteriorly the level of the fifth interdental position. Despite its incompleteness, the anterior surface of the facial process is dorsally concave and significantly developed in medial direction all along the anterior, sloping surface of the process itself. Particularly wide is the foramen of the jugal trough which opens dorsal to the palatal shelf at the level of the last interdentary space. The posterior end of the palatal shelf is complete and not forked as in IPS50119. Probably due to breakage, the step on the palatal shelf marking the contact area with the jugal (see IPS50119) is not present.

BAT-3 1088b is represented just by a portion of palatal shelf corresponding to eight tooth positions and a little part of the facial process. Just the third preserved position hosts a tooth. None of the characters described above can be discerned on this element.

Two unnumbered isolated teeth and about a dozen bone fragments likely belong to these two maxillae.

The right prefrontal BAT-3 1088c is a rather well preserved triradiate element (Fig 6). The main body and the sublacrimal process are complete, whereas the frontal process and the maxillary flange are apically broken off. Dermal sculpturing and canthal crest on the external surface of the main body are absent, but this surface is slightly rough. The main body is dorsally expanded in medial direction and it significantly overhangs the paranasal recess. The internal surface is characterized by a deeply-excavated paranasal recess. The recess is very well delimited posteriorly (where the frontal process originates). Its smoothness is just altered by a little ridge along the maxillary flange. A little foramen opens on the posterior wall of the recess. The triangular base of the frontal process is stout and thick. The fact that it tapers abruptly could indicate that it was not particularly long. The dorsal surface of the frontal process has an evident knob. There is a little notch at the boundary between the base of the frontal process (the anterior limit of the orbital surface of the frontal process) and the antorbital flange. The latter is markedly convex. The medial surface of the tip of the sublacrimal process has a small but distinct notch, which contacted the palatine. The rim of the lacrimal foramen, located between the maxillary flange and the sublacrimal process, is wide and quite concave. The maxillary flange bears the mark left by the overlapping facial process of the maxilla. Such mark is well defined posteriorly, but rather faint dorsally. With the exception of a tiny foramen located dorsal to the rim of the lacrimal foramen, no evident foramina open on the external surface of the prefrontal. The subpalpebral fossa is also absent.

#### Description of the nearly complete skeleton from Batallones-3

The nearly complete skeleton from Batallones-3 is represented by at least 119 skeletal elements, originally found close to each other, corresponding to the following collection numbers MNCN BAT-3 2011–200, –252, –148, –2626, –2627, –2628, and –2629.

**Premaxilla–**The premaxillae (Fig 7A and 7B) are fused in a single element (MNCN BAT-3 2011–200a). They only partially preserve the long nasal process, but the overall preservation is relatively good, despite the presence of concretion partially hiding the morphological characters. The anterior edge of the premaxillae is roundish and there is no rostrum anterior to the teeth. The right ethmoidal foramen, despite being filled with concretion, is clearly visible on the external surface (more so than the left one). The internal aperture of these foramina is not visible. Three teeth are preserved to the right of the central tooth, whereas only two are visible to the left (plus a small replacement tooth); therefore at least six tooth positions are partially preserved, but their original number could have been slightly higher. Teeth are pleurodont, approximately conical, with a blunt tip and without any sharp keel. They are equal in size. A smooth edge on the right posterolateral sector of the basal plate indicates the presence of a premaxilla-maxilla aperture (due to preservational reasons, neither maxillae show the corresponding notch). The incisive process is present, but because of preservation it is not clear if it was bipartite or not. The general morphology of the basal plate and of the preserved base of the incisive process clearly indicates that the anterior tips of the missing septomaxillae were far from the anterior tip of the premaxilla. The nasal process is much more developed dorsoventrally (3.60 mm) than mediolaterally (1.40 mm). This narrow morphology is an apomorphic feature of *Varanus* according to our phylogenetic analysis. The total length of the preserved portion is 19.20 mm.

**Maxilla–**Both maxillae are available, but none of them is complete (Fig 7C–7E). Both their anterior and posterior tips are preserved. Their total length is 43.10 mm (right maxilla) and 42.80 mm (left maxilla). The presence of a concretion hinders the perception of some of the finer morphological details. The concretion adhering to the medial side of the right maxilla embeds two as yet unidentified skeletal fragments that partially cover the maxilla. The tooth positions can be counted with confidence only on the left maxilla, where they are nine, but the same number is likely valid also for the right maxilla. The latter still hosts some teeth or their remnants in the positions one, three, five seven and (just a very basal portion) nine. The left maxilla hosts teeth in the positions one to five and then seven. In both maxillae, the concretion keeps approximately in place some small replacement partial teeth: two teeth are visible in the space of the second tooth position, whereas on the left element two teeth precede and follow the second tooth.

Both maxillae are characterized by the medially expanded ascending surface of the facial process and by the lateral sulcus developed along the narial margin. The medial expansion of the facial process is testified by its remnants that are preserved nearly at the dorsal tip of the facial process of the left maxilla (which is not laminar but has a medial, yet broken, expansion; Fig 7C and 7E). The expanded lamina has two main depressions located dorsal to the third and sixth tooth positions. The narial edge of the maxilla is nearly horizontal dorsal to the first four positions, then steep dorsal to the fifth and sixth positions, and is markedly steeper dorsal to the seventh and eight positions (that is to say, up to the dorsal tip of the facial process). The lateral sulcus developed along the narial margin extends on both maxillae from the second to the fourth tooth position (Fig 7D).

The left maxilla preserves the tip of the facial process, which corresponds to the eight tooth position. In lateral view, it is apically rounded and it overhangs the deeply concave lacrimal recess.

**Palatine–**A nearly complete left palatine (Fig 7F and 7G) is preserved adhered by matrix to the ventrally overlapping palatine process of the pterygoid (BAT-3 2011–200s). It is a triradiate element only slightly longer than wide. The complete vomerine process is an elongate laminar structure directed anteromedially but also slightly ventrally. The robust maxillary process is only partly preserved; it is anterolaterally directed but morphological details are not visible for preservational reasons. The pterygoid process is directed posteriorly, comparatively wide, laminar and it bifurcates apically. The medial edge of the palatine, linking the vomerine and the pterygoid process, is markedly concave, without any medial expansion. The palatine groove is short without any secondary palate. The presence of concretion at the level of the anterior infraorbital continuation of the maxillary process betrays the probable location of the palatine foramen. Palatine teeth are absent. This absence is apomorphic for *Varanus* in our phylogeny.

**Pterygoid–**A left pterygoid is nearly completely preserved (Fig 7H and 7I), being represented by the main body, the palatine and tranverse processes and most of the quadrate process, which misses its basal portion. The basypterygoid buttress and the columellar fossa are not preserved. The tips of both the palatine and tranverse processes are incomplete (the one of the palatine process is preserved along with the palatine BAT-3 2011–200s). There are no pterygoid teeth. As for the missing palatine teeth, the absence of pterygoid teeth is an apomorphy of *Varanus* (characters 394 and 395 in the phylogenetic analysis have been scored as “?” in the corresponding coding in agreement with the scores for the other *Varanus* species in the matrix, but these characters are actually non-applicable because none of the *Varanus* species have pterygoid teeth). The transverse process is rather thick and tall laterally. Its lateral edge is flattened and develops dorsally in a sort of lamina and ventrally in a rounded expansion. The lateral margin of the transverse process is convex, whereas the medial one, hosting the contact facet with the ectopterygoid, is concave. Both the dorsal and ventral surfaces of the main body of the pterygoid are somewhat concave. The edge of the pterygoid between the transverse and the palatine processes is laminar and concave. The posteromedial edge of the transverse process is thicker than the anterolateral one. A distinct ridge runs along the quadrate process, presumably at the ventromedial edge, and the medial surface is weakly concave dorsal to it.

**Ectopterygoid–**BAT-3 2011–200r is a right, partially preserved ectopterygoid (Fig 7J and 7K). Even though it is incomplete, this element is clearly slender and elongate. The anteriorly placed dorsal pterygoid process is more complete than the posterior region, which preserves only the cavity that hosted the transverse process of the pterygoid. By the general architecture of the palate, it is clear that the ectopterygoid contacted the palatine anterior to the suborbital fenestra.

**Lacrimal–**A left lacrimal, rather well preserved (Fig 7L and 7M), was found in the lacrimal recess of the maxilla. It is 10.30 mm long. The posterolateral flange, constituting the anterior margin of the orbit, is distinctly developed in a distinct, rounded process whose lateral surface is slightly rugose. The anterior edge hosts laterally the contact surface with the maxilla, and medially that of the prefrontal, whereas the posteroventral tip hosts that of the jugal. At the edge of the ventromedial surface, there is a distinct notch for the contact with the palatine. The ventral opening of the lacrimal foramen [54], filled with sediment, seems to be rather large and opens close to the posteroventral border of the element, by the contact facet with the palatine.

**Quadrate–**The two quadrates are well preserved. The left element (Fig 7N–7P) is more complete than the right one, lacking only a small ventral portion of the medial crest. These two elements are taller than broad, 19.30 mm and 10.60 mm, respectively. The posterior crest originates ventral to the wide surface for the articulation with the squamosal. It is robust and deeply concave in its dorsal sector. The lateral, or tympanic crest, straight both in lateral and posterior views, is well developed, being only slightly shorter than the maximum width of the posterior crest. The medial crest, slightly curved in medial view, develops in anteromedial direction giving origin to a deep fossa on the anterodorsal surface of the bone. The lateral rim of the fossa is marked by an evident ridge. The saddle-shaped articular condyle shows a lateral hemicondyle much smaller than the medial. The latter develops a process in anteromedial direction, at the base of the medial crest.

**Prefrontal–**Both prefrontals are preserved (Fig 8A and 8B). The right element (21.20 mm long, 18.30 mm high) is more complete, but also more fractured, than the left one. Their morphology is congruent with that already described for BAT-3 1088c, with some minor differences related to the absence of a knob on the dorsal edge of the bone and the absence of foramina on both the external and internal surfaces. As in BAT-3 1088c, there is no evident sculpturing of the external surface, but a V-shaped scar corresponding to the contact with the supraorbital is present at the dorsolateral edge of the left element (the right one is not well preserved in that area). As in BAT-3 1088c, there is no evidence for the subpalpebral fossa.

**Supraorbital–**The right supraorbital is nearly complete (Fig 8C and 8D). Its mediolateral length is 13.30 mm. It has the shape of an elongated, posterolaterally directed spine. The proximal end, deeper posteriorly than anteriorly, is much thicker than the pointed tip. The dorsal surface is slightly irregular close to the proximal end, where both the anterior and medial edges are distinctly crenulate. The vaguely triangular medial surface is centrally smooth but crenulate at the edge (it is eroded in the posteroventral sector).

**Frontal–**The two paired frontals (Fig 8E–8I) are well preserved, and the left one (32 mm long, 12.10 mm wide) is nearly complete. Their dorsal surface is vaguely sculptured. The lateral edge of the dorsal surface is nearly straight, but at mid length it shows a small lateral expansion corresponding to the dorsal notch in the neighboring prefontal. The anteriorly tapering tip of the left element preserves a medial, partly broken, lamina that underlay the nasal, and also the lateral surface, laterally bordering the nasal, that represents the posteromedial edge of the osseous external naris. The contact between the (probably paired) nasals and the frontal is W-shaped. The posterior tips of the nasals were therefore separated from each other and tapering posteriorly in an apically rounded point. There are no nasofrontal fontanellae. The lateral surface hosts anteriorly a marked, wide, V-shaped contact surface for the prefrontal, whereas close to the posterior edge there is the contact surface with the frontal process of the postorbitofrontal. The medial surface is proportionally very thick (4 mm) and characterized by fine striations interlocking in the two opposed frontals when articulated. The posterior surface hosts a markedly interdigitated suture for the parietal. At least the median region of this sutural contact is convex (both the frontals have broken posterolateral edges). Even though they are incomplete in both frontals, the cristae cranii are represented by well-developed, ventromedially directed laminae well delimiting the olfactory tract. The crista of the right frontal, better preserved than that of the left counterpart, is well developed in medial direction, reaching the level of the medial margin of the dorsal surface of the bone (Fig 8I). This indicates that the cristae were ventromedially in contact in the posterior region of the frontal. The intertemporal width is of about 11.60 mm. The parietal tabs are present (an apomorphy of *Varanus* in our phylogenetic analysis).

**Postorbitofrontal–**Both the right and left elements preserve their anterior portion, which is characterized by the three evident processes: the anterolateral process, the frontal process and the parietal process (Fig 8J and 8K). The anterolateral processes are robust and pointed with a dorsal surface distinctly irregular. The posterior surface of the frontal process and the medial surface of the parietal process are complementary to that of the bones they contact: the first one is approximately concave, whereas the latter is sloping in ventromedial direction. The left postorbitofrontal also preserves part of the posterior ramus (the squamosal process), the relative robustness of which indicates that it was very long originally (Fig 8J and 8K). The lateral surface of this process hosts the elongated contact facet with the squamosal. Anteriorly, an obtuse angle is formed by the anterolateral and frontal processes, as in *V*. *mokrensis* [5]. In contrast with the latter species, however, the squamosal and parietal processes form a very wide angle in the Iberian specimen and the margin between them is distinctly concave (the angle is acute and the margin is V-shaped in *V*. *mokrensis* [5]). Moreover, the angle made by the frontal and parietal processes is strongly wider in *V*. *mokrensis* compared to the specimen from Batallones. The postorbitofrontal does not contact the jugal nor the prefrontal.

**Parietal–**This unpaired bone is nearly perfectly preserved (Fig 8L and 8M). Only the tips of the supratemporal processes, the right one more extensively than the left one, are broken off. It is a nearly X-shaped element, with a nearly straight anterior edge and deeply concave lateral and posterior margins. It is 30.70 mm wide at the anterior edge and 33.30 mm long and it is characterized by a marked thickness (about 7.30 mm at the level of the pineal fossa). The intertemporal width corresponds to 12.40 mm. The dorsal surface is flat and its anterior half is slightly vermiculate. The anterior edge has a medial, small anterior projection that slightly separates the frontals from each other. The pineal foramen pierces the parietal just a few millimeters from its anterior edge, well anteriorly to the posterior edge of the facets for the contact with the postorbitofrontal. The lateral edges of the parietal anteriorly host the facets for the contact with the postorbitofrontal and posteriorly form the deeply concave rim of the supratemporal fossae. The surface delimiting the fossae is concave anteriorly but only lateroventrally sloping posteriorly. In the latter area, a thin, approximately longitudinal ridge develops close to the dorsal edge of the parietal.

The supratemporal processes are very long (they are only slightly shorter than half the total length of the parietal) and their preserved tips are 33.80 mm from each other, so that the anterior width of the bone is smaller than the posterior one. The processes are directed posterolaterally in dorsal view and slightly ventrally in lateral view. The posterior angle between the processes is about 90°. Their short axis is ventrolaterally directed. They are somewhat rounded dorsally (but not broad or flat) and rather sharp ventrally. The facets for the contact with the supratemporal extend anteriorly at the level of (on the left) or well beyond (on the right) the anterior limit of the processes, and occupy most of their lateral surface. The contact facets at the distal tip of the supratemporal processes are not preserved or visible with confidence, except for the supratemporal facet that could be present on the left process. The ventral surface of the parietal hosts at its lateral edges, anteriorly to the supratemporal processes, the descensus parietalis (or crista cranii parietalis), a moderately deleveloped crest nearly anteroposteriorly oriented. The fossa parietalis opens posteriorly and is delimited ventrally by a well-developed ridge, the crista juxtafovealis that gives origin to the crista postfovealis, running along the medial surface of the supratemporal processes.

**Braincase–**The braincase is represented by the isolated parabasisphenoid (Fig 9A–9D) and by a complex including the supraoccipital, the prootics, the otooccipitals and the basioccipital (Fig 9E–9I). The complex is not well preserved, being highly fractured (in some cases with little dislocation) and partly covered by concretion. The left paroccipital process is separated from the rest of the braincase (Fig 9J and 9K) and the presence of a crystalline concretion on the contact surface prevents its reconnection. For preservational reasons, the right side of the complex is more informative than the left one.

The parabasisphenoid is much wider than long. Its ventral surface (as well as that of the basioccipital) is smooth and devoid of any sagittal ridge or recess (Fig 9B). The basipterygoid processes are long, anteroventrally directed, apically expanded and rounded. Their medial surface is convex, whereas the lateral one is flattened. Part of the base of the parasphenoid process and of the trabeculae cranii is preserved. This portion of the bone hosts dorsally two longitudinal ridges, the cristae trabeculares, separated by a sagittal groove. Another groove laterally borders each of the ridges. The ventral surface is smooth. The left trabecula cranii is nearly complete. Lateral to the base of the trabeculae cranii, the small anterior openings of the Vidian canal are located on both sides, being therefore significantly ventral to the dorsum sellae. The latter is rather shallow. The posterior opening of the Vidian canals is clearly visible on the left side: it is completely hosted by the parabasisphenoid. The abducens canals (cranial nerve VI) pierce the bone on the dorsum sellae, close to its anterolateral edge, and on the anterior surface, just medial to the alar processes. The alar processes are robust and anteroventrolaterally directed. The pituitary fossa is filled with concretion and therefore the openings of the carotid canals are not visible. The posterior articulation facets for the basioccipital are tall and robustly built. There is no clear entocarotid fossa within the recessus vena jugularis dorsal to the base of the of the basipterygoid processes of the basisphenoid. The posterolateral flanges of the parabasisphenoid are not developed.

The sutures between the supraoccipital and the surrounding elements are well visible (Fig 9H and 9I). This unpaired element is vaguely pentagonal and characterized by a dorsal surface raised in a broad sagittal ridge. The anterior tip of the supraoccipital, the processus ascendens, is anterodorsally oriented and apically truncated. Lateral to the anterior truncation, it hosts the two contact facets for the lateral rims of the fossa parietalis. The internal surface of the supraoccipital, close to the suture with the prootic, it shows a convexity corresponding to the anterior semicircular canal. Posteriorly, the supraoccipital forms the dorsal margin of the foramen magnum.

Both the prootics preserve the long, anterodorsally and relatively pointed alar process, each distinctly overhanging the relative inferior process (Fig 9E). The fenestra ovalis, as well as the foramen rotundum, cannot be seen directly because of the concretion covering it. However, its absence in the area anterior or dorsal to the sphenooccipital tubercle indicates that it is located posterior to it. For the same reason, it has not been possible to evaluate whether the facial foramen is single or double (but it is single in the remains from Samos attributed to *V*. *amnhophilis*; [11]). The prootic contribution to the paroccipital process extends posterolaterally over more than two-thirds of the process (Fig 9F and 9J). The well-developed right prootic crest clearly hosts a well-preserved accessory anterostapedial process, which is anteroposterioly elongate and ventrolaterally expanded. The internal surface of the prootics is markedly convex in correspondence to the semicircular canals.

The tip of the right paroccipital process is not complete but the isolated portion of the left one is perfectly preserved and shows that its dorsal and ventral tips extend equally posterolaterally (Fig 9J and 9K). The ventrolateral margin of the exoccipital part of the left paroccipital process has an accessory lip, which is distinctly offset with a dorsolateral step. On the external surface of the otooccipital, lateral to the dorsal edge of the occipital condyle, as well on the inner surface, between the rim of the occipital canal and the auditory bulla, the openings of the X and XII cranial nerves are present but obstructed by concretion. On the posterior surface, they are apparently hosted in the same depression, whereas on the internal surface they are clearly separated by a well-defined ridge.

Both sphenooccipital tubercles are preserved. They are evident but comparatively small and clearly directed ventrally. They are placed at the anteroventral tip of the tuberal crest. The occipital condyle is proportionally strong and very well defined. It is made ventrally by the basioccipital and laterally by the otooccipitals. The general shape is that of a broad U (Fig 9H).

The tuberal crests (Fig 9E) are laterally eroded (the left one much more that the right one), but it is clear that they inserted on the paroccipital process much more laterally than on the sphenooccipital tubercle. The crests are anteroventrally inclined and, in ventral view, they cover completely the laterally open recessus scalae tympani. The latter is better preserved on the right side of the braincase, but it is nearly completely filled with concretion and therefore most of the fine anatomical details cannot be seen.

**Dentary–**The two dentaries are isolated and rather well preserved (Fig 10A–10C). They are relatively robust, being proportionally tall and massive (i.e., not as slender and thin as in *Varanus salvator*). The right element (42.60 mm; Fig 10C) is more informative than the contralateral (38 mm; Fig 10A and 10B). It has ten tooth positions, seven of which still preserving teeth or their fragments (only the fifth, seventh and ninth positions do not host teeth). This number of tooth positions could be congruent with the arrangement shown by the left dentary, whose preservation hinders a precise count. The dorsal edge of the dentaries is clearly concave, whereas the ventral edge is convex. In dorsal view, they are rather straight, but the anterior tip is slightly curved medially. Their lateral, convex surface hosts some mental foramina. In the right dentary, there are six foramina. They are approximately aligned and the last one, corresponding to the posterior edge of the last tooth position, is by far the largest. Due to preservational reasons, only three foramina can be counted on the left element. In both dentaries, a small longitudinal sulcus is developed on the anterolateral surface, ventral to the first three positions and dorsal to the first foramen. The medial surface is characterized by the absence of a subdental ridge (the subdental table, sensu [55], is clearly sloping in medioventral direction with a smooth surface) and by a Meckel’s canal completely open along the entire length of the dentary. The canal is rather narrow in the anterior half of the dentary, where it is restricted to the ventral edge. It reaches the symphysial area, giving to the latter a kidney shape/reniform outline in medial view. The intramandibular septum terminates posteriorly to the last tooth position. Its posteroventral margin is sutured along the dentary wall. It is not clear if and how the dentaries contributed to the anterior inferior alveolar foramen. The morphology of the teeth fits with that described above for those of the maxillae from both the Vallès-Penedès Basin and Batallones. The posterior end is poorly preserved in both dentaries and the posterior processes are broken away.

**Surangular, prearticular and articular–**Due to fossilization, the right surangular, prearticular and articular are preserved together in a single unit 58.30 mm long (Fig 10D–10G), but they are not all fused together, being most of the sutures among the bones well visible. Their general preservation is relatively good and they provide significant morphological information.

The surangular, which is nearly complete, missing only part of the anterior tip and the area dorsally expanded below the coronoid, is robust. In dorsal view, it is distinctly convex laterally and concave medially. On the anteroventral surface, the surangular has an elongated facet that was overlapped by the missing angular. The ventral portion of this facet is developed on the prearticular. The anterior surangular foramen is visible just ventral to the area that contacted the coronoid. Despite being anteriorly preceded by a shallow concavity that was overlaid by the lingual flange of the coronoid’s anterior process, it is not associated to a groove. It seems likely that this foramen was dorsally bordered by the latter element. The posterior surangular foramen, which clearly opens entirely on the surangular, is close to the surangular-articular suture. The adductor fossa is comparatively small, not expanded, and has a low medial margin not producing a vertical flange. The posterior canal (sensu [56]) opens on the medial surface of the surangular, close to the articular. Anterior to the adductor fossa, the surangular has a drop-shaped facet that was overlapped by the posteriorly directed medial process of the coronoid. The prearticular does not show any crest. The articular forms the glenoid surface without any contribution from the surangular. This surface is characterized by a large, bulbous convexity mostly developed in anteromedial position, associated with a vaguely concave area that borders it posteromedially and posteriorly. The retroarticular process is rather long, massive, not posteriorly expanded, and slightly directed posteromedially.

**Coronoid–**Both coronoids are preserved (Fig 10H and 10I), but the right one is represented only by part of the dorsal eminence and part of the anterior processes. The latter process is rather long and relatively robust. The coronoid eminence, or coronoid process, is relatively tall and characterized by having a clearly crenulated posterodorsal edge. On the external surface, it has a well-marked ridge, oriented posterodorsally-anteroventrally, that reaches the mid of the preserved portion of the labial flange of the anterior process. The labial flange seems to be rather complete in both coronoids (in the left one the dorsal notch between the labial and lingual flange is preserved), and by superimposing it on the surangular it is clear that in labial view it did not overlap the posterior margin of the coronoid process of the dentary. The labial flange is more developed in ventral direction than the lingual flange. The posterior process of the left coronoid is represented only by a small portion, triangular in cross section, of the original process. The preserved portion of the left coronoid is 25.10 mm long.

**Vertebrae–**The atlas of the almost complete skeleton is missing, but the axis and the following six cervical vertebrae are preserved (BAT-3 2011–2626; Fig 11A–11E). They are all characterized by having a posteroventrally directed hypapophysis or at least a vestige of it. The hypapophyses of the axis and the cervical vertebrae from four to six are apically notched. The one of cervical vertebra three is rounded (Fig 11B and 11D). The cervical vertebrae are longer than the dorsal vertebrae. Twenty dorsal vertebrae are available (BAT-3 2011–2627; Fig 11F–11J). They are characterized by the absence of zygosphene-zygantra articulations (an apomorphic feature of *Varanus* in our phylogeny) and by a strong precondylar constriction. The ratio bewteen the minimum precondylar width of the centrum and the maximum condyle width varies, in the four vertebrae that can be measured, from 70.8% to 75.6% (8.5–12.0 = 70.8%, 8.8–11.9 = 73.9%, 9.0–11.9 = 75.6%, 8.3–11.6 = 71.6%). The centrum length is difficult to measure because most of the vertebrae are preserved in connection. Nevertheless, the length of the longest centrum may reach 15.30 mm.

A block of vertebrae cemented by matrix (BAT-3 2011–2628; Fig 12) contains the last trunk vertebra (with rather pointed synapophyses), the two sacral vertebrae, one cloacal vertebra (plus a fragment of a possible second one), and the first caudal vertebra. A further element could represent the proximal portion of the right ischium. There are also a metatarsal and a partial phalanx.

The first sacral vertebra has massive, laterally broad diapophyses (maximum width at the level of the diapophyses is approximately 39 mm, but the left one is not complete). The distal end of the right diapophysis has the shape of a V laying on one side, with the concavity oriented in posterior direction. The dorsal branch of the V is more robust and longer than the ventral one. The second sacral vertebra is better preserved and exposed than the first one. The estimated length of the centrum could reach approximately 15 mm. The diapophyses are slightly bent downward and their distal tip is simply anteroposteriorly elongated. The maximum width at the level of the diapophyses in the second sacral vertebra is of 37.50 mm. Two short longitudinal (following the main axis of the structure) ridges are located at mid length of each diapophyses, close to its posterodorsal and posteroventral edges. At least one vertebra associated to the sacral ones is interpreted as a cloacal vertebra lacking pedestals for chevron bones and synapophyses, but bearing transverse processes. It also lacks lymphapophyses.

Nearly all the tail is preserved on a slab where 51 vertebrae can be counted (BAT-3 2011–2629; Fig 13), with a gap of one vertebra before the last ten preserved ones. Very few chevron bones are associated to the vertebrae 21 to 26. No caudal vertebra shows any evidence of autotomic plane. All the caudal vertebrae are characterized by a single pair of long transverse processes, and by well-developed pedestals for the chevron bones. The pedestals are located slightly anterior to the posteroventral margin of the centrum.

**Coracoid–**A partial right coracoid is preserved (Fig 14A and 14B). The wide coracoid blade is fractured but nearly complete. It forms the posterior rim of a deep secondary coracoid fossa. Even though the anterior rim of the latter is not complete, it is possible to state that it was relatively small, about the size of the glenoid fossa. The coracoid foramen is located anteroventrally to the glenoid fossa. The thinning of the bone anterior to the coracoid foramen indicates the location of the primary coracoid fenestra. The shallow glenoid fossa is preserved, as is the surface contact with the scapula.

**Humerus–**Only the left humerus is available (Fig 14C–14F). Even if incomplete, it preserves both the proximal humeral head and the distal epiphysis, which are developed on nearly orthogonal planes. The total length of the humerus is 59.40 mm. An elongated unidentified bone is embedded in the concretion covering the humeral surface posterior to the deltopectoral crest. The head is significantly extended in posteroventral direction. The single deltopectoral crest is well developed in dorsal direction. The stout shaft is elliptical in cross section, with the main axis oriented anteroposteriorly (length of the axis = 7.80 mm), but widens considerably in distal direction (the width of the distal epiphysis is 19.80 mm). The entepicondilar fossa is rather deep and wide. The ectepicondyle is nearly completely broken off, but the hint of a groove can be seen on its ventral surface. The presence or absence of the entepicondilar and ectepicondylar foramina cannot be assessed because of preservational reasons. The radial condyle is thinner but much more developed than the ulnar condyle. The presence of distal articulations clearly indicates the presence of the non-preserved zeugopodial elements. As a whole, the humerus appears rather robust, similarly to the humerus of *Varanus* sp. reported by Georgalis et al. [57] from the late Miocene of Ravin de la Pluie. These authors suggested a short and stout-limbed morphology for Neogene varanids from Europe and the humerus of the Batallones specimen might support this conclusion, at least for *V*. *marathonensis*. Given that the very few fossil *Varanus* limb bones from the European continent cannot be clearly identified at species level based on our current knowledge, it is not possible to clearly state whether only *V*. *marathonensis* possessed this limb morphology or it was shared with other species.

**Ilium–**Both ilia are preserved (Fig 15A and 15B). The anteroposterior length of the right element is 42.40 mm. The anterior (preacetabular) process is stout, short and anterodorsally directed. The rod-like dorsal process is rather straight in lateral view, but moderately curved, with the tip pointing posterolaterally, in dorsal view. It bears a number of longitudinal striations. A main ridge is developed on the dorsal edge of this process, extending from the anterior process to about the mid of the dorsal process. Here, another ridge, located more medially than the former, originates and extends up to the posterior tip of the ilium. The iliac portion of the acetabulum cavity is wide and weakly delimited.

**Femur–**Both femora are preserved, but none is complete. The right element, BAT-3 2011–252f (Fig 15C–15F), is 65.30 mm long and lacks the femoral head. The left one, BAT-3 2011– 252e (Fig 15G and 15H), is 67 mm long and lacks most of the tibial condyles. The femoral head is expanded anteroposteriorly. The internal trochanter is well developed and stout. It is connected to the femoral head by a slightly concave surface. The diaphysis is proportionally robust (dorsoventral thickness of the left element: 8.20 mm), straight and nearly cylindrical, being only slightly flattened ventrally. The distal epiphysis is moderately expanded (20.40 mm), with two tibial condyles well separated by a distinct trochlea.

**Tibia–**The proximal end of a right tibia and a nearly complete left tibia are preserved. The complete element is 46.80 mm long (Fig 15I and 15J). The proximal epiphysis is dorsoventrally flattened and mediolaterally wide (18.90 × 11.20 mm). Both sides of the proximal epiphysis bear a robust ridge. The diaphysis is slightly dorsoventrally flattened. Its maximum diameter reaches 6.40 mm. It widens slightly towards the distal epiphysis, which is 11.10 mm wide.

**Fibula—**This robust element is represented by its proximal portion (Fig 15K), characterized by a vaguely drop-shaped articulation.

### Phylogenetic analysis

**Fig 16:**
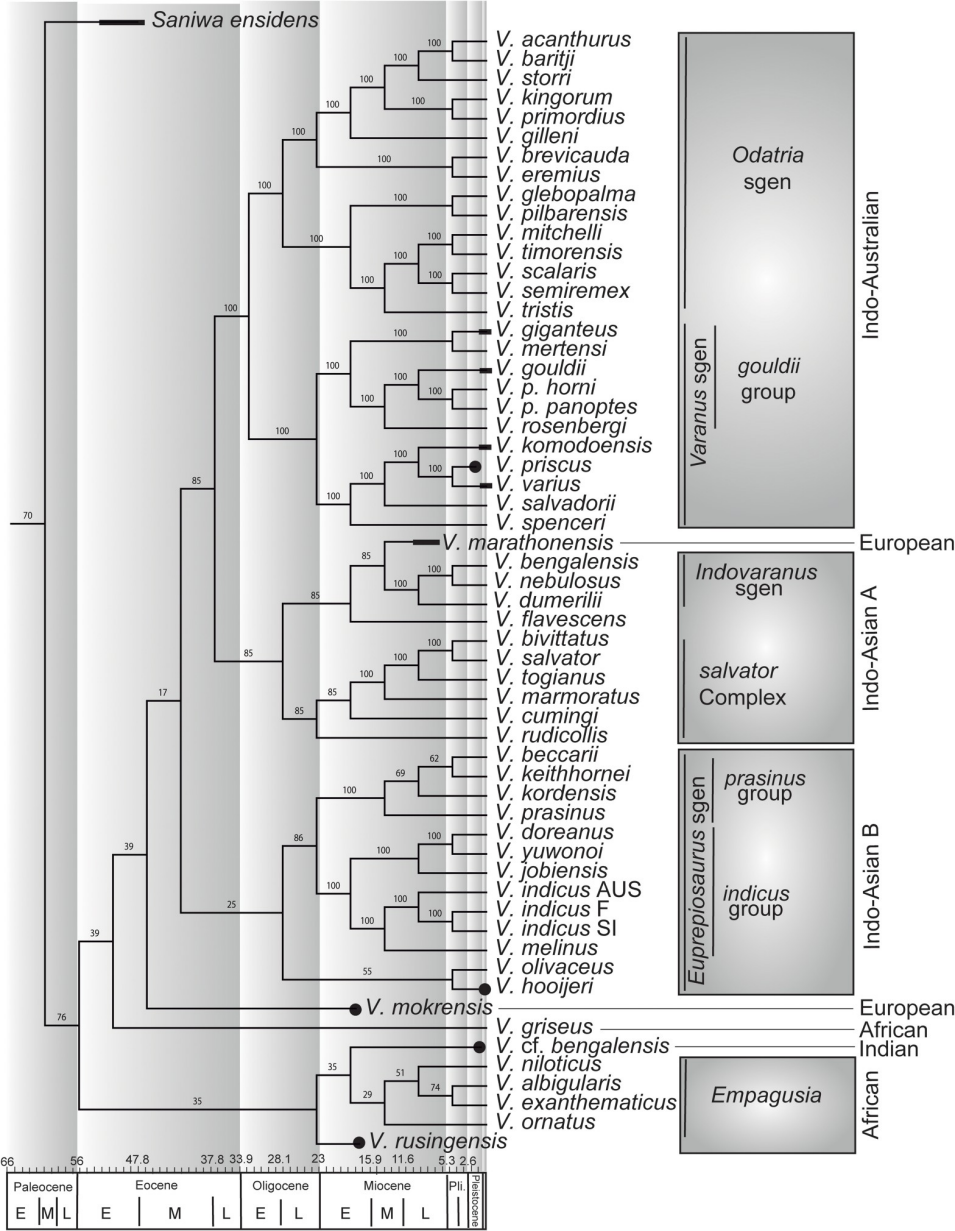
Majority-rule consensus tree based on the results of analysis 1A. The position of *Varanus marathonensis* (as scored from information provided by the specimen from Batallones and the type of *Varanus amnhophilis*; this OTU is named “Vmarathonensis Vamnhophilis” in figures of the supplementary information) inside the Indo-Asian A group and as sister species to the *Indovaranus* subgenus is supported by 85% of the MPTs. Numbers in the time scale represent million years ago.

The overall topology of our analyses is very similar to that recovered by Conrad et al. [11]. In their Adams consensus tree, Conrad et al. [11] recovered many of the traditional groups, but failed to recover Ast’s [58] general topology where Indo-Asian A clade and Indo-Asian B clade formed the sister group to the Indo-Australian clade. Conrad et al. [11] and our analyses here (Fig 16) recover as a general topology an African clade (also recovered by Ast [58], but with the inclusion in the former group of the fragmentary Indian *Varanus* cf. *bengalensis*, which is the name given by Conrad et al. [11] to the Indian fossils close to *Varanus bengalensis* mentioned by Fejérváry [50], in our results) that is sister group to a large group formed by Indo-Asian B, Indo-Asian A clade and the Indo-Australian clade. The Indo-Asian A clade is sister to the the Indo-Australian clade, and the Indo-Asian B clade is sister to the group formed by both of them.

Analysis 1A (see Material and Methods) recovered 2397 trees with a length of 10642 steps (Consistency Index = 0.311, Retention Index = 0.452). The strict consensus (see S5 File) does not recover a monophyletic *Varanus* and shows no resolution within the genus, in the same way as in Conrad et al. [11], except for the monophyletic Indo-Australian clade. The Adams consensus tree (see S5 File) presents a much better resolved phylogeny for *Varanus*, with the general topology as described above. Regarding the position of *V*. *marathonensis* (merged with *V*. *amnhophilis*; "Vmarathonensis Vamnhophilis” in the supplementary files), it forms a trichotomy with the group formed by *V*. *bengalensis* + *V*. *nebulosus* + *V*. *dumerilii* + *V*. *flavescens* and the *V*. *salvator* complex + *V*. *rudicollis*. The majority rule consensus tree (see S5 File) shows, however, that up to 85% of the trees recover *V*. *marathonensis* as the sister taxon of the group formed by *V*. *bengalensis* + *V*. *nebulosus* + *V*. *dumerilii*. In analysis 2A (see S6 File), and regarding the European fossils, *V*. *amnhophilis* and *V*. *marathonensis* (as coded from the specimen from Batallones) are recovered as sister taxa, suggesting they might represent the same species. Alternatively, this could be interpreted as indicating a high degree of phylogenetic closeness, but still different species. The evidence reported in the present paper (see Discussion) suggests the former interpretation is correct. The Adams consensus tree recovers *V*. *amnhophilis* and *V*. *marathonensis* in the same position as *V*. *marathonensis* (coded as merged: "Vmarathonensis Vamnhophilis") in analysis 1A. In any case, more than 50% of the trees (as reported by the Majority rule consensus tree) support a position inside the Indo-Asian clade, and, more specifically, again as sister to the group formed by *V*. *bengalensis* + *V*. *nebulosus* + *V*. *dumerilii*. The second set of analyses (with two new characters added; not figured) yielded trees that are two steps longer, but similar in terms of topology and percentage of trees supported in the majority rule consensus trees. A list of the apomorphies recovered for *V*. *marathonensis* and the synapomorphies of the clade containing *V*. *marathonensis* and the subgenus *Indovaranus* as well as those of the *Varanus* clade are given in the supporting information (S7 File).

## Discussion

### The taxonomic status of *Varanus marathonensis* Weithofer, 1888

**Fig 17:**
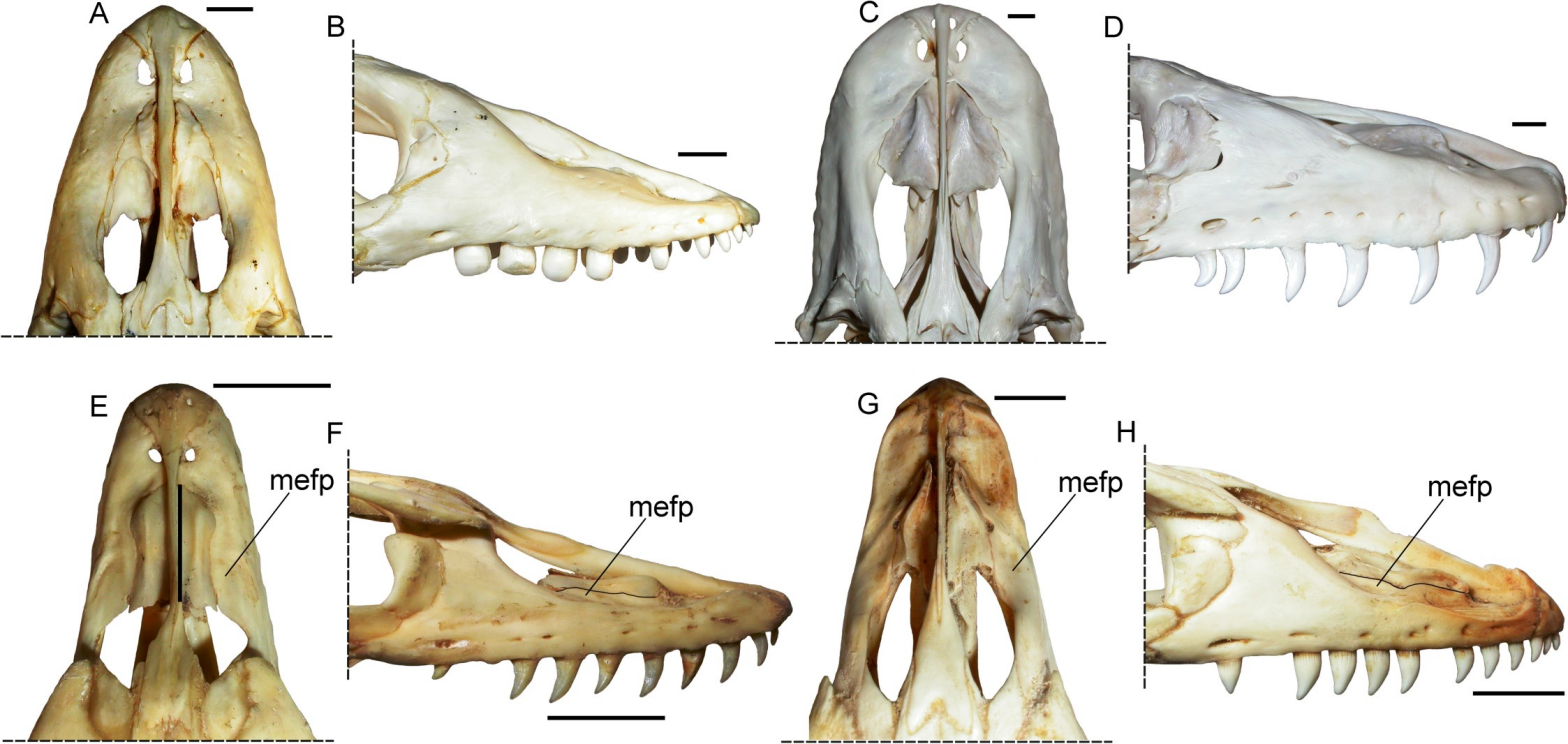
Snouts of extant *Varanus* species showing the maxillary morphology in dorsal and right lateral view. (A, B) *Varanus ornatus* (Daudin, 1803) ZFMK 14889. (C, D) *Varanus komodoensis* Ouwens, 1912 ZFMK 64998. (E, F) *Varanus griseus* (Daudin, 1803) SMF 32911. (G, H) *Varanus bengalensis* (Daudin, 1802) SMF 63456. Note in all cases the absence of a lateral sulcus along the edge of the narial aperture. In *V*. *ornatus* and *V*. *komodoensis* there is no hint of medial expansion of the medial edge of the maxilla (no medial concavity along the medial edge of the maxilla in dorsal view), whereas in *V*. *griseus* and *V*. *bengalensis* a sort of expansion is mostly located in the area anterior to the facial process (presence of a variably developed concavity). Among the four species here depicted, *V*. *ornatus* is the only one that bears blunt, molariform teeth. Scale bars equal 10 mm.

The recovery of monitor lizards remains in the late Miocene of Pikermi, close to Athens (Greece), was already mentioned by Gaudry [49], who prudently referred them to as *Varanus* sp. [49,59,60]. Few years later, Weithofer [15] carefully described and figured a few *Varanus* bones from the same locality, Pikermi, and erected *Varanus marathonensis* on this basis. One of the characters described by Weithofer [15] clearly refers to the fact that the anterodorsal surface of the facial process does not have a more or less sharp edge, but instead is wide, forming a channeled depression. Direct observation of the lectotype IPUW 1888-001-001 confirms the presence of this depression. As noted above (see descriptions of the material in the Systematic Paleontology section), this character is present in all the fossil maxillae from the Miocene of Abocador de Can Mata and Cerro de los Batallones. The only significant difference is that, in the lectotype from Pikermi, the medial expansion of the anterior, sloping edge of the facial process is narrower and its concavity more accentuated. This is, however, possibly attributable to the mediolateral compression of the specimen (see also the comments by de Fejérváry [50]), the Iberian material (in particular IPS50119) showing the actual, undistorted morphology (Figs 3C, 3F, 3G and 5C). Such expansion of the anterior edge of the facial process has not been described for any extant *Varanus* species [10,61–65]. Our own examination of the maxillae of 249 specimens of 37 extant *Varanus* species (see S1 File) confirms that, despite significant interspecific variability (compare morphologies in Fig 17), none of these species has a facial process showing this morphology. Similar—but not identical—morphologies are displayed by *Varanus griseus* (Daudin, 1803) and *Varanus bengalensis* (Daudin, 1802), whose maxillae are briefly described below.

The maxilla of *V*. *griseus* (Fig 17E and 17F) is long and rather low in dorsoventral direction, the crista transversalis is underdeveloped, and there is no sulcus or ridge lateral to the naris aperture. In dorsal view, the medial edge of the maxilla is distinctly concave (as in *V*. *marathonensis*) in all studied specimens (from the smallest, ZFMK 21657, to the largest, ZFMK 7848; length of the maxilla is 22.60 mm and 37.50 mm, respectively). The concavity, which hosts a lateral expansion of the septomaxilla, is parallel with the medial concavity and ventral folding of the palatal shelf. In the two disarticulated specimens of *V*. *griseus* (CM 145053, SMF 74486) examined, it is clear that the dorsal lamina is not confluent with the palatal shelf (as in *V*. *marathonensis* IPS50119) and that a foramen opens between the medially expanded lamina and the palatal shelf. However, the medially expanded area (posterior to the concavity) is only partly located on the anterior sloping surface of the facial process and is mostly developed on the horizontal surface, anterior to the facial process itself. This is different from the situation in *V*. *marathonensis*, in which it develops on the sloping surface. Such widened surface, significantly smaller than in *V*. *marathonensis*, develops nearly horizontally (not inclined as in *V*. *marathonensis*) and can be slightly concave (an exception being ZFMK 7848). The surface is delimited anteriorly by a variably developed step (located at the level of the fifth tooth position) and frequently also by a lateral ridge, even in the smallest available specimen (in which there is clearly a vestige of lateral ridge). An exceptionally developed ridge is present in SMF 74486. In lateral view, the step corresponds to a small dorsal convexity of the upper profile of the maxilla. In ZFMK 7848, this surface is nearly flat on the left maxilla and irregularly concave on the right maxilla, where the bone clearly shows a healed fracture that probably resulted in the depression.

*Varanus bengalensis*, in turn, has a very peculiar morphology (Fig 17G and 17H). The crista transversalis can be very high and nearly vertical. The medial edge of the maxilla is raised in a variably high lamina that is already present in juveniles, but is fully developed in the large specimens. The lamina intercristalis, between the crista lateralis and the crista transversalis is distinctly depressed. Anteriorly to the facial process, there is a medially directed lamina with a slightly dorsally concave surface. This lamina is variably developed. It is nearly absent in SMF 11550 (maxillary length 9.90 mm) and SMF 11554 (maxillary length 19.10 mm), weakly developed in SMF 40179 (maxillary length 28.50 mm), and it reaches its maximum development in the largest specimens, such as SMF 60428 (maxillary length 46.40 mm) and SMF 63456 (maxillary length 36.60 mm). An exception is represented by SFM 71569 (maxillary length 45.10 mm), in which the surface is much less developed than in other specimens of approximately the same size. This lamina abuts (it is not overlapped by) the septomaxilla and does not join ventrally the palatal shelf (as in *V*. *griseus* and *V*. *marathonensis*). However, the development of such lamina does not reach the extent seen in *V*. *marathonensis* and, in dorsal view, there is only a week medial concavity between the lamina and the crista transversalis.

On the basis of the medial development of the sloping surface of the facial process of the maxilla, it is here concluded that *Varanus marathonensis* Weithofer, 1888 is a taxonomically valid, diagnosable species. Moreover, thanks to the new Iberian material, it is possible to associate other characters to *V*. *marathonensis* (see Diagnosis). It is noteworthy that a lateral sulcus in the anterior sector of the narial opening is present. It is a character unknown in any other fossil *Varanus* species so far described nor in any of the extant *Varanus* species directly examined during this study (see for example Fig 17).

Finally, all the teeth of *V*. *marathonensis* clearly show the presence of plicidentine [53] and are mediolaterally flattened, slender, and apically pointed. This dental morphology distinguishes this species from those in the clade of the extant African taxa, except for *V*. *griseus* (the morphology of *Varanus ornatus* (Daudin, 1803) is shown in Fig 17B). It also distinguishes it from the fossil species *Varanus rusingensis* Clos, 1995 (early Miocene, 17.8 Ma, Rusinga Island, Kenya), the maxillary morphology of which is not known in detail, but that is characterized by the presence of blunt posterior teeth [66].

### Taxonomy of the European monitor lizards

The question remains as to how many monitor species from the Neogene of Europe can be considered valid from a taxonomic viewpoint. Although other eight nominal species have been erected on the basis of European Neogene materials, it is likely that *V*. *marathonensis* and *V*. *mokrensis* are the only European species so far described on the basis of diagnostic remains. Most of the remaining nominal species are either junior synonyms of *V*. *marathonensis* or, being based on isolated vertebrae that are not diagnostic at the species rank, best considered nomina dubia (if belonging to *Varanus* at all).

**Fig 18:**
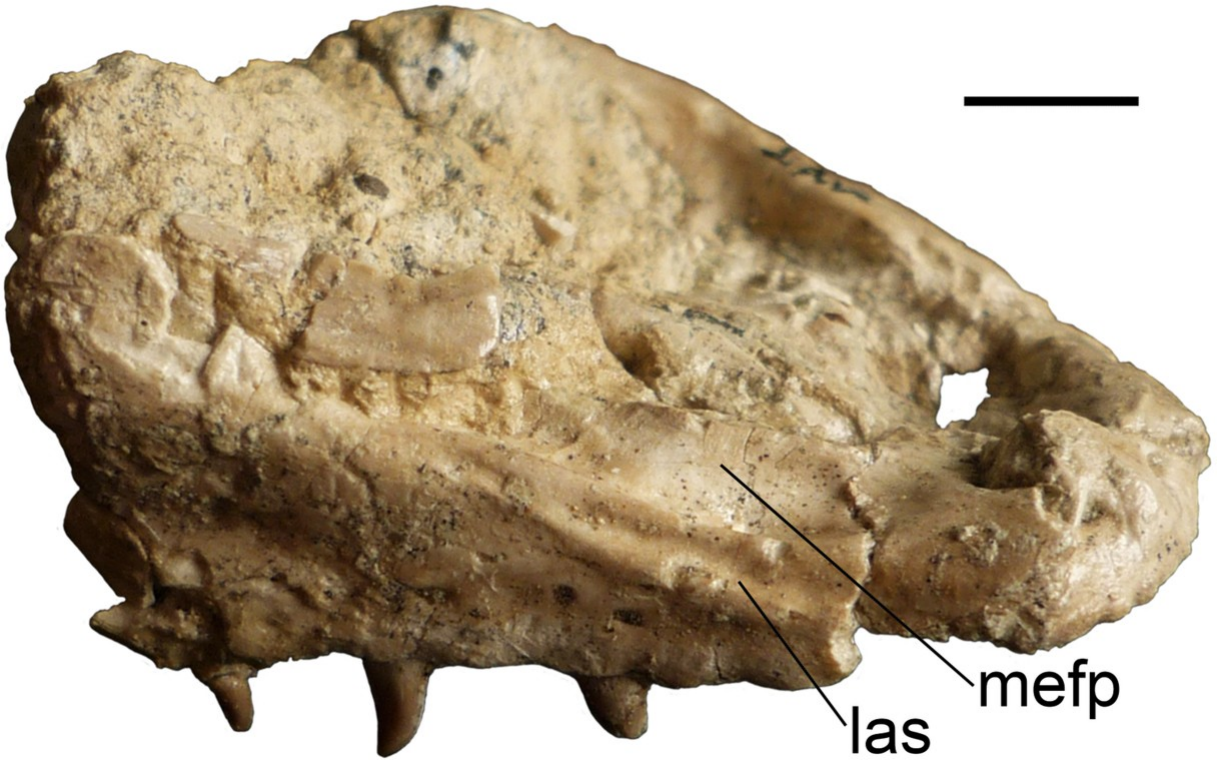
Partial skull (ΜΥΤ-130, including the anterior portion of the skull) of *Varanus marathonensis* Weithofer, 1888 from Samos in right dorsolateral view. Scale bar equals 5 mm.

The erection of the new species *Varanus amnhophilis* Conrad et al., 2012 from Samos was anticipated by Conrad et al. [12], and information about its morphology was indirectly provided by Conrad et al. [10] via a preliminary character scoring. Due to the wide chronological and geographic range of *V*. *marathonensis*, and chronological and geographic proximity of the Q1 locality of Samos and Pikermi (the type locality of *V*. *marathonensis*), Delfino et al. [16,17] suggested that the new purported species from Samos had to be compared with *V*. *marathonensis* before formal erection. However, Conrad et al. [11] separated the name of *V*. *marathonensis* from its diagnostic cranial type material (the slab IPUW 1888-001-001, hosting at least a diagnostic maxilla) and associated it to Gaudry’s non-diagnostic (at least at species level) vertebra. This opened the way to the erection of the new species *V*. *amnhophilis* without comparing it with Weithofer’s material and with the European fossil record of *V*. *marathonensis*. The diagnosis of *V*. *amnhophilis* is non-differential, but based on a combination of apomorphies that we know now are shared with *V*. *marathonensis* (except for the few characters that cannot be evaluated on the skeletal elements currently available for *V*. *marathonensis*). Further support for the synonymization of *V*. *amnhophilis* with *V*. *marathonensis* is provided by additional, still unpublished material from Samos, hosted in the collections of the Natural History Museum of Aegean, Mytilinii, Samos Island. This material, which will be the subject of a forthcoming publication, comes from another locality of the Mytilinii Formation and comprises the anterior region of a skull (Fig 18). It includes both the maxillae that are characterized by the lateral sulcus along the narial margin and the medially expanded ascending facial process typical of *V*. *marathonensis*. Accordingly, *Varanus amnhophilis* Conrad et al. (2012) is here considered a junior subjective synonym of *V*. *marathonensis* Weithofer, 1888. It is worth mentioning that the size of the Samos remains described by Conrad et al. [11] is identical to that of *V*. *marathonensis* from ACM, at least on the basis of the centrum length (presumably the ‘dorsal vertebral length’ of Conrad et al. [11]) of the dorsal vertebra IPS43719 which reaches 18.36 mm. This pushes the earliest occurrence of a giant monitor lizard back to at least the middle Miocene (*V*. *mokrensis* from the early Miocene is a medium-sized species [5]).

*Varanus atticus* Nopcsa, 1908 was introduced in the literature just to name the vertebra already described and figured by Gaudry [49] as *Varanus* sp. Nopcsa [67], who did not significantly comment further about his new species, was apparently not aware that Weithofer [15] had already described a valid species on the basis of other material from the same locality. Coming the remains described by Gaudry and Weithofer from the same locality, and being therefore likely conspecific, the nominal taxon *Varanus atticus* Nopcsa, 1908 is here considered a junior subjective synonym of *Varanus marathonensis* Weithofer, 1888, as previously concluded by de Fejérváry [50], Hoffstetter [68], and Rage & Sen [69]. Note that these nominal species are not objective synonyms, as they were erected on the basis of different type materials.

The status of *Varanus deserticolus* Bolkay, 1913 is rather doubtful. It was erected on the basis of a fragmentary dentary with two teeth (one of them complete, [70]: tab. 12/2) and an isolated vertebra from the Pliocene of Beremend (Hungary; [70]: fig 2). The vertebra was briefly described as having tilted prezygapophyseal facets. According to de Fejérváry [50] and Młynarsky [71], it actually belongs to an anguid lizard. The dentary tooth was described as ‘rather flattened, and nearly straight, blunt at the end’ ([70], p. 222). However, the figure does not show any blunt tooth and, all in all, it does not allow confirming the referral of the dentary to a monitor lizard. On the basis of the information provided by Bolkay [70], it is not possible to support the taxonomic validity of *V*. *deserticolus*, which, due to the poorly informative nature of the type and only known material, is best considered a nomen dubium. Estes [7] and Molnar [9] listed this nominal species as a junior synonym of *V*. *marathonensis*, but such opinion is not followed here, because the type material of *V*. *deserticolus* does not show the diagnostic features of *V*. *marathonensis* reported in this paper.

The type material of *Varanus hofmanni* Roger, 1898 is represented by vertebrae characterized by a depression, with an angulate posterior end, developed in the anterior region of the neural arch (see also [72,73]). This species has been recorded, sometimes with doubts, from a number of localities on the basis of nearly only isolated vertebrae (see among others: [72–76]). However, due to the poor knowledge of the variability of the vertebral morphology in extant *Varanus* [2,10], it is not possible to discern whether these remains are conspecific or not with those referred here to *V*. *marathonensis*. As such, *V*. *hofmanni* should be better considered a nomen dubium.

The characteristics of the Eastern European species *Varanus lungui* Zerova & Chkhikvadze, 1986, *Varanus semjonovi* Zerova & Chkhikvadze, 1986 and *Varanus tyrasiensis* Lungu, Zerova & Chkhikvadze, 1983 have been summarized and discussed by Molnar [9]. These species, so far only reported from their respective type localities, are all based on few isolated, variably fragmentary vertebrae. They should be similarly considered nomina dubia for the same reasons that have been discussed above. It is worth mentioning that Zerova & Chkhikvadze [77] themselves recognized that the character on which *V*. *tyrasiensis* was originally erected was actually variable in the comparative material that they had later at their disposal.

The recently described *Varanus mokrensis* Ivanov et al., 2018 is based on a large collection of both cranial and postcranial elements and, as such, can easily be compared with *V*. *marathonensis*. The single autapomorphy of this species, the acute angle between the parietal and squamosal processes of the postorbitofrontal, is absent in *V*. *marathonensis*, the processes of which make in contrast a widely obtuse angle. Based on the figured maxilla and on the descriptions given by Ivanov et al. [5], it is not possible to clearly infer the presence and morphology of a medial lamina on the anterior margin of the facial process of *V*. *mokrensis*, but the lateral sulcus along the narial margin is distinctly absent. Moreover, *V*. *marathonensis* differs from *V*. *mokrensis* in other significant features, among which: parietal with narrower anterolateral processes (reflected in a narrower angle between the frontal and the parietal processes of the postorbitofrontal), straight anterior margin (convex in *V*. *mokrensis*), and a pineal foramen that is closer to the anterior margin; absence of a distinct crest on the base of the paroccipital process as seen in posterior view; slightly less prominent iliac crest on the ilium. *Varanus mokrensis* is also distinctly smaller in size [5]. Admittedly, some similarities are also present between the two species. These include a gently sloping anterior edge of the facial process, the obtuse angle between the frontal and anterolateral processes of the postorbitofrontal, a similar morphology of the dentition, similar inclination and robustness of the preacetabular process of the ilium, and postzygapophyses forming a right angle in the trunk vertebrae. Taking all of this into consideration, it is well justified to consider *V*. *marathonensis* and *V*. *mokrensis* as distinct species.

*Varanus catalaunicus* (Hoffstetter, 1969) is a nomen dubium. This species was erected by Hoffstetter [73] within the newly erected genus *Iberovaranus* Hoffstetter, 1969 on the basis of an isolated trunk vertebra. However, Delfino et al. [78] later demonstrated that the morphology of this vertebra fits with the variation seen in *Varanus*. Because of this, they synonymized *Iberovaranus* with *Varanus*. The vertebral material described by Hoffstetter [73] and Delfino et al. [78] is not significant enough for a specific diagnosis. We here follow Delfino et al.’s [78] view in considering *V*. *catalaunicus* as a nomen dubium. Nevertheless, it is interesting to note that the size of the holotypic vertebra of *V*. *catalaunicus* is strongly smaller than the largest trunk vertebrae of *V*. *marathonensis* (10.60 mm contra 18.36 mm), approaching the vertebral size of a medium-sized monitor lizard like the roughly coeval *V*. *mokrensis* from the Czech Republic.

To sum up, *V*. *marathonensis* and *V*. *mokrensis* currently seem to be the only taxonomically valid European species of monitor lizard, the other nominal taxa previously erected from the European Neogene being either junior synonyms (*V*. *amnhophilis*, *V*. *atticus*) of the former, or nomina dubia. However, the only remains clearly referable to *V*. *marathonensis* are those represented at least by maxillae or sharing with *V*. *marathonensis* a significant portion of cranial characters, i.e., those from Pikermi (Greece), Abocador de Can Mata (Barcelona, Spain), Batallones-3 (Madrid, Spain), and Samos (Greece). The referral to *V*. *marathonensis* of very fragmentary cranial elements, isolated vertebrae, or sections of vertebral columns from the early Pliocene of Csarnóta 1 (Hungary; [50]) and Çalta (Turkey; [69]) should be confirmed by well-preserved cranial material. These remains are here provisionally referred to *Varanus* sp. A further, particular case is that of the dentary from the late Pleistocene of Arene Candide (Italy). Identified as *V*. *marathonensis* by Morelli [79], it is currently lost, but according to its original description and figure it most likely does not belong to a monitor lizard [80].

### Phylogenetic relationships

Even though there is a general morphology- and molecule-based consensus on the monophyly of *Varanus*, including the extinct *Varanus priscus* (Owen, 1859), the internal phylogeny of the genus is still unclear [10,58,81,82]. The mitochondrial phylogeny by Ast [58], recently qualified as “probably the most comprehensive and reliable study inferring relationships among extant varanid lizards” [83], grouped the extant species in three clades concordant with their geographic distribution: African, Indo-Asian, and Indo-Australian. However, such arrangement has not been replicated by the morphology-based analyses performed by Conrad et al. [10,64]. Combined data sets of morphological and molecular data gave different results depending on the inclusion or not of fossil taxa (compare fig. 26 with 27 by Conrad et al. [10]).

Such unstable phylogeny seriously hampers deciphering the phylogenetic relationships between European fossil monitor lizards and extant taxa. A priori, it would be expected to find close relationships between the European taxa and the clade of the African forms [58], which comprises *V*. *griseus*, a species with a geographic range that currently approaches Europe, including also the southern and eastern Mediterranean countries [84,85]. Actually, on the basis of superficial similarities, Levshakova [86] proposed to include *Varanus marathonensis* in the same clade as *V*. *griseus*, along with another extinct species, *Varanus darevskii* Levshakova, 1986, from the Pliocene of Tadjiikistan.

Conrad et al. [11] recovered their *V*. *amnhophilis* in the Indo-Asian A clade and what they considered to be the type of *V*. *marathonensis* (namely, Gaudry’s vertebra) nested into the Australasian clade. The cranial material that actually represents the lectotype of *V*. *marathonensis* (labeled ‘*Varanus*’ cf. *marathonensis* in their tree) fell well outside of crown *Varanus* (probably related to the large amount of missing data). Based on their phylogenetic results, Conrad et al. [11] suggested a Eurasian origin for the genus *Varanus* as a whole and Asian affinities for the European species. These hypotheses seemed to be confirmed by the phylogenetic analysis of Ivanov et al. [5], in which *V*. *mokrensis* was found as a member of the Indo-Asian A clade but is basal to African varanids, including the extinct *V*. *rusingensis* from the early Miocene of Kenya. All these analyses found *V*. *amnhophilis* and *V*. *marathonensis* (both Gaudry’s and Weithofer’s material) to be phylogenetically well apart.

Problems arising from the inclusion of fragmentary remains in phylogenetic analyses based on large datasets have been already stressed (see e.g., [6]). As explained above, our morphological comparisons suggest that the skeleton from Batallones belongs to the same species as *V*. *amnhophilis*, and the position of the clade composed by them inside the Indo-Asian A clade is rather stable. More specifically, a sister group relationship with the clade formed by *V*. *dumerilii*, *V*. *bengalensis*, and *V*. *nebulosus* is well supported. Regarding *V*. *mokrensis*, it is invariably recovered as the most basal non-African taxon, sister to the group formed by the Indo-Asian B, Indo-Asian A and Indo-Australian groups (however, note that *Varanus* cf. *bengalensis*, which is Indian, is recovered nested within the Africa clade in our analysis, although it is fragmentary and thus rather unstable). This would strongly suggest that European *Varanus* include at least two different lineages: one would be represented by *V*. *mokrensis*; the other one would be represented by, according to our results, *V*. *marathonensis*.

The position recovered here for *V*. *mokrensis* is different from that recovered by Ivanov et al. [5]. However, their analysis is not completely comparable to the one presented herein because: 1) they only used morphological characters; 2) they based most of their interpretations on results of the Bayesian analysis, because their parsimony analyses provided poor resolution; 3) regarding their parsimony analyses, they removed some unstable taxa and, in some cases, used implied weighting; 4) we have scored some characters for *V*. *mokrensis* that were not scored by Ivanov et al. [5]; and 5) they coded two separate taxa, *Varanus mytilini* and *Varanus amnhophilis*, when the former actually was the name for *V*. *amnhophilis* in Conrad et al.’s [11] matrix, and therefore the same taxon was included twice (with two different scorings) in the same analysis.

### Paleobiogeography

The historical biogeography of *Varanus* is one of the most debated issues concerning the evolution of varanid lizards [83,87]. The description of Eocene isolated vertebrae from Egypt as the oldest remains clearly referable to *Varanus* [2] could support an African origin of the genus, in agreement with the basal position of the African *Varanus* taxa in molecular phylogenies [58,88]. However, a Eurasian origin in the Paleocene or earlier, followed by a later (Eocene) over-water dispersal could be equally supported (see discussion in [81]), as apparently suggested by recent phylogenies including both fossils and extant species [5, 11]. Smith et al. [89] also discussed possible Palaeogene varanid dispersals between Eurasia and Africa.

It has to be noted, however, that our phylogenetic analysis is neither supporting a Eurasian origin for *Varanus* nor the alternative hypothesis of an African origin for the genus. The sister taxon to *Varanus* recovered in most of the recent phylogenetic analyses, including the one we are presenting here and that performed by Conrad et al. [11], is *Saniwa ensidens*, from the Eocene of North America. The roughly coeval European *Saniwa orsmaelensis* was not included in the phylogenetic analysis but *Saniwa* went extinct in Europe at the end of the Eocene [90], whereas *Varanus* appears in the European fossil record only in the early Miocene [78]. The fact that the oldest varanid fossil could come from North America (Varanidae indet., Ellesmere Island, Canada [91] does not support an American origin of *Varanus*.

Moreover, in both our analysis and in Conrad et al.’s [11], the most basal *Varanus* clade is composed by African taxa. Thus, an African origin of *Varanus* cannot be discounted. However, the position we recovered for *V*. *mokrensis* (sister to all non-African *Varanus*) is at odds with the ancestral condition inferred by Ivanov et al. [5] for the African lineage. Immigration of reptiles from Africa to Europe and/or Asia in the early Miocene has already been suggested, e.g., for chamaeleonids [92], and therefore a similar biogeographical pattern might apply to *Varanus*. Our cladistic analysis recovered *V*. *mokrensis* and *V*. *marathonensis* as belonging to two different lineages without a direct ancestor-descendant relationship, thus suggesting that a different geographic origin for the two valid European *Varanus* species may be possible.

As for the origin of the European Neogene populations of *Varanus*, Rage [93] suggested that they might have originated from both Asia and Africa, as it has been shown for the European fossil cobras [94]. According to Conrad et al. [11] and Ivanov et al. [5], the phylogenetic relationships of *Varanus* species from the Neogene of Europe (*V*. *mokrensis*, *V*. *marathonensis*, and *V*. *amnhophilis*, the latter being herein considered as a synonym of *V*. *marathonensis*) indicate a possible link with an Indo-Asian clade of *Varanus*. This fits with the hypothesis of a Eurasian origin of the genus. The presence of a *Varanus* of Asian origin in the European Neogene agrees well with other, well-documented dispersal events of the herpetofauna from Asia into Europe (see [95], and references therein). The cases of hylid frogs, ranids of the group of the brown frogs, pelodytid frogs, and bufonid toads are especially noteworthy [96], and so are modern colubrids, large vipers, and elapid snakes [97,98], most of which dispersed into Europe during the relative thermal maximum of the latest early Miocene (MN4). A later Asian origin has been recently proposed by Oaks [99] for the European populations of *Crocodylus*, which dispersed into this region during the Miocene [100].

Moreover, on the basis of paleomammalogical data, it is well established that several major dispersal events took place between Europe, Asia and Africa during the early Miocene. In the lower part of the early Miocene, only an inner-Eurasian continental faunal exchange was possible [101], whereas during the Burdigalian the Afro-Arabian plates collided with Anatolia, thus enabling further faunal exchange between Africa and Eurasia (the so-called *Gomphotherium* landbridge [101, 102]. Soon thereafter (or simultaneously with) the first dispersal of proboscideans from Africa into Eurasia during the MN3 (ca. 19 Ma), several mammalian groups of African origin apparently followed the same dispersal route, including tubulidentates, hyracoids, thryonomyd rodents and catarrhine primates [102,103,104]. Although dispersals between Europe, Asia and Africa continued, the initial establishment of a land bridge connection between Afro-Arabia and southwest Asia agrees well with the first appearance datum of *Varanus* in the European fossil record, which according to the present knowledge ([3–8,78,105,106] and references therein), corresponds to the Burdigalian (latest early Miocene), at the beginning of MN4 or even slightly earlier during MN3.

Although, on the basis of available diagnostic material, the presence of *Varanus marathonensis* can be only verified in the Iberian Peninsula and Greece, it seems most likely that this species was more widely distributed across Europe. The wide geographic range of this species, coupled with its long chronological persistence (about 5 Myr), from the MN7+8 (12.0 Ma; middle Miocene) in Spain to the MN12 (ca. 7 Ma; late Miocene) in Greece, suggests that many of the available European Miocene *Varanus* remains might belong to this species. Regardless, although *Varanus marathonensis* was distributed at relatively low latitudes in both Western and Eastern Europe from the MN7+8 to the MN12, it emerges that the genus *Varanus* displayed a greater chronological and geographic distribution in this continent when all the European record is taken into account.

From a chronological viewpoint, the last record of *Varanus* in Central Europe corresponds to the late Miocene, whereas in Southern Europe it survived until the Middle Pleistocene [4]. As commented above, the Late Pleistocene datum of Arene Candide (Italy) is actually not based on *Varanus* material. It would be interesting to test whether the post-Miocene material, especially the Quaternary one, belongs to *V*. *marathonensis*, testifying its prolonged permanence in at least the southern part of Europe, or whether it represents a dispersal of other species, more closely related to extant taxa. In the case of the last occurrence of a varanid in Europe (few isolated remains, possibly pertaining to a juvenile individual, from Tourkobounia 5 in continental Greece [4]), an attribution to *V*. *marathonensis* is ruled out by the absence of the lateral sulcus in the maxilla. Nevertheless, the Middle Pleistocene Greek material is similar to *V*. *marathonensis* in the presence of a medial lamina on the maxilla and in the dental morphology [4].

From a latitudinal perspective, European fossil *Varanus* almost reached the 49^th^ parallel (Petersbuch, Germany) between MN4 and MN10, i.e. for an extended period of time broadly encompassing the Mid-Miocene Climatic Optimum [8,105]. Remarkably, in Western Europe, the last occurrence datum of *Varanus* corresponds to an unspecified site in the area of Moreda (Pliocene, MN15 to MN16, Spain; [106,107]), which is both at low latitude (N 37.4°) and close to Mediterranean Sea. This fact testifies for a range contraction that fully parallels the one experienced by other thermophilous reptiles, such as crocodylians, agamids, amphisbaenians and scolecophidians [8,93,100,108–113].

## Conclusions

*Varanus marathonensis* Weithofer, 1888, originally erected on the basis of late Miocene material from Pikermi (Greece), is currently one of the two only European *Varanus* species that can be considered taxonomically valid. The other is *V*. *mokrensis* from the early Miocene of the Czech Republic. The revision of the syntype of *V*. *marathonensis* (the maxilla IPUW 1888-001-001, here designated as the lectotype) allowed us to confirm the presence of a character already originally described by Weithofer [15], which has not been found in any known extinct or extant species of *Varanus*. The description of previously unpublished conspecific materials, including maxillae, from the latest middle Miocene stratigraphic series of Abocador de Can Mata (Vallès-Penedès Basin, Catalonia, Spain) as well as from the late Miocene of Cerro de los Batallones (Madrid Basin, Madrid, Spain), confirms the validity of *V*. *marathonensis* and implements the diagnosis of this taxon with the inclusion of several new characters. The majority of the remains from Cerro de los Batallones 3 can be referred to a single individual that, being represented by nearly all the skeletal elements, is the most complete *Varanus* skeleton ever described in the world.

The phylogenetic relationships of *V*. *marathonensis* could be sought in an eastern clade of *Varanus* instead of the African clade comprising *V*. *griseus*, to which it had been related in the past. Regardless of its phylogenetic relationships, our results indicate that *V*. *marathonensis* was distributed both in Western and Eastern Europe at least for about 5 Myr, from the middle to the late Miocene, whereas on the whole the genus *Varanus* inhabited Europe at least from the early Miocene to the late Miocene in Central Europe, further persisting until the Middle Pleistocene in Southern Europe. Due to such a wide geographic and chronological range of *V*. *marathonensis*, most of the European fossils of *Varanus* may belong to this species, at least as far as the Neogene occurrences are concerned. Therefore, comparisons with this taxon are required before erecting additional extinct European species of *Varanus* in the future. Nevertheless, the presence in Europe of a second valid species, *V*. *mokrensis*, in the early Miocene and of remains not clearly attributable to *V*. *marathonensis* in the Middle Pleistocene demonstrate that other species were indeed represented in the continent.

## Supporting information

S1 File List of extant comparative specimens of *Varanus*. (PDF)

S2 File List of references consulted to score characters for the phylogenetic analysis. (PDF)

S3 File Matrix used in the phylogenetic analyses. (NEX)

S4 File New characters added to the original list of Conrad et al. [11]. (PDF)

S5 File Results of the phylogenetic analysis 1A. (PDF)

S6 File Results of the phylogenetic analysis 2A. (PDF)

S7 File Lists of the apomorphies of *V*. *marathonensis*, of the synapomorphies of the clade including *V*. *marathonensis* and the subgenus *Indovaranus*, and of *Varanus* (based on analysis 1B). (PDF)
