# Supplementary material for: Revision of Varanus marathonensis (Squamata, Varanidae) based on historical and new material: morphology, systematics, and paleobiogeography of the European monitor lizards
Source: PLoS One. 2018 Dec 5;13(12):e0207719. doi: 10.1371/journal.pone.0207719 (PMC6281198; doi:10.1371/journal.pone.0207719)
Supplement: S1 File — (PDF) [file pone.0207719.s001.pdf]

Institutional abbreviations

LACM, Natural History Museum of Los Angeles County; MDHC: Department of Earth Sciences, University of Turin (Italy); MNCN, Museo Nacional de Ciencias Naturales–CSIC (Madrid, Spain); MNHN, Muséum national d'Histoire naturelle (Paris, France); NHMW, Naturhistorisches Museum Wien (Austria); PIMUZ, Paläontologisches Institut und Museum, Universität Zürich (Switzerland); SMF, Senckenberg Museum Frankfurt (Germany); SMNS, Staatliches Museum für Naturkunde Stuttgart (Germany); TMP, Royal Tyrrell Museum (Drumheller, Canada); ZFMK, Zoologisches Forschungsmuseum Koenig (Bonn, Germany).

List of extant comparative specimens of *Varanus*

Varanus acanthurus – SMF 11639, SMF 11642, ZFMK 5225, ZFMK 54252

Varanus albigularis – SMF 11543, SMF 21573, SMF 26580, SMF 34049, SMF 40162, SMF 54758, ZFMK 5138, ZFMK 83428

Varanus beccarii – SMF 11637

Varanus bengalensis – LACM 159019, LACM 159057, LACM 159074, LACM 159071, LACM 163952, LACM 163954, MNHN 1883-1828, MNHN 1886-634, MNHN 1886-649, SMF 11550, SMF 11554, SMF 32956, SMF 40160, SMF 40179, SMF 60428, SMF 63456, SMF 71569, SMF 71570, SMNS 11145, TMP 85.16.5, TMP 90.7.360, ZFMK 14872, ZFMK 14873, ZFMK 59018, ZFMK 70425

Varanus caudolineatus – SMF 40086

Varanus cumingi – SMF 11577, SMF 76293

Varanus doreanus – SMF 32290, ZFMK 83429

Varanus dumerilii – SMF 11556, TMP 90.7.271, TMP 90.7.272, TMP 90.7.362, SMF 11557, ZFMK 14876, ZFMK 14877

Varanus eremius – SMF 11648

Varanus exanthematicus – LACM 159111, LACM 163950, MDHC 335, MNHN 1910-7, MNHM 1952-132, SMF 11544, SMF 11545, SMF 33260, SMF 33261, SMF 40161, ZFMK 14884, ZFMK 17528, ZFMK 21652, ZFMK 38432, ZFMK 63663, ZFMK 63664, ZFMK 76976, ZFMK 76977

Varanus flavescens – MNHN 1964-51, SMF 11546, SMF 54157, ZFMK 14878, ZFMK 21653

Varanus giganteus – SMF 53263, ZFMK 14882, ZFMK 84341

Varanus gilleni – SMF 11627

Varanus glebopalma – ZFMK 54847

Varanus gouldi – SMF 11620, SMF 53276, SMF 59018, ZFMK 14885

Varanus grayi – SMF 72156

Varanus griseus – MNHN 1880-4, MNHN 1888-196, MNHN 1895-366, MNHN 1909-402, MNHN 1973-105, PIMUZ A/III 0248, SMF 32911, SMF 33205, SMF 33206, SMF 33254, SMF 33255, SMF 33256, SMF 33257, SMF 33702, SMF 40163, SMF 70678, SMF 74486, SMF 79190, TMP 90.7.47, ZFMK 7848, ZFMK 14883, ZFMK 21657, ZFMK 53533

Varanus indicus – NHMW 739, SMF 32180, TMP 90.7.45, ZFMK 14863, ZFMK 14864, ZFMK 14865, ZFMK 14866, ZFMK 14867, ZFMK 14881

Varanus jobiensis – SMF 75817

Varanus komodoensis – LACM 121971, SMF 23189, SMF 37209, SMF 57555, SMF 57556, SMF 68133, SMNS 9132, ZFMK 64698

Varanus marmoratus – SMF 11571

Varanus mertensi – SMF 53275

Varanus mitchelli – ZFMK 54250

Varanus niloticus – MNCN 40853, MNHN 1887-909, MNHN 1909-20, MNHN 1921-260, MNHN 1921-260 bis, MNHN 1934-339, MNHN 1964-50, MNHN 2005-62, PIMUZ A/III 225, SMF 11615, SMF 11618, SMF 26579, SMF 32250, SMF 32909, SMF 33251, SMF 33252, SMF 33253, SMF 34427, SMF 46912, SMF 47171, SMF 53197, SMF 83055, SMF 83056, TMP 90.7.31, ZFMK 70424, ZFMK 14887, ZFMK 14888, ZFMK 21655, ZFMK 21656, ZFMK 7847

Varanus nuchalis – LACM 163944

Varanus ornatus – SMF 36173, SMF 54117, ZFMK 14889, ZFMK 87629

Varanus prasinus – SMF 11626, SMF 69454, ZFMK 14868, ZFMK 14869, ZFMK 14870, ZFMK 14871, ZFMK 14874, ZFMK 14875, ZFMK 54845, ZFMK 54846, ZFMK 76978, ZFMK 7929

Varanus rudicollis – MNHN 1973-108, SMF 40207, SMF 59216, SMF 59239, SMF 59242, SMF 67586, SMNS 14388, TMP 90.7.361, ZFMK 5229, ZFMK 53534, ZFMK 54253

Varanus salvator – MNHN 1886-284, MNHN 1888-198, MNHN 1977-04, PIMUZ A/III 0232, PIMUZ A/III 0249, PIMUZ A/III 1493, SMF 11563, SMF 32807, SMF 32908, SMF 33126, SMF 33127, SMF 33128, SMF 33129, SMF 33130, SMF 33131, SMF 33132, SMF 33133,

SMF 33134, SMF 35148, SMF 40175, SMF 40176, SMF 40177, SMF 40178, SMF 66647, SMF 69440, SMF 72158, SMF 81057, SMF 86676, SMF 90068, TMP 90.7.221, TMP 90.7.223, TMP 90.7.269, TMP 90.7.273, TMP 90.7.274, ZFMK 14859, ZFMK 14860, ZFMK 14861, ZFMK 21651, ZFMK 70190, ZFMK 70205, ZFMK 70433, ZFMK 91955

Varanus salvadorii – SMF 57878, SMF 58064, SMF 67670, ZFMK 90996, ZFMK 90997

Varanus semirex – ZFMK 54247, ZFMK 54248, ZFMK 54249

Varanus similis – ZFMK 54251, ZFMK 59027

Varanus spenceri – SMF 53277

Varanus storri – ZFMK 14880, ZFMK 54848, ZFMK 54849

Varanus timorensis – SMNS 1440, TMP 90.7.38, ZFMK 14886, ZFMK 10000

Varanus tristis – SMF 11630

Varanus varius – NHMW 1669, TMP 1997.030.0340
