# Supplementary material for: Revision of Varanus marathonensis (Squamata, Varanidae) based on historical and new material: morphology, systematics, and paleobiogeography of the European monitor lizards
Source: PLoS One. 2018 Dec 5;13(12):e0207719. doi: 10.1371/journal.pone.0207719 (PMC6281198; doi:10.1371/journal.pone.0207719)
Supplement: S2 File — (PDF) [file pone.0207719.s002.pdf]

List of references consulted to score characters for the phylogenetic analysis.

Augé M. Évolution des lézards du Paléogène en Europe. Mém Mus natl Hist nat Paris. 2005; 192: 1–369.

Borsuk-Białynicka M. Anguimorphans and related lizards from the Late Cretaceous of the Gobi Desert. Palaeontol Pol. 1984; 46: 5–105.

Brongersma LD. On an extinct species of the genus *Varanus* (Reptilia, Sauria) from the island of Flores. Zool Meded. 1958; 36: 113–125.

Caldwell MW, Cooper J. Redescription, palaebiogeography, and palaeoecology of *Coniasaurus crassidens* Owen, 1850 (Squamata) from the English Chalk (Cretaceous; Cenomanian). Zool J Linn Soc. 1999; 127: 423–452.

Caldwell MW. On the aquatic squamate *Dolichosaurus longicollis* Owen, 1850 (Cenomanian, Upper Cretaceous), and the evolution of elongate necks in squamates. J Vert Paleontol. 2001; 20: 720–735.

Caldwell MW. A new species of *Pontosaurus* (Squamata, Pythonomorpha) from the Upper Cretaceous of Lebanon and a phylogenetic analysis of Pythonomorpha. Mem Soc Ital Sci Nat Mus Civ Stor Nat Milano. 2006; 24: 1–42.

Clos LM. A new species of *Varanus* (Reptilia: Sauria) from the Miocene of Kenya. J Vert Paleontol. 1995; 15: 254–267.

Conrad JL. Skull, mandible, and hyoid of *Shinisaurus crocodilurus* Ahl (Squamata, Anguimorpha). Zool J Linn Soc. 2004; 141: 399–434.

Dutchak AR, Caldwell MW. Redescription of *Aigialosaurus dalmaticus* Kramberger, 1892, a Cenomanian mosasauroid lizard from Hvar Island, Croatia. Can J Earth Sci. 2006; 43: 1821–1834.

Fejérváry GJ de. A contribution to a monography on fossil Varanidae and on Megalanidae. Ann Mus Natl Hung. 1918; 16: 341–467.

Fejérváry GJ de. A further contribution to fossil Varanidae. Ann Mus Natl Hung. 1935; 29: 1–130.

Gao K, Norell MA. Taxonomic composition and systematics of Late Cretaceous lizard assemblages from Ukhaa Tolgod and adjacent localties, Mongolian Gobi Desert. Bull Am Mus Nat Hist. 2000; 249: 1–118.

Gilmore CW. Fossil lizards of North America. Mem Natl Acad Sci. 1928; 22: 1–201.

Ivanov M, Ruta M, Klembara J, Böhme. A new species of *Varanus* (Anguimorpha: Varanidae) from the early Miocene of the Czech Republic, and its relationships and palaeoecology. J Syst Palaeontol. 2018; 16: 767–797.

McDowell SB, Bogert C. The systematic position of *Lanthanotus* and the affinities of the anguinomorphan lizards. Bull Am Mus Nat Hist. 1954; 105: 1–142.

Molnar RE. Dragon in the Dust – The Paleobiology of the giant Monitor Lizard *Megalania*. Bloomington: Indiana University Press; 2004.

Norell MA, Gao K-Q, Conrad J. A new platynotan lizard (Diapsida: Squamata) from the Late Cretaceous Gobi Desert (Ömnögov), Mongolia. Am Mus Novit. 2008; 3605: 1–22.

Pianka ER, King DR, Allen King R. Varanoid Lizards of the World. Bloomington: Indiana University Press; 2004.

Rieppel O, Grande L. The anatomy of the fossil varanid lizard *Saniwa ensidens* Leidy, 1870, based on a newly discovered complete skeleton. J Paleontol. 2007; 81: 643–665.

Seeley HG. On remains of a small lizard from Neocomian rocks of Comen, near Trieste, preserved in the Geological Museum of the University of Vienna. Q J Geol Soc Lond. 1881; 37: 52–56.
