# Supplementary material for: Revision of Varanus marathonensis (Squamata, Varanidae) based on historical and new material: morphology, systematics, and paleobiogeography of the European monitor lizards
Source: PLoS One. 2018 Dec 5;13(12):e0207719. doi: 10.1371/journal.pone.0207719 (PMC6281198; doi:10.1371/journal.pone.0207719)
Supplement: S4 File — (PDF) [file pone.0207719.s004.pdf]

New characters added to the original list of Conrad et al. (2012).

6224. Groove on the lateral surface of the maxilla in the anterior sector of the bony narial opening: (0) absent; (1) present.

6225. Medial expansion on the anterior sloping edge of the facial process of the maxilla: (0) absent or weakly developed only in the lower sector of the sloping surface and not defined posteriorly (gradually merging with the lateral wall of the facial process); (1) present, developed on the ascending branch of the process, and well defined posteriorly (not merging with the lateral wall of the facial process).
