# Supplementary figures and images for: Revision of Varanus marathonensis (Squamata, Varanidae) based on historical and new material: morphology, systematics, and paleobiogeography of the European monitor lizards
Source: PLoS One. 2018 Dec 5;13(12):e0207719. doi: 10.1371/journal.pone.0207719 (PMC6281198; doi:10.1371/journal.pone.0207719)

## Strict consensus tree

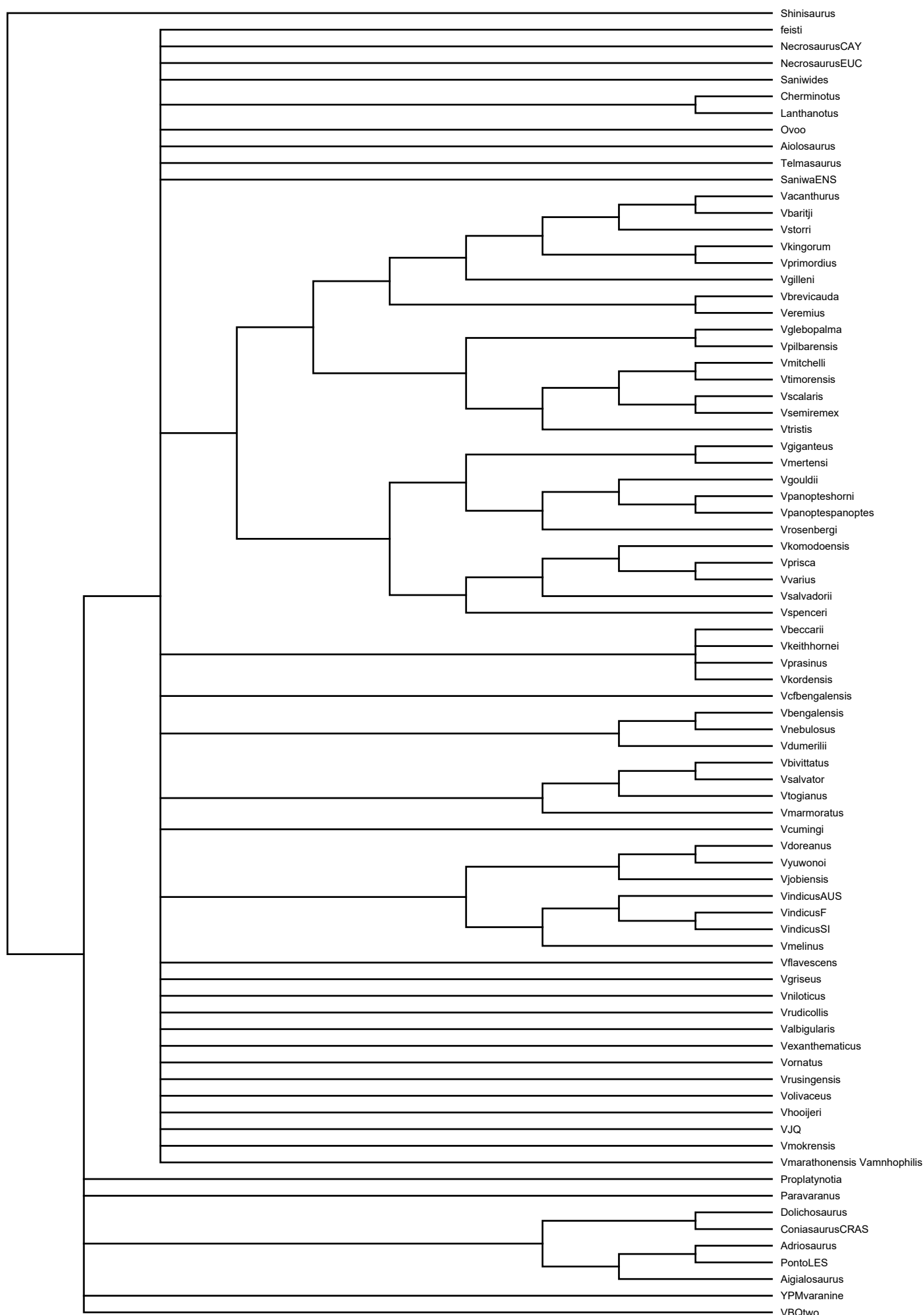

# Majority-rule consensus tree

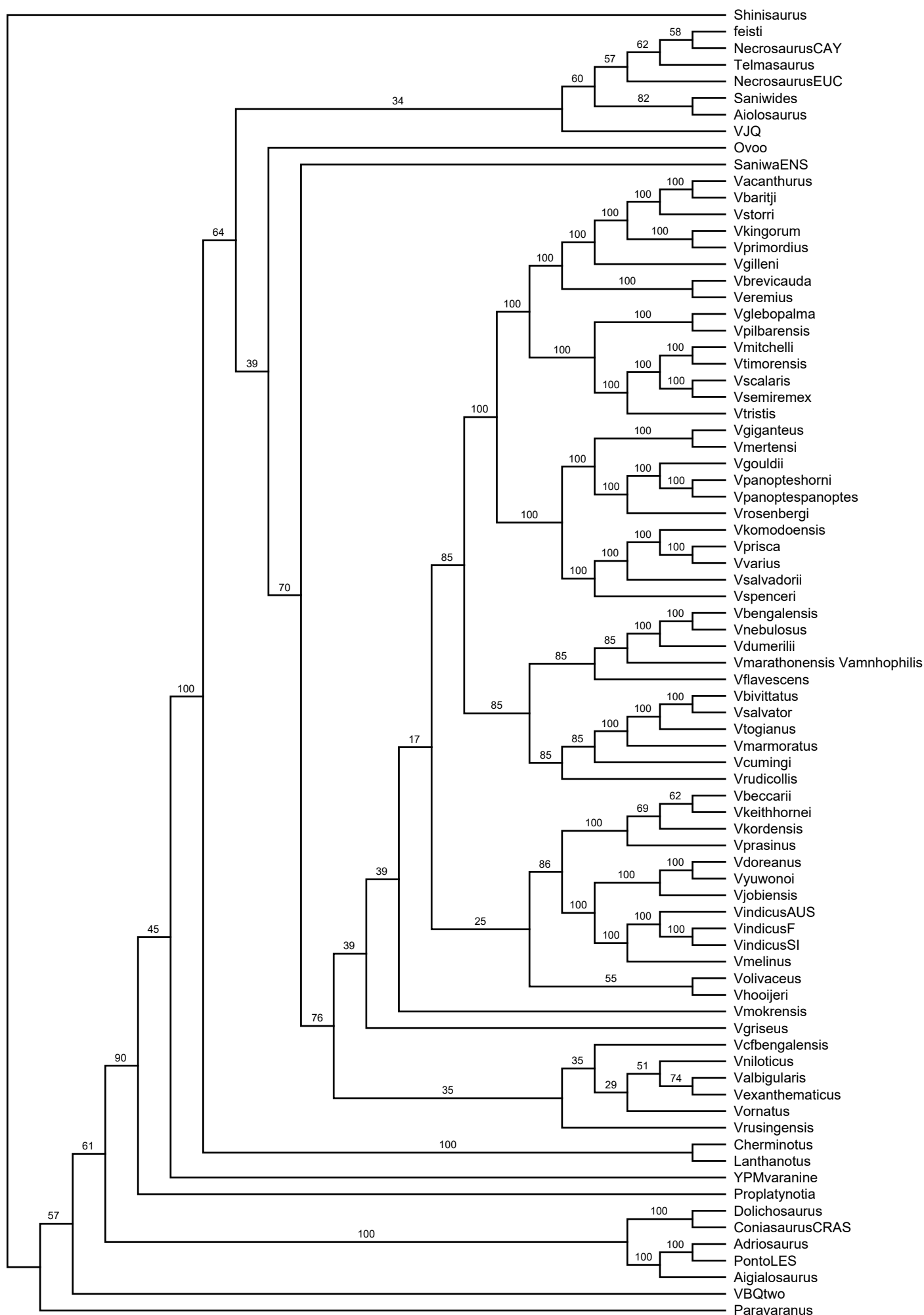

# Adams consensus tree

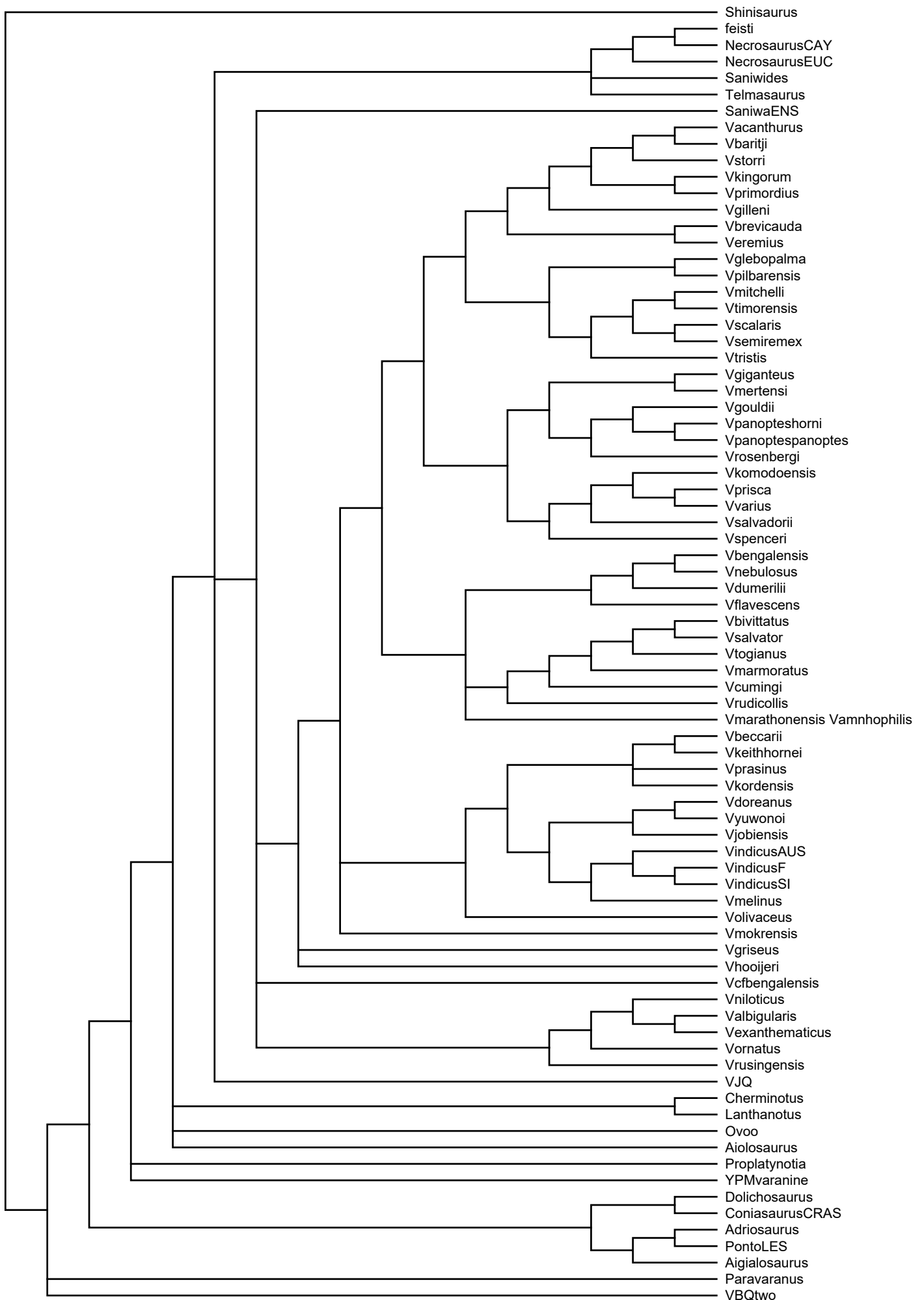

Supplement: S5 File — (PDF) [file pone.0207719.s005.pdf]

# Strict consensus tree

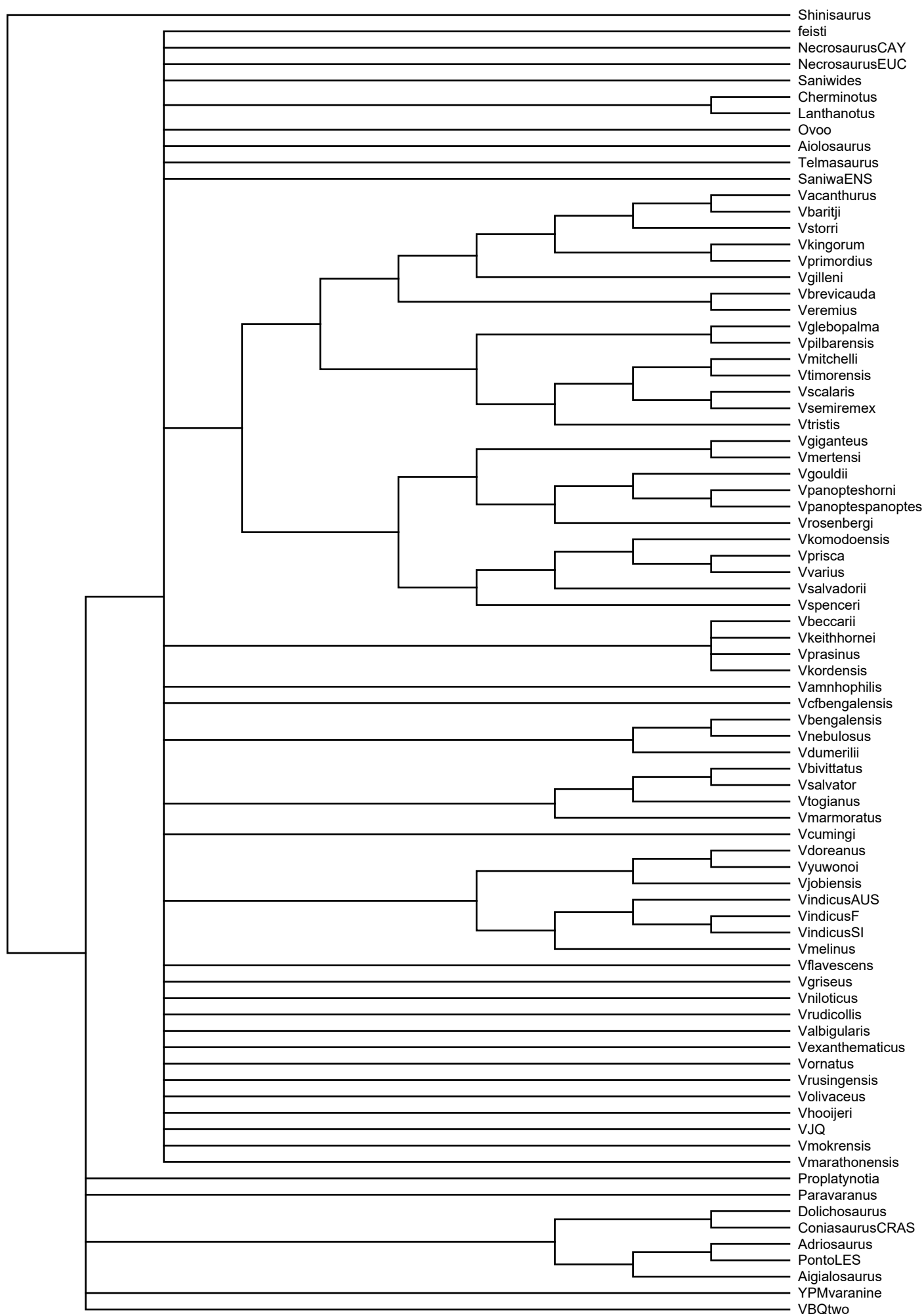

# Majority-rule consensus tree

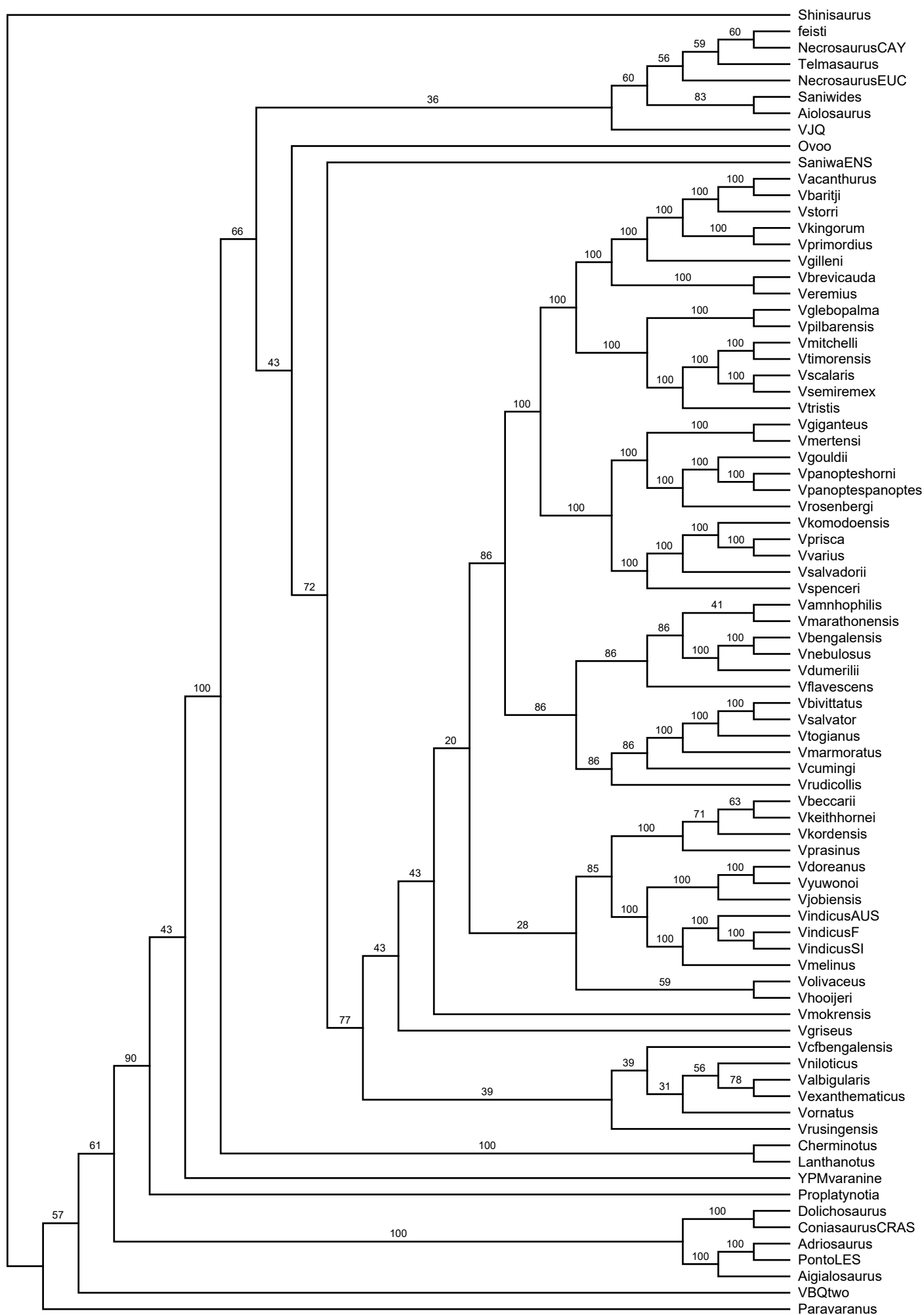

## Adams consensus tree

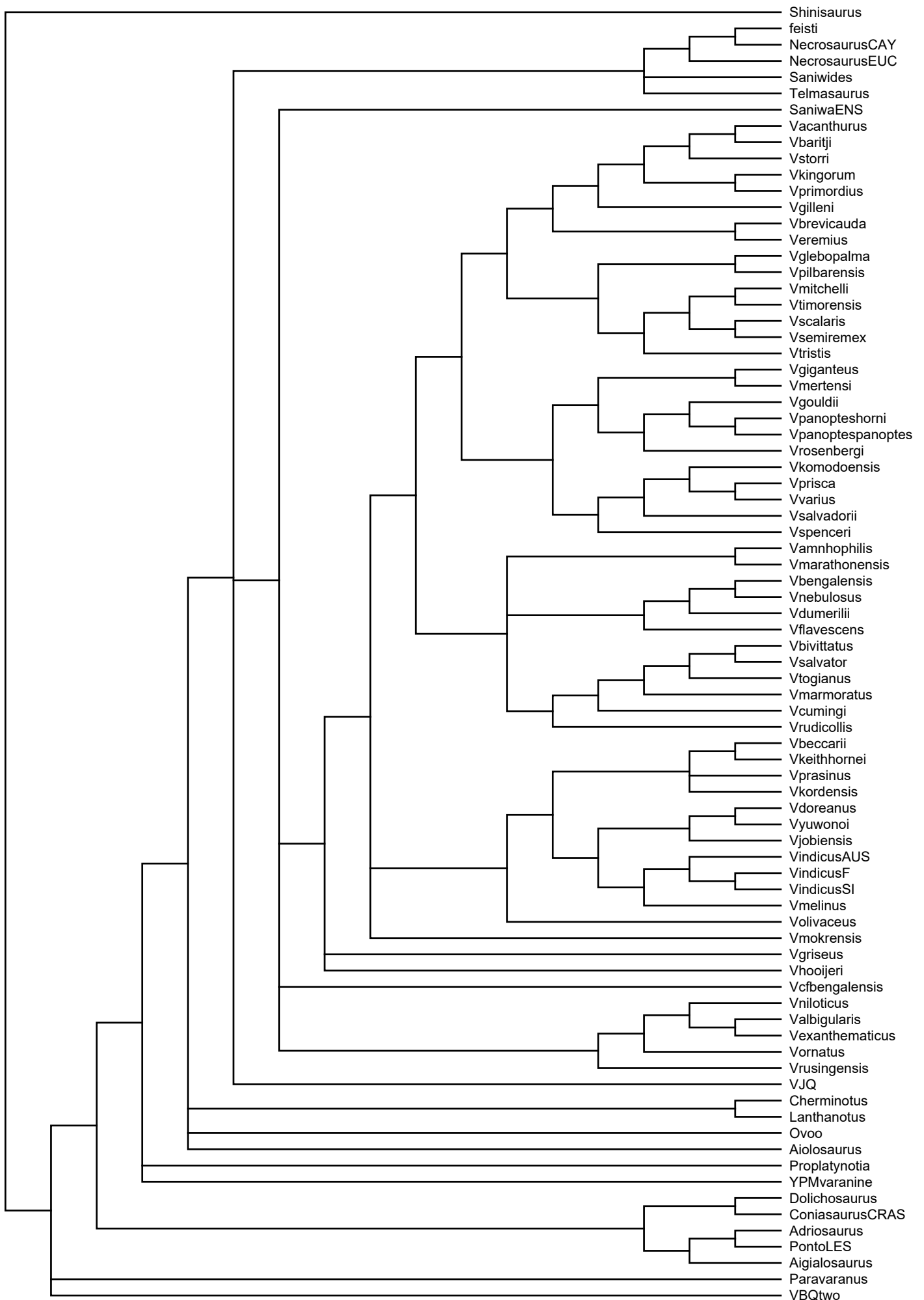

Supplement: S6 File — (PDF) [file pone.0207719.s006.pdf]
