# Supplementary material for: Revision of Varanus marathonensis (Squamata, Varanidae) based on historical and new material: morphology, systematics, and paleobiogeography of the European monitor lizards
Source: PLoS One. 2018 Dec 5;13(12):e0207719. doi: 10.1371/journal.pone.0207719 (PMC6281198; doi:10.1371/journal.pone.0207719)
Supplement: S7 File — (PDF) [file pone.0207719.s007.pdf]

Lists of the apomorphies of *V. marathonensis*, of the synapomorphies of the clade including *V. marathonensis* and the subgenus *Indovaranus*, and of *Varanus* (based on analysis 1B).

node_115 --> Vmarathonensis Vamnhophilis

82 1 0.111 0 ==> 1

131 1 1.000 0 ==> 2

141 1 0.167 1 ==> 0

385 1 0.333 0 ==> 1

397 1 0.500 0 ==> 1

6224 1 1.000 0 ==> 1

6225 1 1.000 0 ==> 1

node_116 --> node_115

128 1 0.143 0 ==> 1

173 1 0.143 1 --> 0

292 1 0.143 1 --> 0

384 1 0.167 0 --> 1

386 1 0.182 1 --> 2

399 1 0.143 0 --> 1

432	1	0.304	3 --> 1
451	1	0.154	0 --> 1
472	1	0.400	1 --> 2
478	1	0.222	2 --> 0
492	1	0.118	0 --> 2
500	1	0.273	0 --> 2
502	1	0.111	3 --> 1
504	1	0.286	1 --> 0
511	1	0.125	1 --> 3
514	1	0.333	3 --> 1
539	1	0.111	1 --> 3
563	1	0.111	1 --> 3
578	1	0.200	0 --> 2
641	1	0.133	1 --> 0
653	1	0.125	3 --> 1
707	1	0.200	1 --> 3
712	1	0.125	1 --> 3

722	1	0.111	1 --> 3
770	1	0.214	0 --> 3
773	1	0.083	1 --> 3
803	1	0.167	1 --> 3
818	1	0.167	1 --> 3
820	1	1.000	3 --> 1
854	1	0.167	1 --> 3
968	1	0.231	1 --> 3
971	1	0.222	1 --> 0
1139	1	0.077	1 --> 3
1142	1	0.111	1 --> 3
1178	1	0.125	1 --> 3
1212	1	0.250	1 --> 3
1283	1	0.154	1 --> 3
1337	1	0.300	0 --> 2
1349	1	0.083	3 --> 1
1409	1	0.214	1 --> 0

1422 1 0.200 1 --> 0

1430 1 0.091 1 --> 3

1469 1 0.143 3 --> 1

1554 1 0.429 1 --> 3

1559 1 0.200 1 --> 0

1622 1 0.273 3 --> 1

1640 1 0.077 1 --> 3

1646 1 0.214 0 --> 3

1700 1 0.250 1 --> 3

1734 1 0.143 0 --> 2

node_145 --> node_144 (*Varanus*)

12 1 0.111 1 ==> 2

69 1 0.250 0 ==> 1

72 1 0.111 1 --> 2

74 1 0.200 0 --> 1

115 1 0.500 2 --> 1

118	1	0.333	1 --> 2
235	1	0.500	1 ==> 0
393	1	0.333	1 --> 2
